
JOURNAL INFORMATION
==============================
NLM Title Abbreviation: Innov Surg Sci
Journal ID: Innov Surg Sci
NLM Journal ID: 101708165
Journal ID: iss

Innovative Surgical Sciences

EISSN: 2364-7485
Publisher: De Gruyter

ARTICLE INFORMATION
==============================
PMCID: PMC11917188
PMID: 40110103
DOI: 10.1515/iss-2025-2005
Article ID: iss-2025-2005
Article version: 1
Subjects: Article

Abstracts SCF

Electronic publication date: 2025 Mar 19
Publication date: 2025 Mar
Volume: 10
Issue: Suppl1
First page: 256
Last page: 367
Copyright: © 2025 by Walter de Gruyter Berlin/Boston
Copyright year: 2025
Copyright holder: De Gruyter
License: This work is licensed under the Creative Commons Attribution 4.0 International License.
License URL: https://creativecommons.org/licenses/by/4.0/

Page count: 112

==============================

Abstracts – DCK 2025 – 142nd Congress of the German Society of Surgery – Munich, March 26–28 • DOI 10.1515/iss-2025-2005
Innov Surg Sci 2025; 10 (Special Suppl 1): s256–s367

**Real-Time Monitoring of Pancreatic Tumor Dynamics: Insights from a 3D-in-vivo-model as an alternative preclinical model**
ID: 860

**Kategorie: SCF - Biomaterials**

Andreas Ettner-Sitter[1 2], Benedikt Wagner[1], Jonas Kuenzel[2], Agata Montagner[2], Monika Pizon[3], Rose Behncke[4], Rene Hägerling[4], Dieter Fuchs[5], Christoph Brochhausen-Delius[6], Rebecca Schönmehl[6], Thiha Aung[7], Silke Härteis[2], Christina Hackl[1]

[1]*Department of Surgery, University Hospital Regensburg , Regensburg, Deutschland*, [2]*Institute for Molecular and Cellular Anatomy, University of Regensburg, Regensburg, Deutschland*, [3]*Transfusionsmedizinisches Zentrum Bayreuth, Bayreuth, Deutschland*, [4]*Berlin Institute of Health at Charité-Universitätsmedizin Berlin, BIH Center for Regenerative Therapies, Berlin, Deutschland*, [5]*FUJIFILM VisualSonics, Inc., 1114 AB, Amsterdam, Niederlande*, [6]*Institute of Pathology, Medical Faculty Mannheim, Heidelberg, Deutschland*, [7]*Faculty of Applied Healthcare Science, Deggendorf Institute of Technology, Deggendorf, Deutschland*

**Background:**
Pancreatic ductal adenocarcinoma (PDAC) is a highly aggressive form of pancreatic cancer, accounting for about 85% of all pancreatic cancer cases and ranking as one of the deadliest cancers worldwide, characterized by increasing incidence rates and a 5-year survival rate of only 6%. This form of adenocarcinoma is accompanied by pronounced vascularization and compression of blood vessels. Angiogenesis, the formation of new blood vessels, plays a crucial role in PDAC progression. PDAC's lethality is driven by factors such as rapid progression, resistance to conventional therapies, and late diagnosis. A hallmark of PDAC is its dense, fibrotic tumor microenvironment (desmoplasia), which impedes blood flow, leading to hypoxia and poor drug delivery.
To investigate PDAC biology, the chorioallantoic membrane (CAM) model has emerged as a valuable preclinical tool. The CAM model, derived from chicken embryos, allows for the grafting of human tumor tissues, enabling real-time observation of tumor growth and vascularization. Advanced imaging techniques such as 3D ultrasonography and laser speckle contrast imaging have further enhanced the model's utility. Recent studies have demonstrated the successful cultivation of PDAC tissues and tumor spheroids in the CAM model, allowing for detailed insights into tumor progression and metastasis. This study aims to quantitatively analyze PDAC growth and angiogenesis in the CAM model using innovative imaging and histological techniques, advancing the understanding of PDAC biology and therapeutic interventions.

**Materials and methods:**
The CAM, a highly vascularized membrane in the chicken embryo, facilitates the transplantation of human tissues, including PDAC and tumor spheres, for *in vivo* observation. To monitor tumor growth and vascularization, 2D and 3D ultra-high frequency ultrasound imaging was conducted using the VisualSonic’s Vevo MD Imaging System with a 71 MHz transducer. Perfusion was assessed using laser speckle contrast imaging (LSCI), while volumetric measurements were obtained through 3D imaging with the PIUR tUS Infinity platform, reconstructing ultrasound volumes using AI-based algorithms. For histological examination, whole-mount immunofluorescence staining was conducted to visualize endothelial and smooth muscle tissue, thereby blood vessels, utilizing isolectin and nanobodies against CD31 and smooth muscle actin. Using CD31, specifically human endothelial tissue can be detected. The tissue was subsequently processed for histochemistry, including hematoxylin and eosin (H&E) staining, and Elastica Van Gieson (EVG) staining to investigate elastosis in vascular structures.

**Results:**
The 3D ultrasound technique allowed for reproducible quantification of tumor volume and surface area across multiple scans. Our results demonstrated significant reductions in tumor size observed over time, specifically at 24h, 72h, 120h, and 168h post-engraftment, indicating a dynamic response of the tumor

Open Access. © 2025 The Author(s), published by De Gruyter. This work is licensed under the Creative Commons Attribution 4.0 International License.

to the microenvironment. Furthermore, the ultra-high frequency (UHF) ultrasound imaging effectively detected anastomoses between chicken and human blood vessels, revealing a progressive increase in branching points throughout the 7-day experimental period. This highlights the potential for real-time monitoring of vascular development in a non-invasive manner. Observation of tumor sphere vascularization further revealed development of microvessels.

Complementary to previous findings, LSCI provides quantitative data on perfusion, showing a significant increase in perfusion units within the engrafted tumor tissues and tumor spheres, indicating enhanced vascularization over time. This particularly correlated with the emergence of new blood vessels, suggesting enhanced angiogenesis. Whole mount staining further confirmed the presence of anastomoses, providing visual evidence of the vascular connections formed. EVG staining illustrating fibrosis and collagen, indicated varying degrees of elastosis within the PDAC tissue, characterized by increased elastic fiber accumulation in blood vessels, which may correlate with the aggressive nature of PDAC. Additionally, immunohistochemical analyses identified blood and lymphatic vessels originating from human tissue.

**Conclusion:**

This study demonstrates the effectiveness of the CAM model for visualizing and quantifying key characteristics of pancreatic ductal adenocarcinoma, including tumor volume, angiogenesis, and perfusion dynamics. The integration of advanced imaging techniques, such as ultra-high frequency ultrasound and laser speckle contrast imaging, alongside immunohistochemical analyses, provides a comprehensive understanding of the tumor microenvironment. Overall, this study underscores the utility of ultrasound imaging as a robust and non-invasive method for monitoring tumor dynamics and vascularization in PDAC. The insights gained from this research have the potential to inform future therapeutic strategies aimed at targeting the tumor microenvironment and improving treatment outcomes for patients with this challenging cancer type.

### Approaches for the treatment of perforated peptic ulcers: a network meta-analysis of randomized controlled trials

ID: 165

**Kategorie: SCF - Clinical Studies**

Elisabeth Wadewitz[1], Juliane Friedrichs[1], Maurizio Grilli[2], Johannes Vey[3], Samuel Zimmermann[3], Yoshiaki Sunami[1], Jörg Kleeff[1], Ulrich Ronellenfitsch[1], Johannes Klose[1], Artur Rebelo[1]

[1]*University Hospital Halle (Saale), Department of Visceral, Vascular and Endocrine Surgery, Halle (Saale), Deutschland*, [2]*Library of the Medical Faculty Mannheim, Mannheim, Deutschland*, [3]*University Hospital Heidelberg, Institute of Medical Biometry, Heidelberg, Deutschland*

**Background:**

Perforated peptic ulcers are a severe, life-threatening condition, with high mortality and morbidity rates. Various treatment approaches are available, with differing outcomes in terms of effectiveness. This network meta-analysis (NMA) aims to compare surgical and alternative treatments concerning mortality and other patient-relevant outcomes.

**Materials and methods:**

A systematic literature search was conducted across PubMed/MEDLINE, Cochrane Library, Embase, CINAHL, ClinicalTrials.gov, and ICTRP using predefined search terms. To determine the most effective treatment approach, a network meta-analysis was performed for each outcome. Following treatments were compared: open surgical treatment, laparoscopic treatment, combined endoscopic and laparoscopic treatment, combined endoscopic and radiologic treatment (endoscopic stenting and repair with radiological fluid drainage) and conservative treatment. The standardized mean difference with a 95% confidence interval was used for continuous outcomes, while the odds ratios with 95% confidence intervals were calculated for binary outcomes.

**Results:**

This NMA included 949 patients from 12 randomized controlled trials (RCTs). The laparoscopic approach and the combined endoscopic and radiologic approach showed significant reductions in mortality rates (laparoscopic: OR 0.27; 95% CI 0.10 to 0.71; p=0.01; combined approach: OR 0.04; 95% CI 0.00 to 0.91; p=0.04) when compared to the open surgical treatment. The laparoscopic approach is represented by several studies while the combined endoscopic and radiologic approach is represented by one study. Concerning morbidity, the laparoscopic approach was associated with significantly lower risks of hernia (OR 0.15; 95% CI 0.04 to 0.53; p=0.003), wound infection (OR 0.13; 95% CI 0.06 to 0.28; p<0.0001), pneumonia (OR 0.30; 95% CI 0.09 to 0.98; p=0.05), and ileus (OR 0.32; 95% CI 0.15 to 0.66). The combined approach also showed a significantly reduced risk of hernia (OR 0.03; 95% CI 0.00 to 0.59; p=0.02) and pneumonia (OR 0.03; 95% CI 0.00 to 0.66; p=0.03).

**Conclusion:**

This NMA suggests that the laparoscopic approach is associated with lower mortality and reduced rates of complications such as pneumonia, wound infection, hernia, and ileus compared to the open surgical approach. The combined endoscopic and radiologic approach also shows potential benefits in reducing mortality and complications like hernia and pneumonia. Due to limited evidence, further RCTs are necessary to confirm whether the combined endoscopic and radiologic approach results in lower mortality and postoperative complications especially compared to the laparoscopic treatment.

### Early discontinuation of surgical randomized controlled trials: a registry-based study

ID: 185

**Kategorie: SCF - Clinical Studies**

Magdalena Holze[1], Yassine Ouaissa[2], Rosa Klotz[1], Christoph Michalski[1], Susan van Dieren[2], Patrick Bossuyt[3], Aneel Bhangu[4], Ewen Harrison[5], Marc Besselink[2]

[1]*Universitätsklinikum Heidelberg, Klinik für Allgemein-, Viszeral- und Transplantationschirurgie, Heidelberg, Deutschland*, [2]*Amsterdam University Medical Center, Department of Surgery, Amsterdam, Niederlande*, [3]*Amsterdam University Medical Center, Department of Clinical Epidemiology, Biostatistics and Bioinformatics, Amsterdam, Niederlande*, [4]*University of Birmingham, College of Medical and Dental Sciences, Birmingham, Großbritannien*, [5]*University of Edinburgh, Clinical Surgery, Edinburgh, Großbritannien*

**Background:**

Randomized controlled trials (RCTs) are the reference standard for evaluating surgical interventions and therefore key to evidence-based clinical decision-making. However, conducting surgical RCTs comes with unique challenges, including the learning curve, surgeon and patient preferences, small patient cohorts, blinding and challenges in patient recruitment. These issues contribute to a reported high rate of early trial discontinuation and low-quality reporting, leading to inefficient use of research resources and raising ethical concerns. Additionally, non-publication of RCTs exacerbates this problem by keeping results inaccessible, resulting in redundant research efforts and repetition of avoidable errors. A decade ago, one in five surgical RCTs were discontinued, mainly due to recruitment failures and one in three completed trials remained unpublished. Although the quality of surgical RCTs has improved over the past decade due to the rise of specialized high-volume surgical centers and advances in prospective trial registration, the current extent of prematurely terminated and non-published trials remains unknown. This study determines the rates of discontinuation and non-publication in surgical trials in the past decade, offering insights into underlying causes and potential solutions for improving research transparency and efficiency.

**Materials and methods:**

Surgical RCTs registered between 1 January 2018 and 31 December 2019 in the ClinicalTrials.gov database are analyzed. The advanced search facility is queried using the keyword “surgery”. Eligibility criteria are: studies registered as an RCT, investigating a surgical topic (all surgical specialties) in an adult population, with a study phase III, IV and not applicable for non-drug and non-medical devices trials. Studies with recruitment status completed, suspended, terminated and withdrawn are included according to the recruitment status definitions of the ClinicalTrials.gov database. A systematic search for study publication in peer reviews journals is carried out, and trial investigators contacted if reasons for discontinued and non-published trials are not accessible. Descriptive analysis is performed for prevalence, causes, and reporting of discontinued trials. Characteristics of discontinued versus completed trials are compared using a $\chi^2$ test, with $p < 0.05$ considered statistically significant. Odds ratios with 95% confidence intervals are calculated via uni- and multivariable logistic regression to examine the impact of the variables funding source, sample size, trial center, and trial scope on discontinuation and completion. Sensitivity analyses are performed to assess the effects of these variables on trials discontinued due to poor recruitment, non-published completed trials, and subspecialty gastrointestinal surgery.

**Results:**

Thus far, 4451 interventional studies have been identified by the systematic data base search for further screening. At the time of abstract submission (30 of September 2024) 1659/ 3352 studies were screened independently by two reviewers. Conflicts were resolved via consensus or a third reviewer. 660 of the screened studies were included, among them 533 completed and 127 discontinued studies.

The final results of the identified discontinued and non-published surgical RCTs will be presented at the DCK 2025 including respective reasons, the contribution of recruitment failures to this issue and further analysis on the impact of variables such as industry funding, sample size and multi-/ single-center studies on successful completion and publication.

**Conclusion:**
Early discontinuation of surgical RCTs hampers progress in the quality of surgical care. By assessing the status of prematurely terminated and completed non-published surgical RCTs, recommendations for researchers, clinicians and policy makers can be formulated to overcome these difficulties in conducting RCTs. The results of this study will enable surgical scientists towards a more efficient use of the available research capacities for future randomized controlled trials in the surgical field.

### A retrospective comparison of preoperative characteristics and surgical outcomes in benign IPMN lesions and IPMN Carcinoma

ID: 221

**Kategorie: SCF - Clinical Studies**

Athanasios Zamparas[1], Ughur Aghamaliyev[1], Simon Sirtl[2], Yannick Meyer[1], Gwendolin Seidel[1], Jens Werner[1], Bernhard W. Renz[1]

[1]*Klinikum der Universität München, Klinik für Allgemein-, Viszeral- und Transplantationschirurgie, München, Deutschland*, [2]*Klinikum der Universität München, Medizinische Klinik und Poliklinik 2, München, Deutschland*

**Background:**

Intraductal Papillary Mucinous Neoplasms (IPMNs) are cystic lesions of the pancreas that vary in their potential for malignant transformation, with some progressing to IPMN carcinoma (IPMN-Ca). Despite the distinct biological differences between these benign and malignant entities, both conditions are typically managed with similar surgical approaches, including tumor resection and lymphadenectomy. However, patients with IPMN-Ca often experience immunosuppression and cachexia due to the malignancy, which can result in delayed recovery, increased postoperative morbidity, and higher mortality rates. In contrast, although IPMNs are generally benign, their softer consistency may contribute to specific postoperative complications related to the pancreas. Given these differing characteristics, this study aims to compare the rates of postoperative complications in patients undergoing pancreatectomies for IPMNs versus those with IPMN-Ca.

**Materials and methods:**

Consecutive patients who underwent pancreaticoduodenectomy (PD) or distal pancreatectomy (DP) for IPMN or IPMN-Ca at our hospital between January 2014 and December 2023 were retrospectively identified. Patients who had total pancreatectomy (TP) or enucleation were excluded, as TP was predominantly performed for IPMN-Ca, while enucleation was only used for benign IPMN cases. Clinical data, including patient demographics, preoperative laboratory results, postoperative complications, length of hospital stay, and tumor histopathology, were collected. Patients were then stratified into the IPMN group and the IPMN-Ca group, and statistical analyses were performed to identify significant differences between these groups.

**Results:**

A total of 144 patients who underwent PD or DP for IPMN or IPMN-Ca between January 2014 and December 2023 were included in this retrospective study. Based on the final histopathological diagnosis, patients were categorized into two groups: low/high grade IPMN (n=97, 67%) and IPMN-Ca (n=47, 33%). The median age of patients was similar between both groups (72 vs. 73 years, p=0.1), with a nearly equal gender distribution (48% vs. 49% male, p=0.4). The median tumor size was significantly larger in IPMN-Ca patients (30 mm) compared to those with IPMN (21 mm, p=0.001). Vascular resection was more frequently required in IPMN-Ca cases (28% vs. 2%, p<0.001), which also correlated with higher estimated blood loss (median 600 mL vs. 460 mL, p=0.02). Analysis of preoperative laboratory parameters revealed that increased CA 19-9 levels (92U/ml vs 14 U/ml,p<0.001) and decreased hemoglobin ( 12,5 g/dl vs 13,95 g/dl, p<0.001) and albumin (3,8 g/dl vs 4,4 g/dl, p=0.02) levels were more commonly observed in patients with IPMN-Ca. The rate of postoperative pancreatic fistula (POPF) grade B/C was twice as frequent in the IPMN group (24% vs. 11%, p=0.02), while delayed gastric emptying (DGE) B/C was more prevalent in the IPMN carcinoma group (15% vs. 5%, p=0.02). 30-day mortality rate was higher in the IPMN carcinoma group (4% vs. 1%, p=0.03).

**Conclusion:**

This study highlights the differences in postoperative outcomes between IPMNs and IPMN carcinomas, suggesting the need for tailored management strategies. Although IPMNs are generally benign, they are associated with a higher incidence of POPF grade B/C due to the soft texture of the pancreas. In contrast, while IPMN-Ca cases have a lower rate of POPF grade B/C, they experience DGE B/C approximately 2.5 times more frequently. This underscores the importance of careful postoperative management, particularly regarding the use of gastric tubes. Furthermore, despite a lower incidence of POPF grade B/C, IPMN-Ca patients exhibit significantly higher 30-day mortality rates. This increased mortality is attributed to factors such as immune suppression, malnutrition, and cachexia, highlighting the need for thorough preoperative evaluation and prehabilitation in these patients.

### Risk factors for recurrence after laparoscopic inguinal hernia repair – multivariate analysis within the randomized, multicenter, self-controlled BIOLAP trial

ID: 240

**Kategorie: SCF - Clinical Studies**

C. Simone Seefeldt[1], Judith Knievel[1], Muneer Deeb[2], Niels-Torsten Hoedt[3], Claudia Rudroff[4], Jonas Lange[1], Jürgen Meyer[1], Claus Ferdinand Eisenberger[1], Rolf Lefering[5], Markus Maria Heiss[1]

[1]*Kliniken der Stadt Köln, Klinikum der Universität Witten/Herdecke, Klinik für Viszeral-, Tumor-, Transplantations- und Gefäßchirurgie, Köln, Deutschland*, [2]*Ammerland Klinik Westerstede, Klinik für Allgemein- und Viszeralchirurgie, Westerstede, Deutschland*, [3]*Ev. Diakonissenkrankenhaus Leipzig, Klinik für Allgemein- und Viszeralchirurgie, Leipzig, Deutschland*, [4]*Evangelisches Klinikum Köln Weyertal, Abteilung für Viszeralchirurgie und funktionelle Chirurgie unterer Gastrointestinaltrakt, Köln, Deutschland*, [5]*Institut für Forschung in der Operativen Medizin, Universität Witten/Herdecke, Köln, Deutschland*

**Background:**
Inguinal hernia repair with synthetic mesh implantation is one of the most common operations worldwide. A remaining problem after hernia surgery are recurrences. There is raising evidence that absorbable meshes could be advantageous concerning occurrence of chronic pain as well as recurrences due to a different postoperative remodeling. The BIOLAP trial compared the use of synthetic and biological meshes in laparoendoscopic inguinal hernia repair.

**Materials and methods:**
491 patients with bilateral inguinal hernias were treated in TEP or TAPP technique in 21 German hernia centers. According to randomization, each patient received a biological implant on one side, left or right, and a synthetic mesh on the other. Patients were not informed about the location of each implant type. Pain intensity after 6 months and hernia recurrence 2 years post-surgery were defined as primary endpoints and recorded individually for all 982 hernia repairs among other secondary endpoints. A multivariate analysis identified influencing factors for recurrences.

**Results:**
After 2 years, the recurrence rate was significantly higher ($p<0.001$) for biological implants with 11.2 %, compared to 2.5 % for synthetic meshes. Within the multivariate analysis factors like patient characteristics, surgical factors, mesh material or size of hernia were analyzed. The implantation of biological mesh material is the most influencing factor for recurrences ($p<0.001$). A further significant risk factor is the operation in a low-volume center ($p <0.001$). With an odds ratio ≥ 1.4 other potentially influencing risk factors are patient age, EHS grade 3 size, medial hernia configuration and occurrence of seroma.

**Conclusion:**
The study provides multicenter data from 982 hernia operations dependent of the mesh material used. Occurrence of recurrences is dependent on mesh materials and amount of performed surgeries.

Abstracts – DCK 2025 – 142nd Congress of the German Society of Surgery – Munich, March 26–28 • DOI 10.1515/iss-2025-2005
Innov Surg Sci 2025; 10 (Special Suppl 1): s256–s367

**PAID trial – Prophylactic active irrigation-drainage of pancreatic stump vs. drainage only in high-risk patients after distal pancreatectomy: study protocol of a prospective randomized controlled clinical study**
ID: 283

**Kategorie: SCF - Clinical Studies**

Ilaria Pergolini[1], Daniel Reim[1], Stephan Schorn[1], Helmut Friess[1], Ihsan Demir[1]

[1]*Klinik un Poliklinik für Chirurgie, Klinikum rechts der Isar der Technischen Universität München, München, Deutschland*

**Background:**
Postoperative pancreatic fistulas (POPF) represent the most relevant issue after distal pancreatectomy. A clinically relevant POPF (grade B/C), as defined by the international study group for pancreatic surgery (ISGPS), leads to other severe postoperative complications, such as intraabdominal fluid collection, delayed gastric emptying (DGE), and bleeding. These complications cause prolongation of hospital stay, need for additional diagnostic and therapeutic procedures, and remarkably increased mortality rate. In the current literature, the incidence of clinically relevant POPF after distal pancreatectomy affects around 25% of patients and up to 30% of patients incur major complications. In patients at high-risk for POPF, this rate is even higher, reaching up to 60%. Continuous irrigation of the peripancreatic area is a well-known and established treatment strategy for the management of acute infected necrotizing pancreatitis and several studies demonstrated its effectiveness and safety. By contrast, its use after elective pancreatic surgery is still under investigation. In the systematic review that we conducted, this was described by a few research groups and showed promising results in reducing the POPF rate and subsequent complications. More recently, the use of prophylactic continuous irrigation after pancreaticoduodenectomy has been already studied in two randomized controlled trials that showed promising results. Two other randomized controlled trials on continuous irrigation after pancreaticoduodenectomy are currently ongoing, while no randomized controlled trials on the use of this procedure after elective distal pancreatectomy have been conducted or registered yet.

**Materials and methods:**
PAID is a multicenter, randomized, controlled surgical trial comparing prophylactic continuous irrigation and drainage of pancreatic stump vs. drainage only in high-risk patients after distal pancreatectomy. Patients were defined at high-risk for POPF (with a risk > 25%) based on the validated preoperative Distal Pancreatectomy Fistula Risk Score (D-FRS). The primary endpoint of this trial is the postoperative rate of major complications (Clavien-Dindo score ≥ 3), and the most relevant secondary outcome is grade B/C POPF. Other secondary outcomes include abdominal fluid collections, DGE, postpancreatectomy hemorrhage (as defined by ISGPS), wound infection, reoperations and reinterventions, quality of life, length of stay, readmission within 90 days, in-hospital mortality, and 90-day mortality. For statistical analysis, a binary logistic regression model will be used. With a level of significance of 5% and a power of 80%, a sample size of 71 patients per group has been identified as necessary.

**Results:**
None

**Conclusion:**
The findings of this trial will help to evaluate the usefulness of prophylactic continuous irrigation of the pancreatic stump at reducing the rate of postoperative major complications in patients at high-risk for POPF after distal pancreatectomy. The results could influence the postoperative management after distal pancreatectomy with potential impact on future clinical guidelines and practice.

**Delayed hepatic failure as a common and underappreciated mechanism of post-hepatectomy mortality**
ID: 305

**Kategorie: SCF - Clinical Studies**

Jonas Santol[1 2 3], Jack W. Sample[4], Joel Probst[5], Guido Costa[6], Markus Ammann[2 7], George Kaoukabani[2], Severin Gloor[8], Eva Braunwarth[9 10], Alessandro Fogliati[11], Pieter Arntz[12], Jan P. Jonas[13], Vanja Podrascanin[2 14], Anna S. Jankoschek[15], Anna E. Kern[15], Anina Schmid[16], Silvia Gasteiger[17], Felix J. Bussmann[13], Oscar Salirrosas[18], Gregor Ortmayr[19], Yawen Dong[1 2], Stella K. Adjei Antwi[20], Florian Primavesi[9 10 21], Stefan Staettner[21], Eduardo A. Vega[18], Rafael Díaz-Nieto[22], Matthias Ilmer[2 23], Mattia Garancini[11], Fabrizio Romano[11 20], Joris Erdman[12], Susanne G. Warner[2], Marc J. Truty[2], Friedrich Laengle[7], Stefan Schneeberger[24], Guido Beldi[8], Rory L. Smoot[2], David M. Nagorney[2], Guido Torzilli[25], Thomas Gruenberger[26], Patrick Starlinger[2 27 28]

[1]*Klinik Favoriten - Wiener Gesundheitsverbund, ehemaliges Kaiser Franz Josef Spital, Abteilung für Chirurgie, Wien, Österreich*, [2]*Mayo Clinic, Department of Surgery, Rochester, Vereinigte Staaten*, [3]*Medizinische Universität Wien, Institut für Gefäßbiologie und Thromboseforschung, Wien, Österreich*, [4]*Mayo Clinic Rochester, Department of Surgery, Rochester, Vereinigte Staaten*, [5]*Klinik Favoriten, Abteilung für Chirurgie, Wien, Österreich*, [6]*IRCCS Humanitas Research Hospital, Rozzano, Italien*, [7]*Landesklinikum Wiener Neustadt, Abteilung für Chirurgie, Wiener Neustadt, Österreich*, [8]*Inselspital, Universitätsspital Bern, Abteilung für Chirurgie, Bern, Schweiz*, [9]*Medizinische Universität Innsbruck, Abteilung für Chirurgie, Innsbruck, Österreich*, [10]*Daniel Swarovski Research Laboratory, Department of Surgery, Innsbruck, Österreich*, [11]*Fondazione IRCCS San Gerardo dei Tintori, Department of Surgery, Monza, Italien*, [12]*Academisch Medisch Centrum, Department of Surgery, Amsterdam, Niederlande*, [13]*Universitätsspital Zürich, Department of Surgery, Zürich, Schweiz*, [14]*Landesklinikum Wiener Neustadt, Department of Surgery, Wiener Neustadt, Österreich*, [15]*Medizinische Universität Wien, Wien, Österreich*, [16]*Inselspital, Universitätsspital Bern, Department of Surgery, Bern, Schweiz*, [17]*Medizinische Universität Innsbruck, Department of Surgery, Innsbruck, Österreich*, [18]*St. Elizabeth's Medical Center, Department of Surgery, Boston, Vereinigte Staaten*, [19]*Medizinische Universität Wien, Center for Cancer Research, Wien, Österreich*, [20]*Università degli Studi di Milano Bicocca, Department of Surgery, Milano, Italien*, [21]*Salzkammergut Klinikum Vöcklabruck, Department of Surgery, Vöcklabruck, Österreich*, [22]*Aintree University Hospital, Department of Surgery, Liverpool, Großbritannien*, [23]*Ludwig-Maximilians-Universität München, Abteilung für Chirurgie, München, Deutschland*, [24]*Medizinische Universität Innsbruck, Abteilung für Chirugie, Innsbruck, Österreich*, [25]*Humanitas Research Hospital, Department of Surgery, Cascina Perseghetto, Italien*, [26]*Klinik Favoriten - Wiener Gesundheitsverbund, ehemaliges Kaiser Franz Josef Spital, Abteilung Für Chirurgie, Wien, Österreich*, [27]*Medizinische Universität Wien, Universitätsklinik für Allgemeinchirurgie, Wien, Österreich*, [28]*Medizinische Universität Wien, Zentrum für Physiologie und Pharmakologie, Wien, Österreich*

**Background:**
Posthepatectomy liver failure (PHLF) remains the greatest risk factor for postoperative death in patients undergoing liver surgery accounting for approximately 50% of all 90-day postoperative mortality. Assessing patient outcomes after hepatic resection, we recognized that a significant proportion of patients died within the first postoperative year from what appeared to be delayed hepatic decompensation after the classical 90-day observation period. This study aimed to investigate the exact causes of death within the first year after liver surgery and to characterize the phenomenon of delayed hepatic failure (DHF) and its associated symptoms.

**Materials and methods:**
7950 patients from 13 international centers were evaluated. Inclusion criteria were any type of liver resection, irrespective of indication and a follow up-period of one year or more. Depending on their date of death (dod), patients were divided into an immediate postoperative mortality group (dod ≤ 2

weeks postop), a 90-day mortality group (dod > 2 weeks and ≤ 90 days postop) and a one-year mortality group (dod > 90 days and ≤ 1 year postop). For these patients the cause of death (COD) was retrospectively evaluated. DHF was either defined via clinical documentation or as the combination of several symptoms associated with liver failure, such as ascites, fever, hepatic encephalopathy, jaundice, or significantly elevated liver function tests (LFT). Tumor recurrence and extent were assessed by postoperative surveillance imaging.

**Results:**
815 patients (10%) expired in the first year following hepatic resection. Of the patients who experienced mortality within the first year after surgery, 13% were immediate postoperative deaths and 21% were within 90 days after surgery (Fig. 1A). 66% of all 815 one-year mortalities occurred after 90 days post hepatectomy (Fig. 1A). Of this 66%, 34% of patients died at home with no retrospectively accessible COD documented (Fig. 1B). Of note, the majority (66%) of patients with assessable COD after 90 postoperative days were caused by liver failure (Fig. 1C). Of these only 17% were caused by hepatic tumor progression, while the remaining 83% of patients were experienced DHF (Fig. 1D). In a significant number of patients, DHF was secondary to infectious complications or sepsis (34%), ultimately leading to multi-organ failure. Finally, we wanted to define which symptoms developed during postoperative follow-up that might identify patients at risk for DHF. Patients ultimately suffering from DHF also suffered from jaundice (12%) or developed hepatic encephalopathy (20%) during the postoperative follow up period prior to progressing to fulminant liver failure. However, the most prominent symptom developing in the time between surgery and DHF was ascites. 55% of patients developing DHF had evidence of ascites in the postoperative follow-up period (Fig. 1E), with 29% requiring recurrent interventions for ascites management. Of the patients who developed ascites, 49% required medical management (diuretics) following hospital discharge after surgery. 22% of patients who developed DHF and did not present with clinically documented ascites were treated with diuretics as well. Interestingly, while the ISGLS definition of PHLF was associated with immediate postoperative mortality (64%) and 90-day mortality (55%), DHF was not captured by this definition (13%) (Fig. 1F).

**Conclusion:**
This study shows that DHF represents a common and under-reported phenomenon representing the most frequent mechanism of post-hepatectomy mortality. DHF often occurs outside of the routine 90-day follow-up after surgery and seems to be associated with chronic immunosuppression leading to infectious complications that ultimately result in multi-organ failure and death. The most significant objective risk factor we found for DHF was ascites, presumably indicating ongoing unstable liver function. These findings suggest that our current postoperative screening methods for patients undergoing liver resection surgery may fail to identify patients at risk for DHF and concomitantly fail to predict development of this common COD of this patient cohort. A stringent follow-up exceeding the routine 90-day period in high-risk patients, particularly in those developing ascites after liver surgery, could potentially decrease 1-yer mortality following liver surgery.

Abstracts – DCK 2025 – 142nd Congress of the German Society of Surgery – Munich, March 26–28 • DOI 10.1515/iss-2025-2005
Innov Surg Sci 2025; 10 (Special Suppl 1): s256–s367

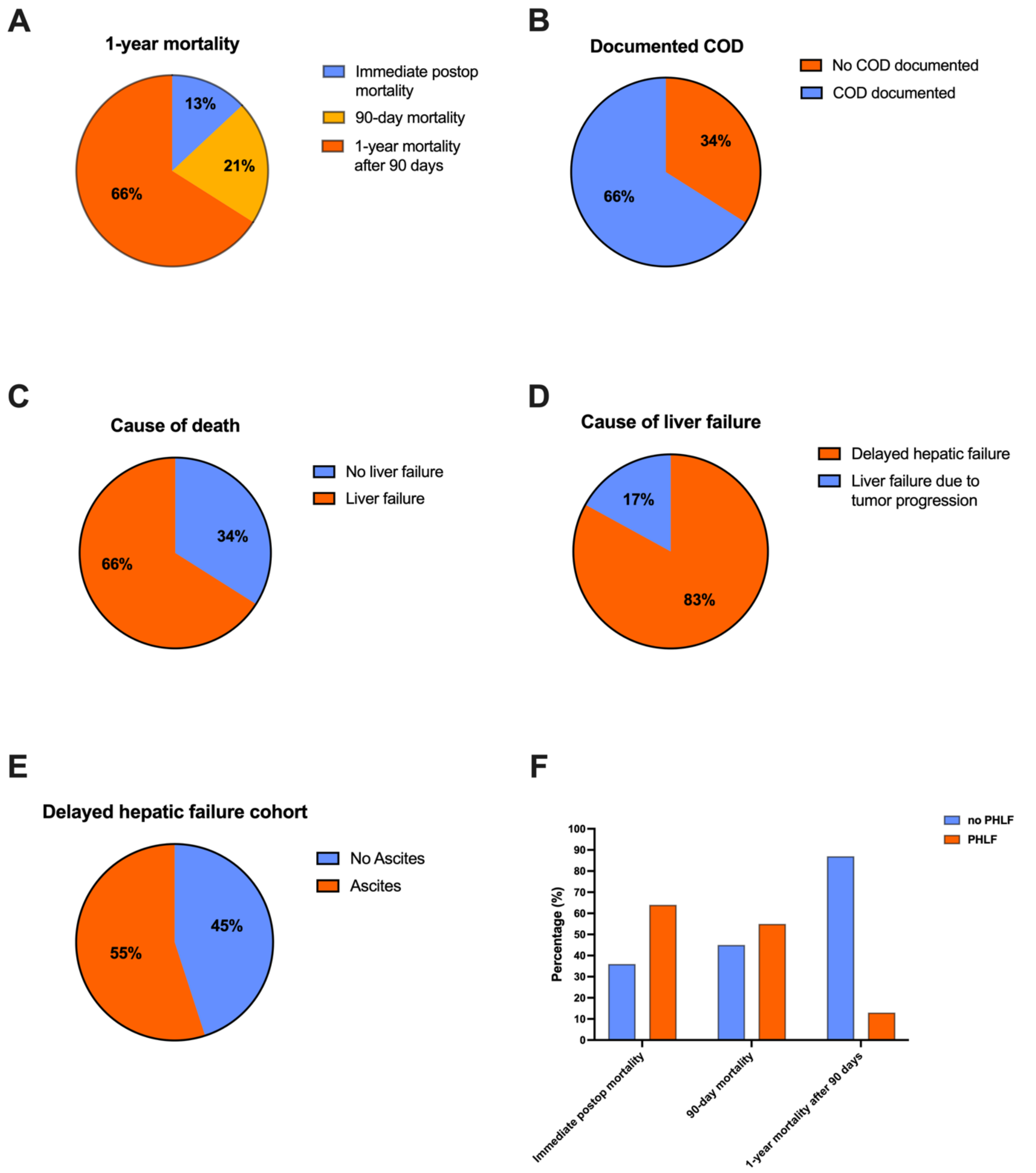

Fig. 1 (A) Type of 1-year mortality grouped by time until death as immediate postoperative mortality (≤2weeks), 90-day mortality (≤90 days) and 1-year mortality after 90days. (B) Percentage of patients were a cause of death (COD) was assessable. (C) Percentage of patients with liver failure as the COD. (D) Percentage of either delayed hepatic failure or liver failure caused by tumor progression as the cause of liver failure. (E) Percentage of patients developing ascites in the cohort of patients which died of delayed hepatic failure. (F) Percentage of patients who developed posthepatectomy liver failure grouped by their time until mortality.

**Pretherapeutic understaging in localized upper gastrointestinal cancers - a common clinical problem**
ID: 342

**Kategorie: SCF - Clinical Studies**

Johannes Moritz Riebeling[1], Martin Leu[2], Jannis Hagenah[1], Ahmad Amanzada[3], Volker Ellenrieder[4], Michael Ghadimi[1], Alexander König[4], Marian Grade[1]

[1]*Klinik für Allgemein- Viszeral- und Kinderchirurgie, Göttingen*, [2]*Universitätsmedizin Göttingen Klinik für Strahlentherapie und Radioonkologie , Göttingen*, [3]*Universitätsmedizin Göttingen Klinik für Gastroenterologie und gastrointestinale Onkologie, Göttingen*, [4]*Universitätsmedizin Göttingen Klinik für Gastroenterologie und gastrointestinale Onkologie, Göttingen*

**Background:**
Accurate pretherapeutic staging of upper gastrointestinal cancers is essential for treatment planning. In this respect, endoscopic ultrasound plays a key role in assessing local tumor infiltration and potential lymph node involvement. In locally advanced tumors, neoadjuvant (perioperative) treatment remains the gold standard, but there is no clear guideline recommendation for patients with cT2 cN0 tumors. The aim of this study was to evaluate the accuracy of endoscopic ultrasound for pretherapeutic staging of upper gastrointestinal cancers with a specific focus on cT2 cN0 cancers.

**Materials and methods:**
We conducted a retrospective analysis of patients with esophageal cancer (EC), cancers of the gastroesophageal junction (GEJ) and gastric cancer (GC), who underwent primary surgical resection at the Department of General, Visceral and Pediatric Surgery at the University Medical Center Göttingen between 2014 and 2024. Inclusion criteria were defined as follows: cancers that were pretherapeutically staged using endoscopic ultrasound as cT2 cN0, without evidence of distant metastasis (based on CT scans). Exclusion criteria were defined as follows: patients without pretherapeutical endoscopic ultrasound, patients with cancers other than cT2 cN0, and patients who received neoadjuvant treatment. Pretherapeutic endoscopic ultrasound staging was compared with definitive histopathological assessment after surgical resection.

**Results:**
In total, 45 patients (12 female, 33 male) with cT2 cN0 cancers were identified. The vast majority of tumors were histopathologically confirmed adenocarcinomas (42/45), while three tumors were classified as squamous cell carcinomas. An understaging was observed for 21 of 45 patients (46,6%). 15 of these 21 patients were male, six patients were female. Correct staging had been performed in 24 cases. In most patients whose tumors were understaged by endoscopic ultrasound, both T- and N-stage were underestimated (14/21). In three out of 21 patients, only the T-stage was incorrect, while the N-stage was underestimated in four out of 21 patients. Of note, understaging appeared nearly as often in patients with gastric cancers (11/21) as in patients with cancers of the GEJ (9/21). Only one patient with esophageal cancer was understaged. Nine understaged tumors were classified as G2, 12 understaged tumors as G3. All understaged carcinomas were histopathologically confirmed adenocarcinomas. The mean BMI of understaged patients was 25,8 compared to 26,7. The mean time interval between pretherapeutic endosonography and surgery was 13,1 days in understaged patients compared to 10.6 days in patients with correct endoscopic staging.

**Conclusion:**
Almost half of the patients with cT2 cN0 cancers were understaged by pretherapeutic endoscopic ultrasound. In particular patients with gastric or GEJ cancers were at a high risk of understaging. As a consequence, therapeutic options (primary surgical resection versus neoadjuvant treatment strategies) should be critically discussed in the respective tumor boards as well as with affected patients. This is particularly important because neoadjuvant treatment has been shown to be more effective than adjuvant regimes (in case of understaging).

**Advanced age, weight loss, and portal vein/ superior mesenteric vein resection are independent risk factors for delayed gastric emptying after distal pancreatectomy**
ID: 356

**Kategorie: SCF - Clinical Studies**

Jingcheng Zhang[1], Helmut Friess[1], Ihsan Ekin Demir[1], Florian Scheufele[1]

[1]*Department of Surgery, Klinikum rechts der Isar, School of Medicine and Health, Technical University of Munich, München, Deutschland*

**Background:**
Few studies have examined delayed gastric emptying (DGE) following distal pancreatectomy (DP). This study aimed to assess the incidence of DGE after DP and its impact on short-term outcomes and identify predictive risk factors.

**Materials and methods:**
All adult patients who underwent DP and pancreaticoduodenectomy (PD) at our hospital were consecutively enrolled from February 2017 to April 2023. Up to 58 variables were analyzed using univariate and multivariate analysis.

**Results:**
Among 162 DP patients, 12.35% developed DGE postoperatively, comparable to that after PD (13.88%, $P>0.05$). DGE patients had a significantly longer postoperative stay [22.50 (17.75-40.00) vs 14.00 (11.00-20.00) days, $P<0.001$] and a higher Clavien Dindo degree ≥3 (55% vs 33.10%, $P=0.043$). Specifically, the postoperative length of stay in patients with grade A DGE was still markedly increased than in patients without any complications [18.50 (13.50-23.25) vs 12.00 (10.00-15.00), $P=0.0208$]. The multivariate analysis identified age≥80 years [OR 4.457, 95% CI 1.187-16.738, $P=0.027$], unintentional weight loss (UWL)>10% body weight [OR 6.397, 95% CI 1.502-27.243, $P=0.012$], and portal vein (PV)/superior mesenteric vein (SMV) resection [OR 9.198, 95% CI 1.768-47.857, $P=0.008$] as independent risk factors for DGE after DP. Notably, there was no association between postoperative pancreatic fistula (POPF) and DGE after DP

**Conclusion:**
DGE is a frequent and under-estimated complication after DP, prolonging postoperative hospitalization, and more emphasis should be placed on recognizing even milder DGE. While reducing POPF may have little impact on DGE after DP, managing preoperative weight loss may help prevent its occurrence.

Abstracts – DCK 2025 – 142nd Congress of the German Society of Surgery – Munich, March 26–28 • DOI 10.1515/iss-2025-2005
Innov Surg Sci 2025; 10 (Special Suppl 1): s256–s367

### Incidental Findings of Pancreatic Pathologies in Preoperative MRCP for Cholecystectomy

ID: 526

**Kategorie: SCF - Clinical Studies**

Jasmin Zeindler[1 2], Gabriel Fridolin Hess[2], Simone Muenst[3], Martin Bolli[2 4], Savas Deniz Soysal[5], Otto Kollmar[1 2]

[1]*Klinik Chirurgie & Viszeralchirurgie, Kantonsspital Baselland, Liestal, Schweiz*, [2]*Clarunis Universitäres Bauchzentrum Basel Viszeralchirurgie, Basel, Schweiz*, [3]*Universitätspital Basel Pathologie, Basel, Schweiz*, [4]*Allgemein- & Viszeralchirurgie - Luzerner Kantonsspital, Luzern 16, Schweiz*, [5]*Medizinische Fakultät* | *Universität Basel, Basel, Schweiz*

**Background:**
Preoperative magnetic resonance cholangiopancreatography (pMRCP) is commonly used to exclude choledocholithiasis and to have a better understanding of patient's anatomy prior to cholecystectomy. Pancreatic pathologies often represent incidental findings in these preoperative imaging studies. We investigated a large cohort of patients with pMRCP for cholecystectomy and analysed the occurrence of incidental pancreatic pathologies.

**Materials and methods:**
This retrospective cohort study consecutively included all patients undergoing pMRCP for cholecystectomy between 2010 and 2020. The occurrence of incidental pancreatic pathologies have been recorded as well as their impact on further surveillance and diagnostic has been analysed.

**Results:**
In the given time period, 2267 patients underwent MRCP prior to cholecystectomy. In 542/2267 patients (24%) pancreatic pathologies have been observed. The most common pancreatic pathologies were pancreatic cysts (171/542, 32%) and radiological signs of pancreatitis (50/542, 9%). Intraductal papillary mucinous neoplasia (IPMN) were diagnosed in 15 cases (3%), and 12 (80%) of these were branch-duct-IPMN. 8 patients showed duodenal diverticula (1.5%), 8 patients with atrophy of the pancreas (1.5%), 6 patients had lipomatosis (1%), 5 ductal ectasia (1%), and 5 patients had pancreatolithiasis (1%). In addition, 11 (2%) patients were diagnosed with solid tumours of which 10 were unspecific lesions, which were followed up with imaging in 9 patients and with endoscopic ultrasound +/- biopsy in one patient. The remaining patient with tumour was radiologically suspicious and was diagnosed as adenocarcinoma with biopsy.

**Conclusion:**
Incidental findings of pancreatic pathologies in preoperative MRCP for cholecystectomy are common. While the majority of these findings are benign and don't require further workup, IPMN and suspicious cysts often require additional imaging surveillance, in accordance with guidelines and the age of patients. Detection of suspicious lesions requiring additional diagnostic workup and treatment is rare.

### How to involve patients for conception and design of a multicenter randomized controlled trial using the example of a trial on multimodal prehabilitation for colorectal cancer patients

ID: 540

**Kategorie: SCF - Clinical Studies**

Magdalena Holze[1], Rosa Klotz[1], Solveig Tenckhoff[2], Maximilian Joos[1], Eva Kalkum[2], Das Patient Advisory Board des Studienzentrums der Deutschen Gesellschaft für Chirurgie[2]

[1]*Universitätsklinikum Heidelberg, Klinik für Allgemein-, Viszeral- und Transplantationschirurgie, Heidelberg, Deutschland*, [2]*Universitätsklinikum Heidelberg, Studienzentrum der Deutschen Gesellschaft für Chirurgie, Heidelberg, Deutschland*

**Background:**

Colorectal cancer (CRC) is the third most common cancer entity worldwide and a leading cause of cancer-related mortality. Surgical resection is the primary curative treatment, but places significant physical and psychological strain on CRC patients, often exacerbating pre-existing reduced conditions. Prehabilitation within the context of preoperative interventions as physical exercise, nutritional optimization, and psychological support aims to enhance patients’ resilience before surgery, leading to a reduction in postoperative complications and acceleration of recovery outcomes. Despite its promise, evidence from existing randomized controlled trials (RCTs) remains inconclusive due to small sample sizes, single-center designs, and a lack of standardized interventions.

Patient involvement increases the clinical relevance of research by integrating all stakeholders in the subsequent project stages. As an intervention like prehabilitation can only be successfully implemented with good patient compliance, the integration of different patient target groups can significantly contribute to feasibility and clinical relevance of the planned trial. This conceptional phase with active patient involvement aimed to develop a multicenter RCT evaluating the effect of a personalized multimodal prehabilitation program, including structured peer counseling on CRC patients.

**Materials and methods:**

First, the relevant target groups were identified and a common role definition and responsibilities of the members of the trial team were set. Second, suitable methods for patient involvement for this project were identified. The design of the conceptual phase included multiple methods of stakeholder engagement to ensure comprehensive input. These activities were structured to gather input on trial design, intervention components, and patient-reported outcome measures. To include a broad spectrum on the patient perspective, surveys were disseminated to CRC patients and caregivers to identify the most relevant intervention components, outcomes and potential barriers. Expert workshops were conducted to inquire detailed information on specific aspects regarding the patient population, trial intervention and primary outcomes. Potential barriers to patient participation were identified in focus groups and strategies developed to overcome these obstacles.

**Results:**

In this conceptual phase, the following key stakeholders were identified and involved: the Patient Advisory Board (PAB) of the Study Center of the German Society of Surgery (SDGC), patient support groups (Deutsche ILCO e.V.), CRC patients and different experts such as nutritionists, physiotherapists and psychosomatics as also healthcare providers and prehabilitation experts. First, a comprehensive survey was conducted among 98 patients to identify the perceived importance of various intervention components, outcomes and trial settings. The survey revealed strong support for prehabilitation program components such as patient blood management (4.40), nutritional counseling (4.18), psychosomatic support (4.16) and physical conditioning (4.14), ranked highly important on a Likert scale of 1 to 5, where 5 indicates "very important ". Participants expressed a preference for settings that offer flexibility and accessibility, with supervised ambulatory (3.79) programs. The most relevant key outcomes were quality of (4.57) and time to recovery (4.41), and improvements in postoperative quality of life (4.39). Concerns were expressed regarding the use of an app-based program as well as for high logistical and time efforts.

The results of this survey as well as of several PAB-meetings were addressed in an interdisciplinary expert meeting with the aim of developing the most feasible and effective study design. It was decided to select a patient population with increased perioperative risk, composed of the factors age, frailty and nutrition status, as this patient cohort will benefit most from prehabilitation. Quality of Recovery was chosen as the primary endpoint. Based on the defined trial concept, the identified barriers from the survey were discussed in a PAB focus group and solution strategies were developed.

**Conclusion:**
This conceptual phase with active patient involvement developed a comprehensive and patient-centered study design for a multicenter RCT investigating the effect of a personalized multimodal prehabilitation program in CRC patients undergoing curative surgery. We demonstrated that patient involvement is not only feasible but effective in designing a clinical study. By engaging patients and support groups early and throughout the process, clear benefits could be shown from these insights that ensured the planned trial to be patient-centered, practical but also aligns with the needs and preferences of CRC patients. It could be shown that integrating patient feedback can address real-world barriers, enhance intervention design, and improve trial feasibility. The active contribution of patient representatives led to a more comprehensive understanding of patient needs, highlighting the value of structured peer support. This study underscores the critical role of patient involvement in the conceptual phase of our trial. It sets a foundation for the subsequent conduction phase with the aim to provide high-quality evidence regarding the effect of prehabilitation on CRC care.

**Preoperative MRCP is not Associated With Lower Complication Rates in Laparoscopic Cholecystectomy**
ID: 553

**Kategorie: SCF - Clinical Studies**

Jasmin Zeindler[1 2], Gabriel Fridolin Hess[2], Simone Muenst[3], Martin Bolli[2 4], Otto Kollmar[1 2], Savas Deniz Soysal[5]

[1]*Klinik Chirurgie & Viszeralchirurgie, Kantonsspital Baselland, Liestal, Schweiz*, [2]*Clarunis Universitäres Bauchzentrum Basel Viszeralchirurgie, Basel, Schweiz*, [3]*Universitätspital Basel Pathologie, Basel, Schweiz*, [4]*Allgemein- & Viszeralchirurgie - Luzerner Kantonsspital, Luzern 16, Schweiz*, [5]*Medizinische Fakultät* | *Universität Basel, Basel, Schweiz*

**Background:**
Laparoscopic cholecystectomy is a very common procedure and while preoperative magnetic resonance cholangiopancreatography (pMRCP) is widely used for detection of choledocholithiasis and preoperative planning, the benefits in patients with normal preoperative cholestatic parameters remain unclear. We therefore aimed to investigate a large cohort of patients after laparoscopic cholecystectomy to analyse the impact of pMRCP on complication rates and analysed postoperative histopathological diagnosis according to pMRCP findings.

**Materials and methods:**
This retrospective cohort study consecutively includes all patients receiving elective or acute laparoscopic cholecystectomy between 2010 and 2020. Exclusion criteria were conversion to open cholecystectomy and preoperatively elevated cholestatic parameters. Complication rates were recorded and patients stratified depending on the use of pMRCP for preoperative planning. Postoperative histopathological diagnosis of the surgical specimens was recorded and correlated with MRCP findings.

**Results:**
3163 patients underwent laparoscopic cholecystectomy between 2010 and 2020, 2568 of which showed normal cholestatic parameters preoperatively. The most frequent indications for laparoscopic cholecystectomy were cholecystolithiasis in 72% (1845/2568) of cases, followed by cholecystitis in 18% (465/2568). The majority of patients (2267/2568, 88%) indeed received pMRCP. Postoperative complication rates did not significantly differ between the two groups (396/2267 (17%) in the pMRCP group and 58/301 (19%) without pMRCP, p=0.4691). One case of postoperative pancreatitis occurred in the pMRCP group, versus two reported cases in the other group. Interestingly, the length of hospital stay after cholecystectomy was significantly shorter in the group with pMRCP versus without (4 days (range 1-61 days) versus 7 days (range 1 – 66 days), respectively, p = 0.023). 752/1845 (40%) showed chronic inflammation of the gallbladder mucosa in the postoperative histology despite a lack of preoperative signs of cholecystitis (blood parameters, ultrasound and MRCP findings). Dysplasia or neoplasia was detected in 44/1845 (2.4%) patients. Interestingly, the majority of these 44 patients (n=30, 68.2%) presented with chronic inflammation of the gallbladder on histopathological examination.

**Conclusion:**
Preoperative MRCP seems not to prevent complications in laparoscopic cholecystectomy, since postoperative complication rates were comparable between the two patient groups with and without pMRCP. However, postoperative hospital stay was significantly shorter in patients undergoing pMRCP compared to patients without pMRCP. pMRCP is not accurate in diagnosing chronic cholezystitis. Cholecystolithiasis is associated with chronic inflammation of the gallbladder mucosa in a high proportion of patients. A majority of patients with incidentally diagnosed dysplasia or neoplasia showed chronic inflammation of the gallbladder associated with cholecystolithiasis.

**Complications and long-term survival after Ivor-Lewis esophagectomy**
ID: 588

**Kategorie: SCF - Clinical Studies**

Aram Pascal Abu Hejleh[1], Julian Lemties[1], Naita Wirsik[1], Jin-On Jung[1], Thomas Schmidt[1], Hans Fuchs[1], Wolfgang Schröder[1], Christiane J. Bruns[1], Lars Schiffmann[1]

[1]*Department of General, Visceral, Thoracic and Transplantation Surgery, Faculty of Medicine and University Hospital Cologne, University of Cologne, Cologne, Germany, Köln, Deutschland*

**Background:**
Though the field has constantly improved operative technique and perioperative management over the last decades, esophagectomy (OE) remains a complex procedure with a considerable risk of severe postoperative complications. It has long been considered that major postoperative complications particularly anastomotic leakage (AL) not only harm patients' short-term outcome and quality of life but also have detrimental effects on oncological long-term survival. This retrospective study was designed to retest the hypothesis that complications after OE impact on long-term survival in a large and highly standardized cohort of esophageal cancer patients.

**Materials and methods:**
733 patients who underwent Ivor-Lewis esophagectomy (IL-OE) for cancer from 2016-2023 at our tertiary center were analyzed from a prospectively maintained database. Postoperative complications were correlated to overall survival.

**Results:**
Neither occurrence of major complications > Clavien-Dindo (CD) IIIA (Median OS not reached in both groups, p =0.46, HR 0.877) nor AL (Median OS not reached for no AL vs. 50 month for AL, p =0.48, HR 0.879) nor pulmonary complications (Median OS not reached in both groups, p =0.59, HR 1.12) had an impact on overall survival. However, the necessity for postoperative readmission to ICU (reaICU) had a significant impact on overall survival (Median OS not reached for no reaICU vs. 40 month for reaICU, p =0.035, HR 0.6844). Mean follow up was 27 months.

**Conclusion:**
Postoperative complications and in particular anastomotic leakage after IL-OE may play a less significant role in harming patients' overall survival than previously anticipated. This might be due to a highly sufficient management of complications that progressively avoids requirement of intensive care treatment.

Abstracts – DCK 2025 – 142nd Congress of the German Society of Surgery – Munich, March 26–28 • DOI 10.1515/iss-2025-2005
Innov Surg Sci 2025; 10 (Special Suppl 1): s256–s367

### Intestinal alkaline phospatase and bacterial lipopolysaccharides as predictors of short- and long-term outcome after oncologic surgery

ID: 592

**Kategorie: SCF - Clinical Studies**

Clara Maraß[1], Viktor von Ehrlich-Treuenstätt[1], Michael Ingrisch[2], Ulrich Wirth[1], Jens Werner[1], Wolfgang Kunz[2], Florian Kühn[1]

[1]*LMU München, Klinik für Allgemein-, Viszeral- und Transplantationschirurgie, München, Deutschland*, [2]*LMU München, Klinik und Poliklinik für Radiologie, München, Deutschland*

**Background:**
The gut brush boarder enzyme Intestinal alkaline phosphatase (IAP) is mainly produced by enterocytes in the duodenum and plays a pivotal role in regulating the gut barrier function aas well as maintaining intestinal homeostasis. By detoxifying bacterial lipopolysaccharides (LPS) it helps preventing systemic inflammation and endotoxemia. IAP-deficiency has recently been linked to various pathologies such as diabetes, metabolic syndrome and frailty. Sarcopenia, a major component of frailty syndrome, is associated with a worse outcome in patients undergoing abdominal surgery. To assess body composition, new CT evaluation tools (Body Composition Analysis) based on A.I. show increasing accuracy in identifying patients with sarcopenia.This study aimed to investigate the role of IAP and LPS as predictors of short- and long-term outcome after oncologic surgery and as potential biomarkers for frailty.

**Materials and methods:**
We conducted a prospective cohort study to investigate the association between IAP, LPS, sarcopenia, surgical outcomes and overall survival in patients undergoing abdominal oncologic surgery. In total, 92 patients were included. Pre- and postoperative IAP and LPS levels were measured in stool and blood samples and we collected various clinical parameters for multivariate analysis. In a subgroup of 54 patients, the diagnosis of sarcopenia was based on the measurement of lumbar skeletal muscle mass at level of L3 by a CT-based A.I. Body Composition Analysis.

**Results:**
Our results showed that preoperative IAP levels were significantly lower in patients with sarcopenia ($p=0.0323$) and old age ($p=0.0205$), with preoperative LPS showing a tendency to be higher in sarcopenic patients. Patients with low preoperative IAP activity had significantly higher postoperative LPS values ($p=0.0196$) with higher morbidity in surgical outcomes (Clavien-Dindo III-IV; Organ space infection) and an elongated hospitalization than those with regular preoperative IAP activity.
Concerning the long-term outcome after oncologic surgery, patients with a progression of disease in general, including local recurrence and metastasis, had significantly lower pre- ($p=0.046$) and postoperative levels of IAP ($p=0.017$) and concurrent higher pre- ($p=0.017$) and postoperative levels of LPS ($p=0.024$). In addition patients with low preoperative IAP activity had lower overall survival ($p=0.0010$). Patients who died as a direct result of their oncologic primary disease during the follow-up had significantly higher pre- ($p=0.024$) and postoperative levels of LPS ($p=0.009$).

**Conclusion:**
In conclusion, our study highlights a possible role of IAP and LPS as potential biomarkers for predicting sarcopenia and short- and long-term postoperative outcomes in patients undergoing oncologic surgery.

### Total versus Partial Pancreatectomy in Patients with Pancreatic Cancer Arising from Multifocal or Diffuse Intraductal Papillary Mucinous Neoplasia

ID: 599

**Kategorie: SCF - Clinical Studies**

Ingmar F Rompen[1], Joseph Habib[2], Benedict Kinny-Köster[1], Brady Campbell[3], Thomas Stoop[4], Christoph Kümmerli[5], Paul Andel[6], Charlotte Leseman[4], Carolin Lesch[2], Lois Daamen[6], Ammar Javed[2], Kelly Lafaro[3], Henrik Nienhüser[1], Adrian Billeter[5], Quintus Molenaar[6], Beat Müller[5], Marc Besselink[4], Jin He[3], Martin Loos[1], Markus W. Büchler[7], Christopher Wolfgang[2]

[1]*Klinik für Allgemein, Viszeral und Transplantationschirurgie, Heidelberg, Deutschland*, [2]*NYU Grossman School of Medicine, New York City, Vereinigte Staaten*, [3]*Department of Surgery, Johns Hopkins Hospital, Baltimore, Vereinigte Staaten*, [4]*Department of Surgery, AmsterdamUMC, Amsterdam, Niederlande*, [5]*Department of Surgery, Clarunis, University of Basel, Basel, Schweiz*, [6]*Regional Academic Cancer Center Utrecht, Utrecht, Niederlande*, [7]*Department of Pancreatic Surgery, Champalimaud Foundation, Lisbon, Portugal*

**Background:**
Major risk factors for invasive progression in the remnant gland include multifocality, diffuse main duct dilation, and the presence of invasive cancer. In these high-risk patients, a total pancreatectomy (TP) may be oncologically beneficial. However, current guidelines discourage TP, especially in elderly patients. Therefore, we aimed to investigate the impact of TP on oncological outcomes for patients at high-risk of local recurrence or secondary progression in the remnant gland after partial pancreatectomy (PP) for IPMN-associated cancer.

**Materials and methods:**
This international multicenter study compares TP versus PP in patients with adenocarcinoma arising from multifocal or diffuse IPMN (2002-2022). Log-rank test and multivariable Cox-analysis with interaction analysis was performed to assess overall survival (OS), disease-free survival (DFS), and local-DFS.

**Results:**
Of 359 included patients, 162 (45%) were treated with TP, whereas 197 (55%) underwent PP. Despite TP and PP having similar R0-rates (59% vs. 58%, p=0.866), patients undergoing a TP had significantly longer local-DFS compared to PP (p=0.039). However, no difference in OS was observed between the two surgical approaches (p=0.487). In a multivariable analysis, young age (optimal cut-off ≤63.6 years) was associated with an OS benefit derived from TP (HR:0.44, 95%CI:0.22-0.89), whereas no significant difference was observed in elderly patients (HR:1.24, 95%CI:0.92-1.67, $P_{interaction}$=0.007).

**Conclusion:**
Since overall, patients with diffuse or multifocal IPMN with an invasive component do not benefit from TP in terms of OS, the indication for TP may be individualized to patients who have sufficient life expectancy to benefit from the prevention of secondary progression or local recurrence. Accordingly, a TP may be the preferred surgical approach for young patients, whereas in older patients, the indication for a TP instead of a PP should be considered more carefully.

### Pancreatic ductal adenocarcinoma in the Young- an Analysis of Patients under 50 Years of Age within the German Cancer Registry

ID: 643

**Kategorie: SCF - Clinical Studies**

Katja Janke[1], Lennart von Fritsch[1], Louisa Bolm[1], Rüdiger Braun[1], Steffen Deichmann[1], Jannis Duhn[1], Thaer Abdalla[1], Tobias Keck[1], Ulrich Wellner[1], Kim Honselmann[1]

[1]*University Medical Center Schleswig-Holstein, Campus Luebeck, Department of Surgery, Luebeck, Deutschland*

**Background:**
Pancreatic ductal adenocarcinoma (PDAC) is a devastating disease with a 5-year overall survival of less than 13% and is predicted to be the second leading cause of cancer death by 2030. The median age of an average pancreatic cancer patient is around 70 years, however there is the notion of an increasing incidence in the younger population. In this study, we aimed to characterize the pancreatic cancer population younger than 50 years.

**Materials and methods:**
Retrospective data was obtained from pooled data of 18 clinical cancer registries in Germany collected by the German Cancer Registry Group of the Association of German Tumor Centers. Patients with ICD-10-code C25.0-3, and C25.7-9, and histo code: 8140 und 8500 (WHO classification) were included. Data was dichotomized into patients under and equal 50 years and older than 50 years.
Overall and disease-free survival (OS/DFS) were analyzed using Kaplan-Meier analyses. Subgroups by histologic pancreatic cancer, type of surgery and years of first diagnosis were analyzed. Cox regression for OS were performed.

**Results:**
Almost five percent (4.5 % (3.639/81.538) of PDAC patients treated in Germany between 1998 and 2023 were younger than 50 years at the time of diagnosis. When we analyzed PDAC diagnoses between 1998-2007, 2008-2015 and 2016-2023, the number of PDAC diagnoses rose from 14.110 to 42.055 patients per eight- year period, but percentage of young patients decreased over time (7% vs. 5 % vs. 3%, respectively). Young patients had significantly higher tumor stage, when compared to older PDAC patients (for example cT4: 42,2% vs. 37,71%, $p<0.001$), and were subjected to any type of treatment more often (70% (n=2575) and 60% (n=46606), $p<0.001$). Median OS, but not DFS was significantly better in young patients (12 months vs. 8 months, $p < 0.001$ and 9.6 vs. 9.1 months, $p=0.099$). When treated with surgery with or without neoadjuvant chemotherapy, OS was also significantly longer in young patients (Median 19 (18-20) vs 16 (15-16) months, $p<0.001$). COX regression analysis revealed younger age among other known factors as independent predictor for overall survival.

**Conclusion:**
PDAC is a disease with rising incidence, however diagnoses in young patients are decreasing over time. Although these young patients present with higher tumor stage, overall survival, but not disease-free survival was significantly better in young PDAC patients. The data suggests that an aggressive treatment approach in young PDAC patients may lead to improved overall survival.

### Metabolic comorbidities in Early-onset Colorectal Cancer: an Observational Cohort study

ID: 664

**Kategorie: SCF - Clinical Studies**

Jacques Hilbert[1], Fernando Topfstedt[1], Hannes P. Neeff[1], Stefan Fichtner-Feigl[1], Gabriel Seifert[1]

[1]*Universitätsklinikum Freiburg, Klinik für Allgemein- und Viszeralchirurgie, Freiburg im Breisgau*

**Background:**

Colorectal cancer (CRC) is the third most common cancer and the second leading cause of cancer-related deaths. CRC is classified into early-onset (eoCRC) and late-onset (loCRC) based on diagnosis age, typically 50 years. While loCRC rates are declining, eoCRC cases are rising in countries like the US, China, Japan, and several European nations. The causes are unclear but likely involve genetic, environmental, and lifestyle factors. Patients with eoCRC often have more advanced, poorly differentiated tumors and are underdiagnosed due to screening programs targeting loCRC. About one-third of eoCRC cases have hereditary links, while the rest are sporadic. Quantifying the association of metabolic risk factors for eoCRC endpoints is extremely challenging and requires high-quality data. The prevalence of metabolic comorbidities, such as type 2 diabetes (T2D) and obesity, is significantly higher in patients with eoCRC compared to patients with loCRC.
This retrospective cohort study aims to describe the prevalence of metabolic risk factors in patients with colorectal carcinoma and seeks to establish and compare metabolic predictors for cancer-free survival in eoCRC and loCRC.

**Materials and methods:**

We established a retrospective cohort using all the patients that underwent surgery on a primary colorectal malignoma at the University Hospital of Freiburg since 2012. This includes a total of 251 cases of eoCRC and 1,353 cases of loCRC. We used a combination of data from Patient Management System, pathology reports, anesthesiology reports, and CCCF- and FREEZE-biobank. The data provided includes (but is not limited to) size and weight, medication before surgery, medical diagnoses, laboratory parameters and date of last follow-up.

Patients were excluded for this analysis if they lacked reliable data for metabolic comorbidities such as Type 2 diabetes (T2D), hyperlipidemia (HLD), arterial hypertension (HTN), obesity and metabolic syndrome (MetS).

**Results:**

In this retrospective cohort study, we aim to provide descriptive data and severity of metabolic risk factors known to associate with earlier incidence in 1,604 CRC-patients. We provide an in-depth description of risk factor distribution, comparing eoCRC with loCRC. Furthermore, we investigate whether these factors independently associate with overall-, disease-specific, and metastasis-free survival.
As this research is still ongoing, we are to this date unable to provide exact data.
A statistical analysis will follow in the coming months.

**Conclusion:**

Large epidemiologic studies have demonstrated an independent association of metabolic risk factors and CRC incidence as well as early incidence. However, these registry studies often lack data granularity on risk factors involved. Here, we provide an in-depth cohort study by leveraging an integrative multi-system database. However, as our research is still ongoing, we are unable to provide any findings at this point.

### Assessing revascularization success in patients with Peripheral Artery Disease using Multispectral Optoacoustic Tomography

ID: 670

**Kategorie: SCF - Clinical Studies**

Briain Haney[1], Yi Li[1], Julius Kempf[1], Werner Lang[1], Adrian Regensburger[2], Emmanuel Nedoschill[2], Axel Schmid[3], Michael Uder[3], Ferdinand Knieling[2], Ulrich Rother[1]

[1]*Universitätsklinikum Erlangen, Gefäßchirurgie, Erlangen/Bavaria, Germany, Deutschland*,
[2]*Universitätsklinikum Erlangen, Pädiatrie, Erlangen/Bavaria, Germany, Deutschland*,
[3]*Universitätsklinikum Erlangen, Radiologie, Erlangen/Bavaria, Germany, Deutschland*

**Background:**

Peripheral artery disease (PAD) is a significant cause of lower extremity morbidity, manifesting as claudication or chronic limb-threatening ischemia (CLTI). Multispectral optoacoustic tomography (MSOT) is a novel imaging technique which provides quantitative imaging of tissue perfusion and oxygenation. This study evaluates the perfusion changes post-revascularization using MSOT in PAD patients, comparing outcomes across different revascularization strategies.

**Materials and methods:**

In this prospective study, 40 patients with PAD (Claudication or CLTI) were enrolled and assigned to one of four groups: Claudicants undergoing open surgical therapy (n=10), claudicants undergoing endovascular therapy (n=10), CLTI undergoing open surgical therapy (n=10), and CLTI undergoing endovascular therapy (n=10). MSOT measurements of perfusion parameters, including deoxygenated hemoglobin (Hb), oxygenated hemoglobin (HbO2), and oxygenation (mSO2), were performed before and after revascularization. The impact of revascularization on microcirculation was assessed by comparing pre- and post-intervention MSOT-values.

**Results:**

All patients underwent successful revascularization. MSOT analysis revealed significant improvements in perfusion parameters post-revascularization across all groups. The differences in ΔmSO2 (the change in oxygenation post-exercise) were particularly notable, indicating enhanced microcirculation. Patients in the open surgical therapy groups demonstrated more pronounced improvements compared to those undergoing endovascular therapy, irrespective of PAD stage. In claudicants, the mean ΔmSO2 increased from -0,1755 to -0,014 ($p<0.05$) post-surgery and from -0,109 to -0,002 ($p<0.05$) post-endovascular therapy. For CLTI patients, the mean ΔmSO2 increased from -0,140 to -0,006 ($p<0.05$) post-surgery and from -0,127 to -0,011 ($p<0.05$) post-endovascular therapy.

**Conclusion:**

MSOT has the potential to be a valuable tool for assessing microcirculatory changes post-revascularization in PAD patients. Both surgical and endovascular therapies significantly improve perfusion, with open surgical interventions showing greater efficacy. These findings suggest MSOT could be integral in guiding and evaluating future revascularization strategies in PAD patients.

**A randomized placebo-controlled trial to examine the effect of acupuncture strategies on pain relief and gastrointestinal recovery after bariatric surgery - the RAPRAB trial**
ID: 801

**Kategorie: SCF - Clinical Studies**

Erfan Ghanad[1], Niki Taebi[1], Xiaoyan Huang[1], Christoph Reißfelder[1], Mirko Otto[1], Cui Yang[1]

[1]*Universitätsmedizin Mannheim, Chirurgische Klinik, Mannheim, Deutschland*

**Background:**
Peritoneal irritation and bowel manipulation following abdominal surgeries often lead to postoperative pain and vegetative symptoms such as nausea, vomiting, and gut motility disorders. Acupuncture has shown effectiveness in managing postoperative pain and associated vegetative syndromes. A pilot study established a concept of check-point acupuncture based on the sensitivity of six visceral indicator points (G1-G6), showing significant pain reduction and increased pain threshold in bariatric surgery patients.

**Materials and methods:**
This randomized controlled trial employs a triple-blinded study design. Patients undergoing bariatric surgery are randomized to receive either verum or sham acupuncture. The study protocol involves: An experienced acupuncturist marking verum and sham acupoints; Baseline assessments including Visual Analog Scale (VAS) for pain, abdominal skin temperature, pain threshold via algometry, heart rate variability, and spirometry; A novice acupuncturist applying needles to randomly selected points.; Follow-up assessments at 5 minutes, 1 hour, and 24 hours post-acupuncture. The primary endpoint assesses postoperative reduction evaluated via VAS. Secondary endpoints include use of morphine equivalents, pain thresholds using algometry, timing of first bowel movement, duration of hospital stay, tidal volume, quality of recovery (QoR-40), mobility measures, oral liquid consumption, and 30-day postoperative outcomes.

**Results:**
The study aims to demonstrate the efficacy of check-point acupuncture in reducing postoperative pain and improving gastrointestinal recovery after bariatric surgery. The triple-blinded design will help minimize bias and provide robust evidence for the effectiveness of this acupuncture strategy.

**Conclusion:**
The RAPRAB trial will provide valuable insights into the potential of check-point acupuncture as an adjunct therapy for post-bariatric surgery care. If successful, this approach could offer a non-pharmacological option for improving pain management and accelerating gastrointestinal recovery in bariatric surgery patients, potentially reducing opioid use and associated complications.

**Interventional Multicenter randomised-controlled trial on the influence of a PERsonalized student-led digital multimodal prehabilitation Intervention in patients undergoing intracavity Oncological surgery – IMPERIO. A study protocol**
ID: 913

**Kategorie: SCF - Clinical Studies**

Chir-Net Sigma Study Group[1]

[1]*Koordinierungszentrale am Studienzentrum der Deutschen Gesellschaft für Chirurgie (SDGC), Universitätsklinikum Heidelberg, Heidelberg, Deutschland*

**Background:**
Whilst it is common knowledge that physical performance, nutritional status and sufficient mental health are significant factors on subjective (Quality of Life) and objective (perioperative morbidity and mortality) outcomes after cancer surgery, current clinical practice and research mainly focuses on the rehabilitative aspects. However, similar to an athletic competition, preparation prior to surgery is critical for patients. Prehabilitation offers the potential to improve the quality of recovery of patients by increasing their functional reserves and thus reducing the risk of postoperative complications. Besides physical training, prehabilitation consists of optimized nutrition and psychological supervision.
It has been shown that physical exercise in a preoperative setting is feasible, and preoperatively improved nutrition or exercised training favors physical performance and shortens the length of stay. The positive impact of physical exercise is not only limited to objective factors, as a more positive subjective assessment of one's quality of life could be achieved. The effectiveness of prehabilitation has already been shown in other surgical specialties, e.g., cardiothoracic or orthopedic surgery. Whether prehabilitation can reduce morbidity or lethality is still controversial.
As studies investigating prehabilitative concepts in visceral oncology surgery are limited to specific organs, evidence on broad surgical oncology is sparse and high quality trials are urgently needed. It is apparent that preoperative training and support for patients has been very costly to date and is not accessible to a broad mass of patients, as this would involve excessive human resources. To implement prehabilitation as standard operating procedures in a majority of hospitals in the future, cost- and labor effectiveness have to be held in consideration when developing and testing said multimodal programs. The examinations to determine an improved physical condition have so far been very laborious. For this purpose, we are currently planning a prospective, multicenter randomized-controlled outcome assessor-blinded superiority trial with two parallel study groups using a digitally supported low concept prior to major oncological surgery.

**Materials and methods:**
The objective of this study is to determine whether a student-led digital multimodal prehabilitative program, consisting of physical exercise, nutrition, and cognitive behavioral therapy interventions, improves postoperative recovery compared to the standard of care in patients undergoing elective intracavity cancer surgery. The study, named IMPERIO, is planned as a multicenter randomized-controlled trial with outcome assessor blinding, featuring two parallel study groups.
Both patients in the interventional group and those in the control group will undergo a standardized student-led screening and assessment at least 10 weeks prior to their scheduled cancer resection. Based on this assessment, patients will be classified as “low-risk” or “high-risk” with respect to nutrition and fitness levels. The interventional group will receive a digital exercise program via the SurgiFit App, which will be adapted in intensity according to their risk group. Additionally, standardized feedback calls from medical students will be included as part of the intervention. Patients in the intervention group will also receive a DGEM nutritional advice brochure for cancer patients (LEKuP), along with supplementary nutritional guidance for those classified as high-risk. Furthermore, all patients in the intervention group will have access to the ACTonCancer Program via the eSano platform.

Patients in the control group will receive perioperative treatment according to the local standard of care. All other preoperative and postoperative care will follow local standards and will be consistent for both groups.
The primary endpoint will be the quality of recovery, measured via the QoR-40 at baseline and on postoperative day 4 ± 1 (or at discharge, whichever comes first). Secondary endpoints will include the “Sit and Stand Test” at baseline, before surgery, and at discharge; handgrip strength at baseline, before surgery, and at discharge; health-related quality of life measured via the EORTC QLQ-C30 at baseline, before surgery, on postoperative day 8 (or at discharge, whichever comes first), and on postoperative day 30. Morbidity and mortality will be assessed using the modified comprehensive complication index within the first 30 postoperative days, and frailty will be evaluated using the FRAILTY score at baseline, before surgery, and at discharge (activities of daily living).

**Results:**
We will start with the first patient in summer 2025 and plan with a study duration of about 24 months.

**Conclusion:**
Previous SIGMA trials showed that studies, even randomized controlled multicentric trials, planned, conducted and analyzed by medical students can be successfully published. IMPERIO will make an important contribution to ERAS and clarify pioneering questions.

**TransValid-A study (ClinTrials.gov: NCT03034473) for real world multimodal treatment (MMT) of patients with rectal cancer: Do we really have an early predictor for long-term DFS?**
ID: 941

**Kategorie: SCF - Clinical Studies**

Johannes Martens[1], Andreas Leha[2], Timotheus Ritter-Vanfloorop[1 3], Julia Kitz[4], Johanna Kreutzer[1], Andrea Hille[5], Julie Schanz[6 7], Torsten Liersch[1]

[1]*University Medical Center of Göttingen, Department of General, Visceral and Pediatric Surgery, Göttingen, Deutschland*, [2]*University Medical Center of Göttingen, Scientific Core Facility for Medical Biometry and Statistical Bioinformatics, Göttingen, Deutschland*, [3]*Cellitinnen Hospital St, Antonius Köln (Bayenthal), Department of Trauma, Hand and Orthopaedic Surgery, Köln, Deutschland*, [4]*University Medical Center of Göttingen, Department of Pathology, Göttingen, Deutschland*, [5]*University Medical Center of Göttingen, Department of Radiotherapy and Radiation Oncology, Göttingen, Deutschland*, [6]*University Medical Center of Göttingen, Department of Haematology and Medical Oncology, Göttingen, Deutschland*, [7]*University Medical Center of Göttingen, Department of Clinical Chemistry, Göttingen, Deutschland*

**Background:**
The prospective single-arm multicenter TransValid-A study (ClinTrials.gov: NCT03034473; funded by the German Research Foundation; DFG-GZ: LI 1727/3-1) assessed the efficacy of 5-FU-based preoperative chemoradiotherapy (preopCRT), quality-controlled total mesorectal excision (qTME) and adjuvant chemotherapy (adCTx; FOLFOX or 5-FU) in patients with locally advanced rectal cancer (LARC, stages cUICC ≥ II; < 12 cm above the anal verge). We aimed to find a predictor for disease-free survival (DFS) by analyzing various clinico-pathological risk factors using a multivariate COX proportional hazards regression model.

**Materials and methods:**
Between August 2011 and January 2015, 194 patients with a LARC (stages II / ≥ III: 14.4% / 85.1%) localized in the lower (41.8%) or mid (57.7%) rectum (f: 36.1%; m: 63.9%; median age: 64 y; ECOG 0: 63.9%; I: 28.4% and II: 7.7%) were enrolled. Multimodal treatment (MMT) was implemented across 19 research locations in Germany, with biomaterials being preserved before, during, and after surgery, as well as during follow-up appointments spanning over five years. For data recording electronic case report forms (eCRF) and secuTrial® database were used. Those four risk factors out of diverse parameters [e.g. age, sex, TNM-/UICC stage, BMI, CRM status (circumferential resection margin), study site and the mNAR (modified neoadjuvant rectal score)] with strongest association to DFS in uni-variable models (by significance from likelihood-ratio-tests) were selected for inclusion in multi-variable models. From clusters of strongly correlated factors (correlation ≥ 0.65) only the representative with the strongest association was included to avoid multi-collinearity problems. The significance level was set to alpha = 5% for all statistical tests. All analyses were performed with the statistic software R (version 4.4.0; R Core Team 2024).

**Results:**
180 LARC patients had started the preopCRT according to the protocol with RT + 5-FU (92.8%), with RT + 5-FU + OX (3.3%) and RT + capecitabine (CAP, 3.9%), respectively. Subsequently, 174 patients underwent qTME-surgery with optimal, moderate and poor quality in 84.5%, 7.5% and 1.7%, respectively. Major complications (CTC-AE G 3 and G 4) occurred in 24.1%; the 30-day mortality was 1.2%. The 73.6% rate of stages ≤ II (vs. ≥ III in 26.4%) showed a remarkable LARC response to preopCRT. A complete remission (CR; ypT0) was achieved in 20.1% and the rate of stages 0 / I was 19.5% and 26.4%, respectively. The 3- and 5-year DFS probability rates were 72.6% and 68.4%, respectively (50 events), with a restricted mean DFS of 90.3 ± 5.8 months.
The multivariable Cox regression model for DFS using the risk factors ypUICC status, ratio of lymphnode metastases (LNM) / LN, age and circumferential resection margin (CRM) status, revealed only ypUICC

stages ≤ I to be significantly predictive for an excellent DFS versus patients with stages ≥ II (HR: 2.755; 95%-CI [1.302; 5.8]; p=0.008).

**Conclusion:**
Multivariable COX regression models for DFS revealed that patients with stage 0 / I after 5-FU-based CRT had an outstanding prognosis compared to those with residual stages of ≥ II. Therefore, the postsurgical ypUICC stage is a valuable indicator for determining the most suitable CTx.

**Combined IL6R inhibition and immunotherapy as a novel neoadjuvant therapeutic approach in murine pancreatic cancer: a preclinical study protocol**
ID: 368

**Kategorie: SCF - Immune Therapy**

Ibrahim Halil Gurcinar[1], Okan Safak[1], Sergey Tokalov[1], Helmut Friess[1], Güralp Ceyhan[2], Rouzanna Istvanffy[1], Ihsan Ekin Demir[1]

[1]*Technische Universität München , Klinikum rechts der Isar, Chirurgie, München*, [2]*Mehmet Ali Aydinlar Acibadem University, Chirurgie, Istanbul, Turkey*

**Background:**
Neoadjuvant therapy (neoTx) plays a critical role in enhancing the success of surgical interventions in pancreatic cancer, particularly in borderline resectable (BRPC) and locally advanced (LAPC) cases. Immunotherapy has gained increasing significance in the treatment of pancreatic ductal adenocarcinoma (PDAC), especially when combined with agents targeting immune inhibitory pathways. Our previous studies have demonstrated that neoadjuvant Gemcitabine therapy in a murine R0-resectable PDAC model reduces tumor size and volume, improves overall survival rates, and decreases metastatic potential. Additionally, we have shown that resistance to Pembrolizumab (Anti-PD-1) can be overcome when combined with an interleukin-6 receptor inhibitor (Tocilizumab).

**Materials and methods:**
We hypothesize that combined neoadjuvant treatment (Pembrolizumab, Tocilizumab, and Gemcitabine) could improve resectability in a murine PDAC model. To test this hypothesis, we will generate R0-resectable PDAC in LysM-Cre; IL6fl/fl mice using orthotopic and electroporation techniques. After tumor development, the treatment regimen will be initiated, and the effects on tumor tissue will be assessed using ultrasonography (USG) and pain sensitivity with Von-Frey testing.

**Results:**
Pembrolizumab will be administered for 1-8 weeks, Tocilizumab for 3-6 weeks, and Gemcitabine for 3-6 weeks. During the treatment, tumor size will be monitored via ultrasonography, and pain sensitivity will be measured using the Von-Frey test. The primary outcome measure will be overall survival, while secondary outcomes will include pain and disease-free survival.

**Conclusion:**
Successful neoadjuvant therapy in BRPC and LAPC cases could enhance the chances of surgical intervention and improve overall survival and disease-free survival rates in PDAC patients. This translational research approach may offer a novel and more effective treatment option for PDAC patients, addressing an unmet clinical need.

**Patterns of immune evasion in primary and metastatic colorectal cancer**
ID: 939

**Kategorie: SCF - Immune Therapy**

Elena Hagen[1], Martin Thelen[1], Kerstin Wennhold[1], Maria Garcia-Marquez[1], Jonas Lehmann[1], Miriam Werner[1], Pauline Volkmar[1], Su Ir Lyu[2], Michael von Bergwelt[3], Georg Dieplinger[4], Christiane Bruns[4], Alexander Quaas[2], Hans Schlößer[1 4]

[1]*Center for Molecular Medicine Cologne (CMMC), Faculty of Medicine and University Hospital Cologne, Cologne, Deutschland*, [2]*Institute of Pathology, Faculty of Medicine and University Hospital Cologne, Cologne, Deutschland*, [3]*Department of Internal Medicine III, University Hospital of Ludwig Maximilians University, Munich, Deutschland*, [4]*Department of General, Visceral, Cancer and Transplantation Surgery, Faculty of Medicine and University Hospital Cologne, Cologne, Deutschland*

**Background:**
Colorectal cancer (CRC) is one of the most common cancer entities worldwide and contributes significantly to the overall cancer-related mortality. Stage-dependent therapeutic algorithms of CRC are mainly based on surgery, chemotherapy, and radiation. The NICHE-I and NICHE-II trials demonstrated exceptional efficacy of neoadjuvant immunotherapy targeting the PD-(L)1 pathway in a subgroup of CRC patients with DNA-mismatch-repair deficiency (dMMR)/microsatellite instability (MSI). In contrast, results of large clinical trials evaluating anti-PD-(L)1 immunotherapy in DNA-mismatch-repair-proficient (pMMR)/ microsatellite-stable CRC were disappointing. As the liver is the most important metastatic site of CRC, we aim to elucidate mechanisms of resistance in the microenvironment of hepatic metastasis of CRC patients.

**Materials and methods:**
We included samples from 68 patients in our study, 28 paired primary tumor and hepatic metastases of CRC patients, 15 colorectal carcinomas and 15 hepatocellular carcinomas. Tumor-infiltrating lymphocytes (TILs), PBMCs, healthy tissue and metastases infiltrating lymphocytes were generated from patient samples. We perform flow cytometry to compare immune cell subsets in different samples. Furthermore, immunohistochemical stainings (CD3, CD8, CD20) were performed to calculate the immune score and to quantify TLS in the TME. Expression of genes related to antigen presentation will be analyzed by 3'-RNA-sequencing (out of FFPE-Material). Additionally, we will compare expression of 11 important proteins of the antigen-presenting machinery by immunohistochemistry.

**Results:**
We found several differences of the immunological infiltrate in the metastases compared with the corresponding primary tumor. Analysis of T-cell abundance in primary tumors and the corresponding metastasis revealed a lower T-cell infiltrate in metastasis. Preliminary FACS analyses show a significant decrease of CD4+ T- fractions in metastasis infiltrating lymphocytes (MILs) compared to TILs. This reduction is not due to regulatory T cells. Furthermore, we found no differences in PD1 or PDL1 expression on T cells between TILs and MILs. We will present detailed analyses regarding expression of co-inhibitory molecules on lymphocyte subsets in the different compartments. RNA expression analyses further reveal heterogeneous expression patterns of proteins relevant to antigen presentation. Additionally, several proteins were identified that are significantly more downregulated in metastases compared to primary colorectal carcinoma such as FBXW7 or ERAP2. These findings are also supported by comparing matched samples of metastases and primary tumors. Furthermore, we will present the expression data of relevant tumor-associated antigens and the corresponding protein-level expression through immunohistochemical staining.

**Conclusion:**
First results of our study demonstrate decreased immune cell abundance in the metastatic environment, and we aim to elucidate the underlying mechanisms of immune escape. Especially combination therapies aiming to upregulate antigen presentation within metastases appear promising for combined immunotherapy approaches.

### Preoperative Progressive Pneumoperitoneum and Botulinum Toxin A in a High-Risk Patient With Loss of Domain Inguinoscrotal Hernia

ID: 89

**Kategorie: SCF - Innovative Surgical Techniques**

Baco Stanko[1]

[1]*Prijedor, Prijedor, Bosnia and Herzegovina*

**Background:**
We present a challenging case of a loss of domain (LOD) inguinoscrotal hernia in a 77-year-old high-risk patient, successfully managed with the complementary preoperative use of progressive pneumoperitoneum (PPP) and botulinum toxin A (BTA) without complications. Giant inguinoscrotal and LOD hernias, particularly in multimorbid patients, are highly complex and require meticulous preoperative preparation. In this case, PPP was performed with ambient air and a gradual increase in insufflation volume, while BTA was injected at three points on each side, with a total dose of 300 IU. This approach facilitated a complication-free increase in abdominal cavity volume and the repositioning of chronically eventrated abdominal contents. The technique proved safe, feasible, and effective, contributing to atraumatic adhesiolysis, reduced operative time, and avoidance of more invasive surgical methods.

**Materials and methods:**
A 77-year-old man presented to our ambulance with a giant right-sided inguinoscrotal hernia that he had experienced for over 10 years. Due to the hernia's enormous size, extending almost down to his knees, he suffered from abdominal and groin pain, a buried penis, difficulty with normal daily activities such as walking and dressing, and spontaneous urinary leakage. His high operative risk had led to multiple previous denials of surgery. The patient's comorbidities included hypertension, chronic renal failure, ischemic cardiomyopathy, NYHA II heart failure, severe mitral valve insufficiency, intermittent left bundle branch block, atrioventricular block gradus primus, prostatic hyperplasia, and NSTEMI myocardial infarction three years prior. A preoperative CT scan revealed a 33 cm long hernial sac completely filled with small and large intestines.
Under local anesthesia, we placed a simple Foley catheter into the abdominal cavity through a 20-mm vertical subxiphoid incision to induce pneumoperitoneum. After 10 days and an insufflated volume of 6.7 L, we were not satisfied with the progress of abdominal wall distention, so we proposed BTA to the patient. We injected 300 IU of BTA between the external and internal oblique muscles, 150 IU on each side of the abdominal wall at three points between the costal margin, iliac crest, and lateral to the semilunar line. The total insufflation period was 20 days, with daily volumes ranging from 600 to 1400 cc, amounting to a total of 20.7 L of ambient air. We restored the hernia contents without the need for bowel resection and performed a Shouldice natural tissue repair. The surgery lasted 90 minutes. The patient's recovery proceeded smoothly, and they were discharged on the 10th postoperative day.

**Results:**
The classification of hernias specifies that LOD hernias either pose a significant risk of complications due to increased IAP or require additional reconstructive procedures beyond primary fascial closure and simple content reduction. An alternative definition considers hernias with more than 20% of abdominal contents as straightforward.
Managing LOD hernias should be performed by experienced hernia specialists, especially in multimorbid patients, as multimorbidity significantly impacts functioning and survival, contributing to nearly 70% of postoperative deaths. PPP is a prehabilitation technique that can expand the abdominal cavity, creating space for chronically impacted contents and reducing the risk of compartment syndrome and related complications. PPP alone initially did not yield satisfactory results, and we observed discomfort when the injected air volume exceeded 1,000 cc, suggesting a gradual approach is preferable.

BTA allows for reversible separation of abdominal wall components, reducing the need for invasive surgery and associated complications. We opted not to use mesh implantation or drains, based on the patient's health status, local findings, and the operator's expertise, with very satisfactory results from the Shouldice operation. The combined use of PPP and BTA achieved pneumatic adhesions, dissection of the hernial sac, and a significant reduction in operative time.

**Conclusion:**
Giant inguinoscrotal and LOD hernias, especially in multimorbid patients, require preoperative prehabilitation. In our experience, PPP alone was insufficient for optimal conditions. However, when combined with BTA, we achieved excellent results. This approach is safe, feasible, and effective, facilitating pneumatic, nontraumatic adhesiolysis, reducing surgery time, and minimizing the need for more invasive techniques such as hemicolectomies, component separation, or TAR. The catheter placement procedure is reproducible even in low-resource settings and can be performed under local anesthesia. It is crucial to close the fascia with a non-resorbable suture for pneumoperitoneum creation and maintain regular wound care. We found that a BTA injection at three points on each side with a total dose of 300 IU was sufficient. Due to notable discomfort with injected volumes exceeding 1000 cc per day, we recommend a gradual and slower increase in volume over an extended period.

**Long-term Ex Situ Normothermic Kidney Perfusion**
ID: 106

**Kategorie: SCF - Innovative Surgical Techniques**

Richard Sousa Da Silva[1], Jonas Binz[2], Florian Huwyler[2], Matteo Müller[1], Miriam Weisskopf[3], Yannik Kalbas[4], Mark Tibbit[2], Andre Mihaljevic[5], Philipp Dutkowski[6], Pierre Alain Clavien[7], Philipp Kron[5]

[1]*USZ, Zürich, Schweiz*, [2]*ETH, Zürich, Schweiz*, [3]*Center for surgical research, Zürich, Schweiz*, [4]*Traumatologie USZ, Zürich, Schweiz*, [5]*Viszeralchirurgie, Tübingen, Deutschland*, [6]*Chirurgie, Basel, Schweiz*, [7]*Wyss Center, Zürich, Schweiz*

**Background:**
Marginal grafts are increasingly considered for transplantation due to the widening gap of available donor grafts and waiting recipients. Graft assessment and repair prior to implantation are therefore essential to minimize the risk of failure. Normothermic perfusion has not been widely evaluated for kidney grafts, with existing perfusions limited to 2 days. The aim of this study was to develop a long-term (>3 days) normothermic *ex situ* swine perfusion model in kidney grafts.

**Materials and methods:**
Swine kidneys were retrieved with minimal injury. After cannulation, organs were perfused at 37°C for up to several days in a newly designed normothermic *ex situ* device with automated blood gas and glucose regulation, addition of nutrition, antibiotics, and vasodilators. Kidney biopsies, urine and perfusate samples were taken daily for analysis as well as markers of viability.

**Results:**
In total 17 kidneys were perfused. Four kidneys were perfused for 3 or more days. Viability was maintained up to 4 days without histological evidence of glomerular and minimal to moderate tubular necrosis. We observed upregulation of ki67 and pH3 in tubular cells with mean values of up to 14.2% and 2.2% respectively. This expression of ki67, a proliferation associated protein, correlated with the duration of *ex situ* perfusion.

**Conclusion:**
This is the first report of normothermic kidney perfusion for up to 4 days with unprecedented observation of upregulated ki67 and pH3 after multiple days of perfusion. These preclinical results open the door for development of long-term perfusion protocols for discarded human grafts while also hinting at limitations and challenges.

**Indocyanine Green Fluorescence (ICG-F)-guided surgery in Pediatric Surgery. Indications and Consensus Statement.**
ID: 354

**Kategorie: SCF - Innovative Surgical Techniques**

Philipp Szavay[1], Rebecca Rentea[2]

[1]*Luzerner Kantonsspital, Kinderspital Zentralschweiz, Kinderchirurgische klinik, Luzern, Schweiz*,
[2]*Kansas City, Children's Mercy, Section of Colorectal and Pelvic Reconstructive Surgery, Kansas City, Vereinigte Staaten*

**Background:**
Indocyanine Green Fluorescence (ICG-F)- guided surgery is increasingly becoming a tool in pediatric surgery. However, there is uncertainty regarding indications, usefulness, dosing, and timing of dosing. Therefore, a consensus statement investigating the utility of ICG-F -guided surgery in various pediatric surgical applications has been established by an international pediatric surgical expert group, primarily focusing on its evidence base, safety, indications, use across different surgical specialties and dosing strategies.

**Materials and methods:**
An international panel of 15 pediatric surgeons from 9 countries was assembled. The structured process consisted of a rapid scoping review, iterative discussion sessions, mixed-methods studies with key stakeholders, and voting rounds on individual statements to create draft consensus statements.

**Results:**
100 articles were identified during the review and summarized. Consensus statements were then generated after 3 iterative rounds of anonymous voting. Key areas were quality of evidence, the safety of ICG, pediatric surgical indications, utilization per surgical specialty, and dosing of ICG.

**Conclusion:**
A consensus statement could be achieved providing support for pediatric surgeons for the application of ICG-F in pediatric surgical cases based on the best available evidence, key stakeholder consultation, and expert opinions. Despite ICG-F's promising potential, the need for higher-quality evidence, prospective trials, and safety studies is underscored. The consensus also provides a framework for pediatric surgeons to utilize ICG-F effectively.

**Evaluation of Parenchymal Transection Techniques on Intraoperative Blood Loss in Liver Resection: Insights from a Porcine Model with Elevated Central Venous Pressure**
ID: 594

**Kategorie: SCF - Innovative Surgical Techniques**

Felicia Kneifel[1], Annika Mohr[1], Alexander Bungert[1], Haluk Morgül[1], Philipp Houben[1], Mazen Juratli[1], Andreas Andreou[1], Shadi Katou[1], Andreas Pascher[1], Benjamin Strücker[1], Felix Becker[1]

[1]*Universitätsklinikum Münster, Allgemein-, Viszeral- und Transplantationschirurgie, Münster, Deutschland*

**Background:**
Liver resection is the standard treatment for resectable liver tumors and metastases. Advances in perioperative care, anesthesia, and surgical techniques have made major liver resections feasible, even in patients with significant comorbidities, such as liver cirrhosis or pulmonary hypertension. However, mortality and morbidity remain significant concerns in high-risk patients, and the optimal parenchymal transection technique for patients with chronically elevated central venous pressure (CVP) remains unclear.

**Materials and methods:**
This study utilized an innovative porcine model to simulate high-CVP conditions during liver resection. Animals were assigned to two groups: a control group with a CVP of ≤ 5 mmHg (low-CVP, n=10) and an intervention group with a CVP of ≥ 10 mmHg (high-CVP, n=15). Left lateral liver resection was performed using three different transection techniques: clamp-crush (CC), harmonic scalpel (HS), and stapler (ST). The primary endpoint was intraoperative blood loss. Secondary endpoints included parenchymal transection time and bile leakage from the resection surface.

**Results:**
Statistical analysis revealed no significant differences in blood loss or transection time among the low-CVP subgroup. In contrast, within the high-CVP group, both the HS and ST techniques resulted in significantly lower blood loss and shorter transection times compared to the CC technique. Parenchymal transection times were comparable between the low- and high-CVP cohorts for the HS and ST techniques; however, transection times were significantly longer with the CC technique in the high-CVP group. The incidence of bile leakage did not significantly differ between the low- and high-CVP cohorts across all resection techniques.

**Conclusion:**
This pilot study indicates a trend favoring precise dissection techniques, particularly HS and ST, in high-CVP liver resections. The findings from this translational large animal model provide a solid foundation for further investigation into optimal liver resection techniques for patients with chronically elevated CVP, establishing a crucial link between preclinical research and clinical application.

**Intestinal Alkaline Phosphatase Orchestrates Autophagy to Alleviate Hepatocellular Lipid Accumulation in Non-Alcoholic Fatty Liver Disease**
ID: 308

**Kategorie: SCF - Novel therapeutics, Biomarker, Targeted Therapy**

Lingxuan An[1], Ulrich Wirth[1], Josefine Schardey[1], Joachim Andrassy[1], Matthias Ilmer[1], Frank Braun[1], Jens Werner[1], Florian Kühn[1]

[1]*LMU klinikum, Munich, Deutschland*

**Background:**
Dysregulation of autophagy is recognized as a pivotal mechanism in the pathogenesis of non-alcoholic fatty liver disease (NAFLD). The induction of autophagy has emerged as a prospective therapeutic avenue for mitigating lipid deposition within hepatocytes. Intestinal alkaline phosphatase (IAP), a brush border enzyme implicated in treating liver fibrosis in murine models, has demonstrated autophagy-inducing effects in intestinal epithelial cells and macrophages. In this study, we explore the potential of IAP to induce autophagy and ameliorate lipid accumulation in HepG2 cells, establishing a foundation for its therapeutic utility in NAFLD.

**Materials and methods:**
Bovine-derived IAP was applied to HepG2 cells to investigate its impact on autophagic activity. Autophagy induction was assessed by monitoring LC3B-II expression through Western blot analysis, and the formation of autophagosomes was detected using monodansylcadaverine (MDC) staining. To replicate the conditions of fatty liver, a cellular model was established by exposing HepG2 cells to a combination of oleic acid and palmitic acid. Intracellular lipid deposition was quantified using Nile Red staining via flow cytometry.

**Results:**
Western blot results demonstrated a significant induction of autophagic activity in HepG2 cells following IAP treatment, particularly evident after a 24-hour exposure and at a concentration of 50U/ml (4.61±2.08 vs. 1.00±0.00, p=0.0223). Simultaneously, MDC staining showed that compared with the control group, the IAP-treated group displayed a notable increase in the accumulation of autophagosomes (24.47±6.05 vs. 7.23±4.55, p=0.0035). Flow cytometry analysis revealed a significant reduction in lipid deposition within the fatty liver cell model upon IAP treatment (166.6±13.87 vs. 212.5±11.89, p=0.0012). Notably, this effect was reversed with the introduction of 3-methyladenine (3-MA), an autophagy inhibitor.

**Conclusion:**
IAP has an effect in reducing lipid deposition in hepatocytes, with the induction of autophagy identified as a potential underlying mechanism. Our findings underscore the promising therapeutic role of IAP in treating NAFLD.

**Identification of predictive surrogate markers for CD8+ T-cell infiltration in soft tissue sarcoma: the role of ECOG, CRP, leukocyte counts, Ki-67 and tumor staging**
ID: 398

**Kategorie: SCF - Novel therapeutics, Biomarker, Targeted Therapy**

Adrian Sagebiel[1], Ruba Al Shonikat[1], Simone Schewe[1], Thilo Hackert[1], Nicola Gagliani[1], Anna Dupree[1]

[1]*Klinik für Allgemein-, Viszeral- und Thoraxchirurgie - Universitätsklinikum Hamburg-Eppendorf - UKE, Hamburg, Deutschland*

**Background:**
Soft-tissue sarcomas are a heterogeneous group of malignancies arising from mesenchymal tissues, often characterized by aggressive behavior and limited treatment options. Despite advances in systemic therapies, outcomes remain poor for advanced-stage disease, underscoring the need for novel therapeutic strategies. Immunotherapies have not yet been fully established in soft-tissue sarcoma treatment and currently show highly variable response rates. Therefore, identifying predictive surrogate markers for CD8+ T-cell infiltration, a key factor in antitumor immunity, is essential. In this study, we conducted a comprehensive exploratory analysis to identify clinical surrogate markers that correlate with CD8+ T-cell infiltration in sarcoma patients. Among the wide range of markers analyzed, the ECOG performance status, C-reactive protein (CRP), preoperative leukocyte counts, Ki-67 expression, and clinical tumor staging (cT) emerged as the most prominent.

**Materials and methods:**
We performed an exploratory analysis of extensive clinical and FACS data from sarcoma patients who underwent tumor resection. Data collected included ECOG performance status, preoperative CRP levels, leukocyte counts, Ki-67 expression, clinical tumor stage (cT), and detailed profiling of tumor-infiltrating CD8+ T cells, with a focus on CD8 PD1+ expression. Statistical analyses such as ANOVA and Pearson correlations were employed to evaluate the association of these parameters with CD8+ T-cell infiltration.

**Results:**
Our comprehensive analysis identified ECOG status, CRP, preoperative leukocyte counts, Ki-67 expression, and cT stage as the most relevant surrogate markers for CD8+ T-cell infiltration. Patients with higher ECOG scores (≥2) demonstrated significantly reduced CD8+ T-cell infiltration in their tumor microenvironment ($p = 0.045$), especially in the CD8 PD1+ subset, suggesting a correlation between poorer performance status and decreased immunogenicity.
Ki-67 expression, a marker of tumor proliferation, showed the strongest positive correlation with CD8+ T-cell infiltration ($r = 0.63$), indicating that tumors with higher proliferation rates might be associated with increased immune infiltration. The clinical tumor stage (cT) also demonstrated notable associations with CD8+ T-cell levels, suggesting that tumor burden could impact immune cell infiltration.
Additionally, elevated CRP levels were negatively correlated with CD8+ T-cell infiltration ($r = -0.34$) and CD8 PD1+ expression ($r = -0.31$), indicating that systemic inflammation may hinder effective T-cell infiltration. In contrast, higher preoperative leukocyte counts showed a positive correlation with CD8+ T-cell infiltration ($r = 0.54$), suggesting that systemic immune activation may play a role in facilitating effector T-cell tumor infiltration.

**Conclusion:**
This exploratory analysis identified ECOG performance status, CRP levels, leukocyte counts, Ki-67 expression, and cT stage as significant surrogate markers for CD8+ T-cell infiltration in sarcoma. These markers could help identify patients with a more favorable tumor immune environment, which may, in turn, suggest a greater potential for successful immunotherapy response. Further research is necessary to validate these markers and establish their role in guiding personalized treatment strategies for sarcomas.

Abstracts – DCK 2025 – 142nd Congress of the German Society of Surgery – Munich, March 26–28 • DOI 10.1515/iss-2025-2005
Innov Surg Sci 2025; 10 (Special Suppl 1): s256–s367

### Anti-fractalkine antibodies reduce tissue damage and relieve pain perception in pancreatitis by inhibiting the activation of inflammatory cells.

ID: 412

**Kategorie: SCF - Novel therapeutics, Biomarker, Targeted Therapy**

Okan Safak[1], Jan G. D´haese[1], Tom D. D´haese[1], Hamza Sezgin[1], Sergey Tokalov[1], Phillip Gärtner[1], David Jungwirth[1], Ibrahim Gürcinar[1], Steffen Teller[1], Alper Dogruoz[1], Timo Kehl[1], Frank Bergmann[2], Helmut Friess[1], Güralp Ceyhan[3], Rouzanna Istvanffy[1], Ihsan Ekin Demir[1]

[1]*TUM, Klinikum rechts der Isar, Chirurgische Klinik und Poliklinik, Munich, Deutschland*, [2]*University of Heidelberg, Heidelberg, Deutschland*, [3]*Acibadem Mehmet Ali Aydinlar University, Istanbul, Turkey*

**Background:**
The World Health Organization's (WHO) pain therapy guidelines are often inadequate for managing acute and chronic pancreatitis. This study investigates the potential of inhibiting the CX3CL1/CX3CR1 axis to enhance pain management and reduce tissue damage in pancreatitis.

**Materials and methods:**
The effects of anti-fractalkine antibodies (aFKN) on c-Fos activity in dorsal root ganglion-derived neuronal cells were initially evaluated in vitro. The impact of aFKN on abdominal pain perception was then assessed in C57BL/6 mice with cerulein-induced acute and chronic pancreatitis. Additional experiments involved CX3CR1-knockout mice. Histopathological changes in pancreatic tissue and the spinal cord's posterior horn were analyzed using immunohistochemistry and fluorescence-activated cell sorting.

**Results:**
aFKN treatment significantly reduced c-Fos expression and the secretion of inflammatory cytokines IL-6 and CCL2 in vitro. In vivo, CX3CR1-knockout mice and CX3CL1 inhibitor treated C57BL/6 mice with cerulein-induced pancreatitis, exhibited decreased pain perception and c-Fos activity in the posterior horn ganglia. Treated mice also showed reduced pancreatic tissue damage, with greater preservation of functional tissue and fewer infiltrating inflammatory cells. Lower levels of inflammatory cell-derived Cathepsin S were detected in the pancreatic tissue.

**Conclusion:**
Inhibiting the CX3CL1/CX3CR1 axis is a promising supplementary therapy option for managing pain in acute and chronic pancreatitis. This strategy disrupts macrophage activation and spinal cord microglia activity, leading to reduced c-Fos expression, decreased pain perception, and improved tissue preservation.

**Deciphering the Role of the CX3CL1-CX3CR1 Axis in Pain Perception in Pancreatitis**
ID: 414

**Kategorie: SCF - Novel therapeutics, Biomarker, Targeted Therapy**

Ibrahim Halil Gurcinar[1], Okan Safak[1], Sergey Tokalov[1], David Jungwirth[1], Phillip Gärtner[1], Alper Dogruöz[1], Helmut Friess[1], Güralp Ceyhan[2], Rouzanna Istvanffy[1], Ihsan Ekin Demir[1]

[1]*Technische Universität München , Klinikum rechts der Isar, Chirurgie, München*, [2]*Mehmet Ali Aydinlar Acibadem University, Chirurgie, Istanbul, Turkey*

**Background:**
Chronic pancreatitis (CP) is characterized by severe, persistent pain, which results from neural and inflammatory changes within the pancreas. Our previous studies have demonstrated that the CX3CL1-CX3CR1 axis plays a pivotal role in mediating neuropathic pain through interactions between immune cells and neurons. CX3CR1, expressed in spinal microglia and pancreatic macrophages, has been identified as a key player in promoting hyperalgesia during pancreatic inflammation. Blocking this axis presents a potential therapeutic target for pain relief in pancreatitis

**Materials and methods:**
In our preclinical models of acute and chronic pancreatitis, we utilized genetically engineered CX3CR1 knockout mice (CX3CR1$^{-/-}$) and evaluated the effects of fractalkine (CX3CL1) neutralization via a blocking antibody on pain perception. Pain was assessed using von Frey filament testing, while neuronal activation was measured via cFos expression in spinal cord sections. Additionally, cytokine levels (IL-6, CCL2) and immune cell infiltration in pancreatic tissues were quantified. Human CP tissue samples were also analyzed to correlate CX3CR1 expression with patient pain scores.

**Results:**
We found that neutralizing CX3CL1 significantly reduced pain behavior in both acute and chronic pancreatitis models. CX3CR1 knockout mice exhibited lower mechanical hypersensitivity as evidenced by reduced von Frey scores, which correlated with decreased cFos expression in the dorsal horn of the spinal cord. Furthermore, fractalkine neutralization led to a significant reduction in IL-6 and CCL2 secretion from DRG neurons, as well as a decrease in macrophage infiltration in the pancreas. In human CP samples, elevated CX3CL1 expression in pancreatic tissues was strongly associated with higher pain scores.

**Conclusion:**
Our data suggest that the CX3CL1-CX3CR1 axis plays a critical role in mediating pancreatitis-associated pain through immune-neuron interactions. Inhibition of this signaling pathway not only attenuates pain but also reduces inflammation in the pancreas. These findings support the CX3CL1-CX3CR1 axis as a promising target for future therapeutic interventions aimed at reducing pain and improving quality of life in patients with chronic pancreatitis.

**Importance of the genetic marker TP53 for therapy response and prediction of prognosis in patients with peritoneal carcinomatosis from colorectal or appendiceal origin Short title: TP53 is a predictive marker in peritoneal carcinosis**
ID: 477

**Kategorie: SCF - Novel therapeutics, Biomarker, Targeted Therapy**

Tamara Braunschmid[1 2], Sonja Kappel-Latif[2], Martina Mittlböck[3], Andrea Beer[4 5], Daniela Kandioler[2]

[1]*Klinik Floridsdorf, WIGEV, Chirurgie, Wien, Österreich*, [2]*Medzinische Universität Wien, Chirurgie, Wien, Österreich*, [3]*Medizinische Universtität Wien , Statistik, Wien, Österreich*, [4]*Labor Kaserer, Koperek & Beer, Pathologie, Wien, Österreich*, [5]*Medizinische Universtität Wien, Pathologie, Wien, Österreich*

**Background:**
Peritoneal carcinomatosis (PC) from colorectal cancer (CRC) or appendiceal cancer (ACA) indicates progressive disease and correlates with an unfavorable prognosis. Cytoreductive surgery (CRS) and hyperthermic intraperitoneal chemotherapy (HIPEC) became the standard treatment for selected patients; nevertheless, early recurrence remains a notable problem. Mutation of TP53 is known to be associated with poor prognosis in CRC patients, although, routine analysis is not common yet. In 2015 TP53 was validated to be a predictive marker in stage III colon cancer patients. The present study aims to validate the predictive power of TP53 in patients with PC of colorectal or appendiceal origin undergoing CRS+HIPEC.

**Materials and methods:**
In this retrospective single-center cohort study we analyzed histological specimen of 62 patients who underwent CRS+HIPEC due to PC of colorectal or appendiceal origin. The TP53 marker status was assessed using the protocol developed by the Medical University of Vienna p53research group.

**Results:**
Median follow up of the cohort was 64.3 months. Synchronous metastases occurred more often in patients with ACA and the peritoneal cancer index (PCI) was significantly higher. TP53 was mutated more frequently in CRC patients. Male gender had a significantly bad influence on early recurrence but not on OS. CRC patients with MSI, synchronous metastases or more invasive surgical procedures had worse DFS; KRAS wt was a significant risk factor for worse OS. ACA patients with metachronous metastases or neoadjuvant chemotherapy and inactive TP53 showed a significantly higher rate of early recurrence.

**Conclusion:**
The analysis was able to show that the TP53 marker status has a significant influence on prediction of therapy response in patients undergoing CRS+HIPEC. ACA patients with TP53 wt had a significantly worse DFS when neoadjuvant chemotherapy was applied. Also in patients with CRC and inactive TP53 neoadjuvant chemotherapy showed a negative effect on DFS. In the ACA subgroup where no neoadjuvant chemotherapy was given the TP53 mutational status was able to predict CRS+HIPEC therapy response; inactive TP53 was associated with significantly worse DFS.

**Diabetes Type 2 and Obesity are regulated by the mTOR pathway**
ID: 533

**Kategorie: SCF - Novel therapeutics, Biomarker, Targeted Therapy**

Laura Feiner[1], Jonas Zimmer[2], Matthias Glanemann[2], Claudia Rubie[1]

[1]*Universität des Saarlandes, Klinik für Allgemein-, Viszeral-, Gefäß- und Kinderchirurgie, Homburg, Deutschland*, [2]*Universitätsklinikum des Saarlandes, Klinik für Allgemein-, Viszeral-, Gefäß- und Kinderchirurgie, Homburg, Deutschland*

**Background:**
Mechanistic target of rapamycin (mTOR) regulates lipid and glucose metabolism thus playing a key role in metabolic diseases like type 2 diabetes mellitus (T2DM). Recently, we demonstrated a functional interaction of miRNA-496 (miR-496) with mTOR and its impact on the regulation of human ageing. As T2DM is most prevalent in older adults, we hypothesized that miR-496 may also have an impact on mTOR regulation in T2DM.

**Materials and methods:**
Based on real time PCR and enzyme-linked immunosorbent assay (ELISA) mTOR gene and protein expression as well as miR-496 expression was monitored in Peripheral Blood Mononuclear Cells (PBMC) from T2DM patients (median age: 71) and healthy age- and BMI matched controls (median age: 69). Further the mTOR protein- and miR-496 expression was determined in T2DM patients with different degrees of obesity.

**Results:**
Our data indicate miR-496 involvement in the regulation of T2DM through the control of mTOR. We further demonstrate that this interaction may be dependent on the patients body mass index with significantly elevated mTOR activity reflecting the progression of the underlying obesity disorder.

**Conclusion:**
Monitoring mTOR expression may be considered as a marker for T2DM development and the progression of obesity. As mTOR up-regulation caused by obesity maintains circulating fatty acids within the physiological range, the BMI-dependent mTOR up-regulation may be considered as a compensatory mechanism to protect the body from metabolic harm.

**Prognostic value of circulating tumor DNA and total tumor volume in patients with unresectable colorectal cancer liver metastases**
ID: 546

**Kategorie: SCF - Novel therapeutics, Biomarker, Targeted Therapy**

Nerma Crnovrsanin[1 2], J Michiel Zeeuw[3 4], Mahsoem Ali[3 4], Ruby Kemna[3 4], Nina Wesdorp[3 4], Jan van Waesberghe[4 5], Janneke van den Bergh[4 5], Irena Nota[4 5], Shira Moos[4 5], Marinde Bond[6], Iris van 't Erve[7], Alessandro Leal[8], Nic Dracopoli[8], Victor E. Velculescu[9], Lana Meiqari[2], Iris Huitnik[2], Elisa Giovannetti[4], Jaap Stoker[4 10], Inez Verpalen[4 10], Joost Huiskens[3], Gerrit A. Meijer[2], Rutger-Jan Swijnenburg[3 4], Cornelis J.A. Punt[6], Niels Fm Kok[11], Geert Kazemier[3 4], Remond J.A. Fijneman[2]

[1]*Universitätsklinikum Heidelberg, Department of General, Visceral and Transplantation Surgery, Heidelberg, Deutschland*, [2]*The Netherlands Cancer Institute (NKI), Department of Pathology, Amsterdam, Niederlande*, [3]*VU University Medical Center, Department of Surgery, Amsterdam, Niederlande*, [4]*Amsterdam UMC - Cancer Center Amsterdam, location VUmc, Amsterdam, Niederlande*, [5]*VU University Medical Center, Department of Radiology and Nuclear Medicine, Amsterdam, Niederlande*, [6]*Julius Center for Health Sciences and Primary Care, Utrecht, Niederlande*, [7]*Stanford Cancer Institute, Palo Alto, Vereinigte Staaten*, [8]*Delfi Diagnostics, Baltimore, Vereinigte Staaten*, [9]*Sidney Kimmel Comprehensive Cancer Center, Baltimore, Vereinigte Staaten*, [10]*Academisch Medisch Centrum, Department of Radiology and Nuclear Medicine, Amsterdam, Niederlande*, [11]*The Netherlands Cancer Institute (NKI), Department of Surgery, Amsterdam, Niederlande*

**Background:**
Liver metastases are a leading cause of mortality in colorectal cancer. Current decisions for managing colorectal cancer liver metastases (CRLM) rely heavily on technical resectability. However, CRLM represent a complex disease with high molecular heterogeneity, which are currently not part of the standard treatment decision path. The need for more reliable biomarkers that reflect tumor biology is crucial to improve treatment decision making.
The aim of this study was to evaluate the (combined) prognostic value for overall survival (OS) and early-recurrence of circulating tumor DNA (ctDNA) and total tumor volume (TTV) in patients with initially unresectable CRLM and their correlation with each other.

**Materials and methods:**
This translational study included patients with initially unresectable liver-only CRLM from the phase 3 CAIRO5 trial (NCT02162563), who received doublet chemotherapy (FOLFIRI/FOLFOX) and bevacizumab as induction chemotherapy. Patients were deemed eligible when the TTV could be calculated and blood samples were collected. TTV was measured on CT scans using a semi-automatic segmentation tool. ctDNA was assessed using a mutation-independent, cell-free DNA (cfDNA) fragmentation-based method. This represents the DELFI tumor fraction (DELFI-TF) score, indicative for the mutant allele fraction (MAF). Main outcome was the prognostic value of these biomarkers for OS and early recurrence after resection, which was evaluated using multivariable Cox and logistic regression models. Early recurrence was defined as any recurrence within 6 months after surgery.

**Results:**
In total 101 patients were included. Median baseline TTV was 139 mL (interquartile range [IQR], 23 to 497 mL) and median DELFI-TF score was 0.29 (IQR, 0.13 to 0.41). Baseline TTV and DELFI-TF score were moderately correlated with each other (Spearman's $\rho = 0.70$, 95% CI, 0.57 to 0.79). After systemic treatment, the median relative decrease in TTV was 50% (IQR, 28% to 64%) and in DELFI-TF 97% (IQR, 91% to 99%). At this timepoint, the change in TTV did not correlate significantly with change in DELFI-TF score (Spearman's $\rho = 0.09$, 95% CI, -0.01 to 0.42). In multivariable analyses DELFI-TF score ($P < 0.0001$) and baseline TTV ($P = 0.0003$) were both strong independent predictors of overall survival (OS) and outperformed current clinicopathological variables. Patients with higher baseline ctDNA levels had a worse prognosis independent of TTV. In the subgroup of resected patients, patients with early recurrences had a higher baseline TTV compared to patients without early recurrence (n=19 vs 27; median, 62 mL vs 24 mL; P=0.035), as well as a higher baseline DELFI-TF score (median, 0.31 vs 0.11; P=0.005).

**Conclusion:**
The combination of ctDNA levels and TTV provides a more comprehensive assessment of the total tumor burden and biology in patients with initially unresectable CRLM. Therefore, incorporating ctDNA testing alongside radiological volumetric tumor measurements could significantly improve the risk stratification in this patient subgroup.

**Crosstalk of the BH3-only protein NOXA and antigenicity in pancreatic cancer**
ID: 566

**Kategorie: SCF - Novel therapeutics, Biomarker, Targeted Therapy**

Denise Demic[1], Leonie Witte[1], Selen Selcen[1], Hazal Köse[2], Badeel Zaghla[2], Paolo Mazzeo[3], Raphael Koch[3], Michael Ghadimi[1], Günter Schneider[1 4 5], Martin Klatt[2], Matthias Wirth[1 2 6 7]

[1]*Department of General, Visceral and Pediatric Surgery, University Medical Center Göttingen, Göttingen, Deutschland*, [2]*Department of Hematology, Oncology and Cancer Immunology, Campus Benjamin Franklin, Charité - Universitätsmedizin Berlin, Berlin, Deutschland*, [3]*Department of Hematology and Medical Oncology, University Medicine Göttingen, Göttingen, Deutschland*, [4]*CCC-N (Comprehensive Cancer Center Lower Saxony) , Göttingen, Deutschland*, [5]*Clinical Research Unit KFO5002, University Medical Center Göttingen, Göttingen, Deutschland*, [6]*German Cancer Consortium (DKTK), Heidelberg, Deutschland*, [7]*Max Delbrück Center, Berlin, Deutschland*

**Background:**
Even though recent advancements in targeted therapy and immunotherapy have contributed to improving the prognosis of multiple malignancies, pancreatic cancer remains unresponsive to both standard of care treatment and immunotherapies. Various mechanisms of immune evasion such as blocking infiltration of cytotoxic T cells (CTL) into tumor tissue and loss of antigenicity (e.g., MHC-I), preventing T cell recognition contribute to therapy resistance. The immunogenically silent cell death mode apoptosis is commonly deregulated in pancreatic cancer and is controlled by the BCL2 protein family, which is composed of pro- and antiapoptotic proteins. We recently identified an enrichment of the pro-apoptotic BH3-only NOXA in the aggressive and therapy resistant basal-like pancreatic cancer subtype. To investigate the role of therapy resistance we aim to dissect the crosstalk of NOXA-associated apoptosis and antigenicity in pancreatic cancer.

**Materials and methods:**
A computational approach using CIBERSORTx helped to identify virtual *NOXA*-associated immune cell infiltration pan-cancer using the TCGA repository. To investigate the potential effect of NOXA on antigenicity in pancreatic cancer, we analyzed MHC-I presentation in isogenic parental and *NOXA* knockout pancreatic cancer cell lines via flow cytometry and immunoblotting. In addition, we performed immunopeptidomics to investigate *NOXA*-associated (neo-)antigen presentation. To mechanistically dissect the crosstalk of NOXA-associated apoptosis and antigenicity, we performed BH-3 profiling and an unbiased drug screen using 1812 drugs in 4 isogenic *NOXA* knockout cell lines. We validated the top drug hit in *NOXA*-deficient cells by viability assays, MHC-I flow cytometry, and immunoblotting. Additionally, we performed transcriptomic profiling using RNAseq in *NOXA*-proficient and deficient cells and integrated our results using data from publicly available repositories.

**Results:**
Bulk RNAseq analysis uncovered a highly significant negative correlation between infiltration of CTLs and *NOXA* expression in pancreatic cancer. Notably, this was the top negative correlation observed across all cancer types within the TCGA dataset, thus uncovering a specific vulnerability of pancreatic cancer. This prompted a further investigation *in vitro*. Here, upon *NOXA* deletion, pancreatic cancer cells showed an increased MHC-I expression and a unique repertoire of antigens presented on the cell surface. Additionally, we identified a specific vulnerability of *NOXA*-deficient cells to Chk1 inhibition, indicating a crosstalk of DNA damage repair and antigenicity, as detected by an unbiased drug screen. Transcriptomic profiling of *NOXA*-proficient and deficient cells, treated with a highly specific Chk1 inhibitor revealed an induction of the antigen presentation machinery and a significant correlation to DNA damage repair associated gene signatures. Integration of our data with publicly available repositories verified the association of NOXA and DNA-damage-inducing antigenicity in pancreatic cancer.

**Conclusion:**
We identified the pro-apoptotic BH3-only protein NOXA as a potential marker for more concise patient stratification: Our results indicate that low *NOXA* expression can be associated with higher MHC-I expression, CTL infiltration, and increased susceptibility to Chk1 inhibition. DNA-damaging therapeutics have been found to synergize with immunotherapy and a higher MHC-I expression, which may further enhance immunorecognition through a more profound presentation of (neo-)antigens, supporting our findings. We here, show an unexcpected role of NOXA in the immunogenicity of pancreatic cancer, paving the way for a more concise patient stratification and allowing for the development of improved combinatorial treatment regimens.

**Literature**

Brahmer JR, et al. (2012): Safety and Activity of Anti–PD-L1 Antibody in Patients with Advanced Cancer. N Engl J Med 366, 2455–2465

Brown JS, Sundar R, Lopez J (2018): Combining DNA damaging therapeutics with immunotherapy: more haste, less speed. Br J Cancer 118, 312–324

Doffo J, et al. (2022): NOXA expression drives synthetic lethality to RUNX1 inhibition in pancreatic cancer. Proc Natl Acad Sci USA 119, e2105691119

Kleeff J, et al. (2016): Pancreatic cancer. Nat Rev Dis Primers 2, 16022

Newman AM, et al. (2019): Determining cell type abundance and expression from bulk tissues with digital cytometry. Nat Biotechnol 37, 773–782

Rahib L, et al. (2021): Estimated Projection of US Cancer Incidence and Death to 2040. JAMA Netw Open 4, e214708

Siegel RL, Giaquinto AN, Jemal A (2024): Cancer statistics, 2024. CA A Cancer J Clinicians 74, 12–49

Wu X, et al., (2023): Targeting MHC-I molecules for cancer: function, mechanism, and therapeutic prospects. Molecular Cancer 22, 194

**Histone deacytelase inhibitiors are a valuble addition to therapy regimes in primary and metastatic colorectal cancer.**
ID: 612

**Kategorie: SCF - Novel therapeutics, Biomarker, Targeted Therapy**

Sebastian Schmitz[1], Rebecca Ulbrich[1], Jens Werner[1], Barbara Mayer[1]

[1]*Department of General, Visceral and Transplantation Surgery, LMU University Hospital, LMU Munich, Munich, Deutschland*

**Background:**
Over one-third of colorectal cancer (CRC) patients with primary, locally advanced tumors do not reach the five-year survival despite radical surgery followed by intense chemotherapy. In the metastatic stage survival drops to around 15%, despite a wider range of available therapies.
Additionally, nearly half of non-metastatic CRC patients need to de-escalate from a FOLFOX chemotherapy regimen to a 5-FU monotherapy due to severe oxaliplatin-related toxicities. This underscores the urgent need for new treatment strategies, as well as a significant opportunity for improvement, for example, by replacing Oxaliplatin with an equally effective but less toxic alternative. Candidates are Histone Deacetylase (HDAC) inhibitors (HDACi), which are FDA-approved (e.g., vorinostat, panobinostat) and BfArM-approved (panobinostat) for the treatment of hematological malignancies. This study aims to investigate the effect of the pan-HDAC inhibitors vorinostat and panobinostat in the patient-derived CRC spheroid model.

**Materials and methods:**
Effects of HDACi were tested in a 3D diagnostic platform. Multicellular cancer spheroids were prepared from 28 primary colorectal cancer tissues. After 48h spheroids were treated with either vorinostat (Vor) or panobinostat (Pan) as single agent or in combination with the standard chemotherapeutics 5-FU, 5-FU+Oxaliplatin (FO) or 5-FU with Irinotecan (FI) at the peak plasma concentration for 72h. Efficacy of the HDACi was evaluated by treatment-induced changes in spheroid morphology using a semi-automated algorithm. In addition, impact of HDACi on spheroid metabolic activity was measured using a luminescence-based ATP assay. Statistical analysis was performed using the Mann-Whitney U test.

**Results:**
Vorinostat as a single agent was less effective than F, FO, or FI (all $p<0.001$). The combination of Vor with F was more effective than F or FI ($p<0.001$). Vor + F was equally effective as FO ($p=0.0542$) or FOI ($p=0.696$). The combination was more effective than Vor alone ($p<0.001$).
The combination of FI with Vor showed similar results. This combination was better than F and FI ($p<0.001$) and showed similar efficacy to FO or FOI ($p=0.115$, $p=0.160$). It was as effective as F + Vor ($p=0.452$).
FO with Vor showed similar efficacy to F + Vor and FI + Vor ($p=0.050$, $p=0.400$). The FO + Vor therapy was more effective compared to all standard chemotherapies (all $p\leq0.009$).
Pan alone was as effective as F in monotherapy but less effective than FO, FI, or FOI ($p<0.001$, $p=0.048$, and $p<0.001$, respectively). Combining Pan with F showed no additional benefit compared to F alone ($p=0.077$). F + Pan was as potent as FO, FI, and FOI ($p=0.945$, $p=0.219$, and $p=0.758$) and had similar efficacy to Pan monotherapy ($p=0.056$).
The combination of Pan with FO was more effective compared to F, FI, or Pan alone ($p=0.008$, $p=0.025$, and $p=0.016$). However, it showed no advantage over FO, FOI, or F + Pan ($p=0.598$, $p=0.716$, and $p=0.548$). The combination of FI with Pan only showed benefits over F, FI, and Pan as monotherapy ($p=0.019$, $p=0.043$, and $p=0.032$) but not when compared to F, FOI, F + Pan, or FO + Pan ($p=0.697$, $p=0.801$, $p=0.841$, and $p=0.548$).
Vorinostat as single agent was equally effective to panobinostat as single agent ($p=0.575$) as well as

F + Vor and F + Pan had the same efficacy (p=0.542).

**Conclusion:**
The results suggest that HDAC inhibitors (HDACi) could be a valuable addition to therapeutic regimens in colorectal cancer (CRC). Vorinostat appears to be a suitable partner for 5-FU and has the potential to replace Oxaliplatin in chemotherapy regimens. This could provide access to effective chemotherapies for a broader cohort of patients, including older patients and those with pre-existing conditions, who currently only qualify for 5-FU monotherapy. These patients could then receive a therapy comparable to FO.
In contrast, panobinostat could not yield a significant benefit when combined with chemotherapy but seems to be a viable alternative to 5-FU as monotherapy. This could offer a new treatment option with fewer side effects for patients who are currently limited to 5-FU monotherapy.
Currently, several new and more specific HDAC inhibitors are in development, which have the potential to further improve therapeutic efficacy.
In conclusion, HDAC inhibitors offer an opportunity not only for primary CRC patients, with not only potentially fewer or more tolerable side effects, but also in the metastatic setting, where a more effective chemotherapy standard such as FO could still be preserved compared to FI.

**Patient-derived microtumors highlight the potential of precision medicine in managing pancreatic ductal adenocarcinoma**
ID: 624

**Kategorie: SCF - Novel therapeutics, Biomarker, Targeted Therapy**

Christine Nitschke[1], Charline Phan[2], Philipp Walter[1], Mara Goetz[1], Gediminas Simkus[2], Jacob Thastrup[2], Jürgen Kupper[2], Jakob Izbicki[1], Thilo Hackert[1], Harriet Wikman[3], Marianne Sinn[4], Faik G. Uzunoglu[1], Tabea Sturmheit[2]

[1]*Department of General, Visceral and Thoracic Surgery, University Medical Center Hamburg-Eppendorf, Hamburg, Deutschland*, [2]*2cureX, Copenhagen, Denmark*, [3]*Institute of Tumor Biology, University Medical Center Hamburg-Eppendorf, Hamburg, Deutschland*, [4]*II. Medical Clinic (Department of Oncology), University Medical Center Hamburg-Eppendorf, Hamburg, Deutschland*

**Background:**
Pancreatic ductal adenocarcinoma (PDAC) ranks among the most lethal cancers, with only 20% of patients qualifying for curative treatment at diagnosis. Three-dimensional microtumors capturing patient-specific features of PDAC serve as a valuable disease model.

**Materials and methods:**
We employed this technology to assess drug sensitivities of patient-derived microtumors to clinically relevant drugs and combinations, evaluated culture success rates and correlated in-vitro data with clinicopathological and follow-up information. Microtumor cultures were established from PDAC patients undergoing surgical resection (or a liver biopsy at a single medical center) and being subsequently followed-up. Patient-derived cultures displaying sustained growth were analyzed regarding their phenotypic subtype and utilized for functional drug sensitivity testing (f-DST) based on the IndiTreat® technology (2cureX).

**Results:**
Correlative analyses of our PDAC patient cohort (n=67; n=42 patients with curative tumor resection and n=25 palliative patients) revealed a link between microtumor growth and reduced patient survival. Furthermore, drug sensitivity profiles (obtained of 10 patient-derived cultures) revealed notable inter-individual differences and mirrored clinical responses to administered drug therapies. f-DST was applicable across microtumor cultures of basal-like, classical and mixed subtypes. This pilot study confirms the feasibility of deriving and maintaining microtumor cultures from heterogeneous samples. Cultures displaying sustained proliferation correlated positively with advanced-stage tumors (UICC stages III and IV). Individual patient case analyses integrating in-vitro drug sensitivity profiles with clinical follow-up data suggest that f-DST using microtumors could guide future therapeutic strategies.

**Conclusion:**
In summary, microtumors offer insights into patient-specific responses to treatment, highlighting the potential of precision medicine in managing this challenging cancer.

**Modulation of P2X4 receptor activity affects the expression and release of pro- and anti-inflammatory cytokines by innate immune cells**
ID: 638

**Kategorie: SCF - Novel therapeutics, Biomarker, Targeted Therapy**

Lisa Bengs[1], Marie Dippel[1], Luca Ettischer[1], Anna Herbolsheimer[1], Andreas Hecker[1], Juliane Liese[1], Fritz Markwardt[2], Günther Schmalzing[3], Christa Müller[4], Veronika Grau[1], Katrin Richter[1 5]

[1]*Justus-Liebig University of Giessen, Laboratory of Experimental Surgery, Giessen, Deutschland*, [2]*Martin-Luther-University, Julius-Bernstein-Institute for Physiology, Halle, Deutschland*, [3]*RWTH Aachen University, Aachen, Deutschland*, [4]*University of Bonn, Bonn, Deutschland*, [5]*Bonn-Rhein-Sieg University of Applied Sciences, Rheinbach, Deutschland*

**Background:**
Pro-inflammatory cytokines that regulate innate immunity can be induced by pathogen-associated molecular patterns such as lipopolysaccharide (LPS) and play essential roles in host defense against infection. In response to several stimuli, including major surgery and accidental trauma, damaged cells release ATP, inducing P2X7 receptor (P2X7R) activation in innate immune cells. This leads to the release of a large number of cytokines, including the pro-inflammatory cytokine interleukin (IL)-1β, which can cause life-threatening, systemic inflammation. Despite the well-recognized role of the P2X7R in the assembly of the NLRP3 inflammasome and IL-1β release, it remains unclear whether other purine receptors can contribute to or modulate this inflammatory process. In this study, we examine the role of P2X4R in cytokine secretion and biosynthesis.

**Materials and methods:**
Human monocytic THP1-cells, THP1-cell-derived macrophages, primary human peripheral blood mononuclear cells (PBMCs), human macrophages derived from PBMCs, and peritoneal human macrophages were primed with LPS. Cells were then left untreated or treated with a panel of P2X4R or P2X7R antagonists and the expression and release of pro-inflammatory (IL-1β, IL-6) and anti-inflammatory (IL-10) cytokines, as well as expression of P2X4R and P2X7R was analyzed by real-time RT-PCR, Western blot and ELISA. LPS-primed cells were also stimulated with ATP to induce secretion of NLRP3 inflammasome-dependent (IL-1β, IL-18) cytokines, which were measured by ELISA.

**Results:**
Our data show that both the P2X4R and P2X7R seem to be involved in the ATP-induced IL-1β release by multiple types of mononuclear phagocytes, a typical hallmark of traumatic cell damage. Specifically, our data show that modulation of P2X4R activity can affect the LPS-induced expression and release of both, the NLRP3-dependent (IL-1β, IL-18) and -independent cytokines (IL-6, TNFα, IL-10). We identified different sensitivities and responses to P2X4R inhibition in monocytes relative to macrophages that may be explained by a 160-fold higher expression level of P2X4R in monocytes. Interestingly, blocking the P2RX4 seems to induce the activity of caspase-3/-7. While caspase-3/-7 activity is an initiator of apoptosis, it further can regulate a variety of signaling processes from proliferation to wound healing.

**Conclusion:**
These findings highlight an unexpected role of P2X4R in the expression and release of cytokines by mononuclear phagocytes. We propose that this paves the way towards novel therapeutic strategies protecting surgical and traumatized patients against infection, while efficiently preventing ATP-mediated hyperinflammation.

**Heat shock protein 90 α/β overexpression stratifies primary colorectal cancer (UICC IIb-IV) for early recurrence, short survival and response to targeted therapy.**
ID: 644

**Kategorie: SCF - Novel therapeutics, Biomarker, Targeted Therapy**

Sebastian Schmitz[1], Jacob Gülden[1], Cassandra Eichner[1], Jens Werner[1], Barbara Mayer[1]

[1]*Department of General, Visceral and Transplantation Surgery, LMU University Hospital, LMU Munich, Munich, Deutschland*

**Background:**
Benefit in targeted therapy is limited in metastatic colorectal cancer (CRC). A promising new candidate for targeted therapy are cytosolic, heat shock protein 90α and heat shock protein 90β (Hsp90α/β). This study tested the prognostic and predictive impact of Hsp90α/β protein expression in 89 primary (p) CRC patients.

**Materials and methods:**
Eighty-nine samples of pCRC were immunohistochemically stained and the results were correlated with 10-year survival data. The effectiveness of Hsp90 inhibitors e.g., ganetespib (Gan) and pimitespib (Pim) was analyzed in CRC cell lines (HCT-116; HT-29 and Caco-2) and the Patient Derived Colorectal Cancer Spheroid (PDCS) model. Additionally, the combination of Hsp90 inhibitors with standard chemotherapeutics e.g 5-FU (F), 5-FU+Oxaliplatin (FO), 5-FU+Irinotecan (FI), Encorafenib and Trastuzumab were tested.

**Results:**
Hsp90α/β overexpression (>40% positive cancer cells) was found in 27% of patients and predicted early recurrence (<16 months, p=0.047), poor progression-free survival (HR 3.250, p=0.007) and overall survival (HR 3.029, p=0.022). Hsp90α/β overexpression, when combined with BRAF-V600E mutation or Her2/neu Score >3, was linked to a worse prognosis (all p<0.05).
Hsp90 inhibitors Gan and Pim as single agents showed direct anti-tumor effects in CRC cell lines HCT-116, HT-29, and Caco-2 but were less effective than traditional chemotherapies. Combining Gan or Pim with Encorafenib or Trastuzumab improved efficacy compared to monotherapies in HT-29 and Caco-2 cells (all p<0.05).
Gan and Pim also had a chemosensitizing effect, enhancing the efficacy of 5-FU, FO or FI in HCT-116, HT-29, and Caco-2 cells (all p<0.05). F+Gan or F+Pim combinations outperformed standard chemotherapies.
In patient-derived spheroids, Hsp90α/β expression correlated with response to Hsp90 inhibitors. In those individual spheroid models the superiority of F+Gan and F+Pim to F, FI, Gan, Pim and most interesting to FO could be confirmed.

**Conclusion:**
Hsp90α/β overexpression represents a prognostic and predictive biomarker for locally advanced and metastatic CRC and could potentially replace Oxaliplatin in chemotherapy regimens.

### PRAY – a prospective, observational study for assessing risk factors for intestinal anastomosis leakage

ID: 757

**Kategorie: SCF - Novel therapeutics, Biomarker, Targeted Therapy**

Maximilian Denzinger[1], Kira Steinkraus[2], Marko Kornmann[1], Benno Traub[1 3]

[1]*Universitätsklinikum Ulm, Klinik für Allgemein- und Viszeralchirurgie, Ulm, Deutschland*, [2]*Universitätsklinikum Hamburg-Eppendorf, Klinik und Poliklinik für Allgemein-, Viszeral- und Thoraxchirurgie, Hamburg, Deutschland*, [3]*Universitätsklinikum Tübingen, Klinik für Allgemeine, Viszeral- und Transplantationschirurgie, Tübingen, Deutschland*

**Background:**

With nearly 16 million surgeries performed in Germany in 2020 and almost 17.000 oncologic surgeries, the reduction of the perioperative complication rate has high medical and economical relevance. Intestinal anastomotic leakage is one of the most relevant complications in the field of visceral and general surgery with high impact on the mortality of patients undergoing surgery.

In patients undergoing major abdominal surgery, an activation of pro- and anti-inflammatory cells and cascades resulting in an alteration of adaptive and innate immunity can be observed. The patient's response towards this trauma is highly individual. While some patients present lightly elevated inflammatory parameters, others, undergoing the same surgery, show a massive immune dysregulation and activation leading to conditions comparable to septic shock called the systemic inflammatory response syndrome (SIRS). Patients with the clinical presentation of SIRS show higher mortality and morbidity after surgical trauma.

Yet, we are not able to detect patients with a higher risk for developing SIRS before surgery and therefore cannot intervene early and adequately. Malignant disease has been shown to severly alter the patients immune system and thus we believe that especially tumor patients would benefit from a preoperative risk stratification to minimize perioperative complications.

We hypothesize that a subset of patients already displays dysregulations of their immune system preoperatively. These dysfunctions impair physiologic wound healing and can lead to outright SIRS.

**Materials and methods:**

We conducted a translational-clinical study to establish a biomarker panel to predict anastomotic leakage in patients undergoing visceral surgery with at least one intestinal anastomosis. The study was registered at the DRKS (Deutsches Register klinischer Studien) with the register number: DRKS00029194. The first patient was enrolled in the study in August 2022.

Previous studies in patients with perioperative complications showed high anti- and pro-inflammatory mediators after surgery. We believe this immunologic imbalance exists even before surgery and could therefore be modified. In detail we analyzed leukocyte subpopulations by flow cytometry such as immunosuppressive macrophages, regulatory T-cells, myeloderived suppressor cells (MDSC) and surface markers involved in transmigrations processes as well as circulating immune mediators, such as Interleukin-6 and Interleukin-8 by ELISA and their association with perioperative complications. Blood tests were performed preoperatively and on post-operative days (POD) 1, 3, and 5. Further patients were visited on the postoperative days 1, 3 and 5. Assessed complications are graded according to the Classification of Clavien-Dindo and documented in an electronical case report file (eCRF) using the RedCap System.

**Results:**

We are pleased to present preliminary results from a closed study involving 179 enrolled patients. The study was terminated due to personnel changes within the research team. Patients underwent various types of visceral surgery, including esophagectomy (25), colon surgery (64), rectal surgery (18), gastrectomy (12), and pancreatoduodenectomy (28), among others. Of the 179 patients, 20 (11.2%)

Abstracts – DCK 2025 – 142nd Congress of the German Society of Surgery – Munich, March 26–28 • DOI 10.1515/iss-2025-2005
Innov Surg Sci 2025; 10 (Special Suppl 1): s256–s367

experienced an anastomotic leak postoperatively. This rate can be compared to published anastomotic leakage rates for various general surgery procedures. Additionally, we will present the immunological profiles—based on flow cytometry and mass spectrometric analysis of perioperative plasma probes—of these patients in comparison to matched controls without anastomotic leakage.

**Conclusion:**
In the future we hope to be able to stratify each patient's individual risk score for perioperative complications. After identification of individual risk profiles, we will focus on therapeutic strategies for modification of these risk factors, hopefully resulting in an interventional study to reduce the risk for perioperative complications.

**Liposuction can Break the Vicious Circle of Pain, Physical Inactivity, and Depression in Lipedema Patients – A Response Surface Analysis.**
ID: 824

**Kategorie: SCF - Novel therapeutics, Biomarker, Targeted Therapy**

Lena Busch[1], Fiona Kirstein[1], Matthias Hamatschek[1], Tobias Hirsch[1], Maximilian Kückelhaus[1]

[1]*Institute of Musculoskeletal Medicine, University of Münster, Plastic and Reconstructive Surgery, Münster, Deutschland*

**Background:**
Lipedema is a highly prevalent disease affecting 5-15% of women with accumulations of subcutaneous fat primarily at the legs and limbs. In contrast to adipose tissue, lipedemic tissue is characterized by pain, sensitivity to pressure, and feeling of tension in the affected areas. Lipedema can cause severe impairment in mobility and can interfere with the possibility to engage in physical activity. Adiposities is highly comorbid in lipedema patients, accentuating the relevance to enhance weight loss. Moreover, lipedema patients report significantly elevated levels of depression so that pain stands in a complex interaction with physical activity and mental health in lipedema patients. Recently, it was shown that lipedema pain can interfere with the potential of drawing benefit from engaging in physical activity, describing a vicious-circle-like negative catalyzing effect of pain and physical activity on mental health in lipedema patients. In contrast to conservative therapy (e.g., compression and lymphatic drainage) liposuction can lead to substantial symptom reduction and pain relief which might pave the way to benefit from engaging in physical activity. Thus, it was the aim of this study to investigate if the catalyzing interrelations between pain, physical inactivity, and mental health change in lipedema patients who had undergone liposuction.

**Materials and methods:**
We included n=58 lipedema patients (post-op) who had undergone liposuction at our hospital. Patients had a mean age of M=40.16 (±12.45) years and a mean BMI of M=32.62 (±6.93) kg/m2. Depression severity was measured via PHQ-9 (nine items on a four-point Likert scale). QoL was measured via WHOQOL-BREF (26 items on a six-point Likert scale) with the subscales mental, physical, social, and environmental health. Pain in the referring areas was measured via a single-item questionnaire (ten-point Likert scale). Physical activity was measured via the number of hours per week. In statistical analysis, a Response Surface Analysis (RSA) was conducted. RSA is based on polynomial regression analysis and can make synergistical and catalyzation effects visible. Data analysis was conducted via the programming language R.

**Results:**
Compared to lipedema patients who had not undergone liposuction (pre-op), the interrelations between pain, physical inactivity, and mental health significantly changed after liposuction. In post-op data, only one main effect for pain predicting lower mental health ($b1 = -6.14$; $p<0.05$) and one main effect for physical activity predicting higher physical health ($b2 = 0.252$; $p<0.05$) were observed. Moreover, the significant additive and curvilinear incongruence effects observed in the pre-op data could not be detected in the post-op analysis for all scales, i.e., depression severity, mental health, physical health, social health, and environmental health.

**Conclusion:**
Lipedema pain does not only lead to lower levels of mental health and higher depression but can also interfere with the potential to engage in physical activity, forming a vicious-circle-like interrelation. Therefore, solutions leading to long-term reduction of pain and impairment in mobility are of high relevance. In this context, liposuction can lead to substantial symptom reduction such as pain relief and might pave the way to benefit from engaging in physical activity. In this study we could demonstrate that the pain reduction after liposuction can dissolve the vicious-circle-like connection between pain, physical inactivity, and low mental health in lipedema patients. In sum, liposuction and multimodal strategies in pain treatment are of high interest to reduce pain and thus to be able to benefit from the positive effects of physical activity.

### Spontaneous hypoxia and CTCs in colorectal cancer in an *in vivo* assay

ID: 59

**Kategorie: SCF - Oncology**

Julia Geheb[1], Dennis Roth[1], Maria Wecker[1], Elisabeth Moog[1], Rui Neves[1], Christiane Driemel[1], Nikolas Stoecklein[1], Wolfram Knoefel[1], Georg Fluegen[1]

[1]*Uniklinik Düsseldorf, Klinik für Allgemein-, Viszeral- und Kinderchirurgie, Düsseldorf, Deutschland*

**Background:**

Colorectal cancer is the third most common cancer in both men and women and has the second highest mortality rate of all cancers. A major cause of death is metastasis. Circulating tumor cells (CTCs) have been shown to be a precursor to metastasis. Hypoxia in colorectal cancer is associated with more aggressive tumors, more metastases and poorer overall survival. Carbonic anhydrase IX (CAIX), an endogenous marker of hypoxia, is known to be upregulated by Hypoxia-inducible facor 1-alpha (HIF-1 α) and correlates with poor patient outcome. A link between CAIX and resistance to chemotherapy or radiotherapy has been suggested. Pimonidazole is an exogenous hypoxia marker that can be used to assess hypoxia in tissues and cells. It irreversibly binds to proteins in hypoxic milieu, allowing a snapshot of hypoxic cells to be taken.

**Materials and methods:**

We used the CAM assay (chorio-allantoic membrane assay, embryonated chicken egg) to generate human xenograft tumors from three different colorectal cancer cell lines.
First, we used immunohistochemical double staining against the hypoxia markers CAIX and Pimonidazole to detect hypoxic areas in the primary tumor.
Using different protocols with high flow fluorescence activated cell sorting (FACS) we sorted CTCs from the CAM blood and analyzed them for hypoxia markers. For this we used an immunofluorescence staining.
To test the different protocols, we performed spike experiments. For this we spiked hypoxic cell culture cells in CAM-blood. To treat the cells with hypoxia, we used a hypoxia incubation chamber that is flooded with a gas mixture consisting of 1% $O_2$, 14% $CO_2$, 85% $N_2$.

**Results:**

Spontaneous hypoxic areas could be detected in most tumors. There was no correlation between the volume or the weight and the appearance of hypoxic areas. Even small tumors showed hypoxic areas. Using different protocols for staining and FACS sorting, we were always able to detect hypoxic spiked cell culture cells, but there were differences in the efficiency of these methods. Using the most efficient method, we were able to distinguish between normoxic and hypoxic cells in spike experiments.

**Conclusion:**

It has been demonstrated that CTCs are a significant prognostic factor for cancer patients. Further investigation is required to gain a deeper understanding of the development of CTCs and the factors that promote their growth, such as hypoxia. The CAM assay represents a cost-effective, rapid and easy way to investigate colorectal cancer in vivo, while also enabling the examination of the hypoxic microenvironment. In experiments with spiked cell culture cells, we sorted the cells and analyzed them regarding the hypoxia markers CAIX and Pimonidazole.
The next step will be to analyze CTCs from in vivo tumors on the CAM. These results are still pending.

Abstracts – DCK 2025 – 142nd Congress of the German Society of Surgery – Munich, March 26–28 • DOI 10.1515/iss-2025-2005
Innov Surg Sci 2025; 10 (Special Suppl 1): s256–s367

**Validation of the PANAMA-Score for Patient Stratification and Adjuvant Therapy Sequencing after Neoadjuvant Treatment for Pancreatic Cancer**
ID: 160

**Kategorie: SCF - Oncology**

Ingmar F Rompen[1], Thomas Stoop[2], Stijn van Roessel[3], Eran Veldhuisen[3], Quisette Janssen[4], Adnan Alseidi[5], Alberto Balduzzi[6], Gianpaolo Balzano[7], Frederik Berrevoet[8], Morgan Bonds[9], Olivier Busch[3], Giovanni Butturini[10], Ammar Javed[3 3], Marco Del Chiaro[11], Kevin Conlon[12], Massimo Falconi[7], Isabella Frigerio[10], Giuseppe Fusai[13], Johan Gagniere[14], Oonagh Griffin[14], Thilo Hackert[15], Asif Halimi[16], Knut Labori[17], Giuseppe Malleo[6], Marco Vito Marino[18], Michael Bau Mortensen[19], Andrej Nikov[20], Mickael Lesurtel[21], Tobias Keck[22], Jörg Kleeff[23], Rupaly Pande[24], Per Pfeiffer[25], Daniel Pietrasz[26], Keith Roberts[24], Antonio Sa Cunha[26], Roberto Salvia[6], Oliver Strobel[27], Timo Tarvainen[28], Haneke Van Laarhoven[3], Bas Groot Koerkamp[4], Martin Loos[1], Christoph Michalski[1], Marc Besselink[3], Thomas Hank[1]

[1]*Universitätsklinikum Heidelberg, Allgemein, Viszeral und Transplantationschirurgie, Heidelberg, Deutschland*, [2]*Amsterdam, Department of Surgery, Amsterdam, Niederlande*, [3]*AmsterdamUMC, Department of Surgery, Amsterdam, Niederlande*, [4]*Erasmus UMC, Rotterdam, Niederlande*, [5]*Department of Surgery, University of California at San Francisco, San Francisco*, [6]*Department of General and Pancreatic Surgery, The Pancreas Institute, University of Verona Hospital Trust, Verona, Italien*, [7]*Pancreatic Surgery, Pancreas Translational & Clinical Research Center, San Raffaele Hospital, Mailand, Italien*, [8]*Department of General and Hepatobiliary Surgery, Gent University Hospital,, Gent, Belgium*, [9]*University of Oklahoma Health Sciences Center, Oklahoma City, Vereinigte Staaten*, [10]*Department of Surgery, Pederzoli Hospital, Peschiera, Italien*, [11]*Department of Surgery, University of Colorado Hospital, Denver, Vereinigte Staaten*, [12]*Department of Surgery, Trinity College Dublin, Trinity Centre for Health Sciences, Dublin, Ireland*, [13]*Hepatobiliary Surgery and Liver Transplantation Unit, Royal Free Hospital, London, Großbritannien*, [14]*Department of Digestive and Hepatobiliary Surgery-Liver Transplantation, University Hospital of Clermont-Ferrand, Clermont-Ferrand, Frankreich*, [15]*Hamburg Eppendorf, Hamburg, Deutschland*, [16]*Department of Surgical and Perioperative Sciences, Surgery, Umeå University, Umeå, Sweden*, [17]*Department of Hepato-Pancreato-Biliary Surgery, Oslo University Hospital, Oslo, Norwegen*, [18]*General Surgery Department, Azienda Ospedaliera Ospedali Riuniti Villa Sofia-Cervello, Palermo, Italy and Oncologic Surgery Department, P. Giaccone University Hospital, Palermo, Italien*, [19]*Department of Surgery, Odense Pancreas Center, Odense University Hospital, Odense, Odense, Denmark*, [20]*Department of Surgery, Charles University and Central Military Hospital, Prag, Czech Republic*, [21]*Department of Digestive Surgery and Liver Transplantation, Croix Rousse University Hospital, Hospices Civils de Lyon, Lyon, Frankreich*, [22]*Department of Surgery, Universitaet zu Luebeck, Lübeck, Deutschland*, [23]*Department of Visceral, Vascular and Endocrine Surgery, Martin Luther University Halle-Wittenberg, Halle (Saale), Deutschland*, [24]*Department of Surgery, University Hospital Birmingham, Birmingham, Großbritannien*, [25]*Department of Medical Oncology, Odense University Hospital, Odense, Denmark*, [26]*Department of Hepato-Biliary-Pancreatic Surgery, Liver Transplant Center, Paul Brousse Hospital, Université Paris-Sud, Université Paris-Saclay, Villejuif, Frankreich*, [27]*Department of General Surgery, Division of Visceral Surgery, Medical University, Wien, Österreich*, [28]*Department of Gastroenterological Surgery, Helsinki University Hospital, Helsinki, Finland, Helsinki, Finland*

**Background:**
The Pancreatic NeoAdjuvant Massachusetts (PANAMA)-score, which is composed of persistence of elevated CA19-9, resection margin status, remaining tumor size, and nodal involvement after neoadjuvant therapy was created to guide prognostication of patients with pancreatic cancer. Furthermore, these variables are not only prognostic but indicate remaining advanced disease and local treatment failure, thus opening the window for a possible treatment benefit for additional adjuvant treatment. Therefore, we aim to validate the prognostic value of the PANAMA-score in predicting overall survival (OS), recurrence, and treatment benefit derived from adjuvant treatment in patients after neoadjuvant treatment and resection for pancreatic cancer.

**Materials and methods:**
This study represents a retrospective international multicenter study endorsed by the European-African Hepato-Pancreato-Biliary Association (EAHPBA). Patients with pancreatic adenocarcinoma who underwent pancreatic resection after neoadjuvant treatment with FOLFIRINOX were included. Mantel-Cox regression with interaction analysis was performed to assess the treatment benefit of adjuvant chemotherapy.

**Results:**
A total of 383 were considered for final analysis of which 187 (49%), 137 (36%), and 59 (15%) had low, intermediate, and high-risk according to the PANAMA-score, respectively. A stepwise reduction of time to recurrence and OS was observed per risk-group (median OS low-risk: 48.5 months [95%CI:40.7-not reached], intermediate-risk: 27.6 months [95%CI:24.3-38.1], and high-risk: 22.3 months [95%CI:16.1-31.1], all group comparisons Log-Rank-P <0.05). While PANAMA low-risk patients did not derive an OS benefit from further adjuvant treatment (HR 1.50, 95%CI:0.92-2.50), increasing benefit was observed for patients with intermediate (HR0.58, 95%CI:0.34-0.97) and high-risk profile (HR 0.47, 95%CI:0.24-0.94) scores (p-interaction=0.008).

**Conclusion:**
Findings from this study both validate the discriminatory value of the PANAMA-score for predicting survival and show that it is a determinant of benefit from adjuvant therapy after neoadjuvant treatment. By combining multiple clinically available predictive biomarkers, it may be able to mitigate the drawbacks of using a single biomarker for this important treatment decision.

**Intratumoral nerves drive immune suppression in pancreatic ductal adenocarcinoma via the recruitment of myeloid-derived suppressor cells**
ID: 203

**Kategorie: SCF - Oncology**

Kaan Cifcibasi[1], Carmen Mota Reyes[1], Theresa Zwick[1], Ibrahim Halil Gurcinar[1], Alexander Muckenhuber[2], Katja Steiger[2], Wilko Weichert[2], Helmut Friess[1], Rouzanna Istvanffy[1], Guralp Onur Ceyhan[1], Ihsan Ekin Demir[1]

[1]*Klinik und Poliklinik für Chirurgie, Klinikum rechts der Isar, Technische Universität München, Germany, München*, [2]*Institut für Pathologie, Klinikum rechts der Isar, Technische Universität München, Germany, München*

**Background:**
Pancreatic ductal adenocarcinoma (PDAC) is characterized by pronounced intratumoral nerve growth, neural invasion, and neuroinflammation. Moreover, an immunosuppressive tumor microenvironment is a driving factor in PDAC aggressiveness. In this study, we uncovered a novel crosstalk between the nervous and the immune system in PDAC carcinogenesis.

**Materials and methods:**
Spatial transcriptomic technology was utilized to profile intratumoral nerves of human PDAC and pancreas-innervating sensory neurons in the dorsal root ganglia (DRGs) of genetically engineered mouse models of PDAC. Bulk RNA sequencing of human PDAC specimens and murine DRGs confirmed the spatial transcriptomic analysis. Double immunofluorescence stainings were used to quantify myeloid-derived suppressor cells (MDSCs) in the perineural niche of mouse and human PDAC. In vitro migration assays and secretome arrays were performed for the functional characterization of MDSCs. MDSC depletion in TPAC mice was used to investigate the neurotrophic role of MDSCs in vivo.

**Results:**
Spatial transcriptomics revealed a significant upregulation of the potent MDSC recruiting chemokine CXCL8 specifically in nerves invaded by cancer cells in human PDAC. Moreover, CXCL2 and CXCL15, the mouse homologs of human CXCL8, were upregulated in pancreas-innervating DRGs of tumor-bearing mice compared to their age-matched, tumor-free controls. Congruently, bulk RNA sequencing revealed an upregulation of immunosuppressive pathways in PDAC mouse DRGs and chemokine signaling in human PDAC samples. Co-culturing DRGs with MDSCs led to increased MDSC proliferation and a significantly higher secretion of several cytokines, including CXCL8. In accordance, a higher grade of neural invasion in human PDAC correlated significantly with a higher whole-tumor and peri-neural MDSC density. Functional analyses revealed that MDSCs enhance the migrative behavior of cancer cells towards DRG neurons. In vivo, MDSC depletion in TPAC mice led to a tendential decrease in tumor nerve density.

**Conclusion:**
Our comprehensive analysis revealed a synergistic crosstalk between intratumoral nerves and MDSCs mediated by CXCL8. In this regard, inhibiting neural CXCL8 stands out as a potential therapeutic approach targeting this nerve-immune crosstalk, thereby helping overcome the immunosuppressive TME of PDAC.

Abstracts – DCK 2025 – 142nd Congress of the German Society of Surgery – Munich, March 26–28 • DOI 10.1515/iss-2025-2005
Innov Surg Sci 2025; 10 (Special Suppl 1): s256–s367

### Impact of Lymphadenectomy in Cirrhotic Patients Undergoing Liver Resection for Hepatocellular Carcinoma

ID: 345

**Kategorie: SCF - Oncology**

Jasmin Zeindler[1 2], Gabriel Fridolin Hess[2], Maximillian Von Heesen[3 4], Simone Muenst[5], Martin Bolli[2 6], Otto Kollmar[1 2 4], Savas Deniz Soysal[7]

[1]*Klinik Chirurgie & Viszeralchirurgie, Kantonsspital Baselland, Liestal, Schweiz*, [2]*Clarunis Universitäres Bauchzentrum Basel Viszeralchirurgie, Basel, Schweiz*, [3]*Göttingen Uniklinik, Viszeralchirurgie, Göttingen, Deutschland*, [4]*UKS - Klinik für Allgemeine Chirurgie, Viszeral-, Gefäß- und Kinderchirurgie, Homburg, Deutschland*, [5]*Universitätspital Basel Pathologie, Basel, Schweiz*, [6]*Allgemein- & Viszeralchirurgie - Luzerner Kantonsspital, Luzern 16, Schweiz*, [7]*Medizinische Fakultät | Universität Basel, Basel, Schweiz*

**Background:**

The occurrence of lymph node metastases (LNM) in hepatocellular carcinoma (HCC) is associated with poor prognosis and therefore, the identification of loco-regional LNM is important for tumour staging purposes and outcome prediction. The rate of positive lymph nodes has been shown to be 9% in HCC without cirrhosis compared to around 7% in HCC with cirrhosis. The S3 guidelines recommend the removal of 3 or more lymph nodes for HCC without cirrhosis. In contrast, in HCC with cirrhosis only a selective removal of suspicious lymph nodes should be performed, and the decision to perform lymphadenectomy should be made individually, taking into account the possible higher morbidity of these patients, even though several studies have shown no increase in complication rates.

**Materials and methods:**

This is a retrospective and prospective multicentre cohort study, including all patients undergoing liver resection for HCC between 2009 and 2020 from 3 specialised centres in Switzerland and Germany. Patients with and without lymphadenectomy have been stratified for the occurrence of cirrhosis and no cirrhosis. Complication and survival rates have been analysed using univariate and multivariate Cox regression models.

**Results:**

298 patients underwent liver resection for HCC during the defined time points. Median follow-up time was 52.76 months. 158/298 (53%) patients were diagnosed with cirrhosis. Non-cirrhotic patients underwent lymphadenectomy significantly more often with 20% compared to 5% in cirrhotic patients ($p<0.001$). Interestingly, complications between cirrhotic and non-cirrhotic patients undergoing lymphadenectomy were not significantly different ($p=0.41$). Additionally, comparison of cirrhotic patients with and without lymphadenectomy showed no significant difference regarding complication rates ($p=0.25$). Overall and recurrence free survival in cirrhotic patients with and without lymphadenectomy was not significantly different (adjusted HR 0.84 (95% CI 0.63-1.25, $p=0.31$) and adjusted HR 0.72 (95% CI 0.54-1.14, $p=0.22$), respectively).

**Conclusion:**

European patients with cirrhosis do not show higher complication rates after lymphadenectomy compared to non-cirrhotic patients as well as compared to cirrhotic patients without lymphadenectomy. Future Guidelines should therefore recommend lymphadenectomy in cirrhotic patients in the same manner as in non-cirrhotic patients.

**The FAS receptor mediates neural invasion in pancreatic cancer**
ID: 366

**Kategorie: SCF - Oncology**

Mara Goetz[1 2], Rüdiger Göß[2], Helmut Friess[2], Ihsan Ekin Demir[2]

[1]*Klinik für Allgemein-, Viszeral- und Thoraxchirurgie , Universitätsklinikum Hamburg-Eppendorf, Hamburg, Deutschland*, [2]*Klinik und Poliklinik für Chirurgie, Klinikum rechts der Isar, Medizinische Fakultät, Technische Universität München, München, Deutschland*

**Background:**
Previous studies have identified an increased expression of the FAS receptor (CD45; FAS) in pancreatic adenocarcinoma (PDAC), a highly aggressive malignancy with poor prognosis. A key feature of PDAC is its high incidence of perineural invasion (PNI), which is also an independent prognostic factor with regard to disease-free survival and overall survival. FAS is a critical component of the extrinsic apoptotic pathway, regulating programmed cell death but obtains also non-apoptotic functions such as cell migration. Preliminary findings suggest that the FAS receptor pathway may be relevant to both cell migration and associated PNI in PDAC patients.

**Materials and methods:**
Transcriptomic analyses were conducted on genetically engineered mouse models (GEMM) displaying either human-like PNI or non-PNI phenotypes. RNA expression of FAS in human and murine neuroinvasive cell lines was measured using quantitative reverse transcription PCR (qRT-PCR), while protein expression was analyzed via western immunoblotting.
Histological sections from PDAC patients with nerve-invading versus non-nerve-invading tumors were stained, using an anti-FAS antibody. Caspase 3 expression, a downstream marker of the FAS pathway, was evaluated in human and murine PDAC cell lines using qRT-PCR and western blot analysis.
Additionally, a 3D migration assay was performed to study the non-apoptotic role of the FAS in cell migration. Dorsal root ganglia (DRG) neurons from neonatal mice were exposed to the invasive human PDAC cell line SU.86.86. FAS expression was suppressed using short interference RNA (siRNA), and a neutralizing antibody was applied to inhibit FAS receptor activity. The migration response to Fas ligand (FasL) stimulation was also studied. CDC42 activation (GTP-bound) was assessed by western immunoblotting to investigate its role as a downstream target of the FAS receptor pathway.

**Results:**
The transcriptomic analysis revealed increased expression of the FAS receptor in GEMM models exhibiting human-like PNI compared to non-PNI models. qRT-PCR and western blot analyses confirmed elevated FAS RNA and protein levels in both human and murine neuroinvasive PDAC cell lines. Histological sections from PDAC patients showed significantly higher FAS receptor expression in nerve-invading tumors compared to non-nerve-invading ones. Caspase 3 was moderately elevated in human and murine PDAC cell lines, but the increase was modest compared to the robust FAS receptor expression, indicating that not all PDAC cells are engaging apoptotic pathways. The 3D migration assay demonstrated that pancreatic cancer cells exhibited a strong affinity for neurons. Suppressing FAS receptor expression with siRNA, or inhibiting it with a neutralizing antibody, resulted in decreased migration of PDAC cells. In contrast, stimulation of the FAS receptor with FasL increased PDAC cell migration. Furthermore, CDC42 was predominantly in its active GTP-bound form following FasL stimulation, confirming the involvement of the FAS receptor in enhancing PDAC cell migration.

**Conclusion:**
These preliminary results suggest that the FAS receptor is highly expressed in neuro-invasive cancer cells and human PDAC tissues and that the FAS receptor contributes to PDAC cell migration towards neurons, a critical aspect of PNI. Taken together, this pathway may represent a novel target for therapeutic intervention to impede PDAC progression by mitigating cell migration and perineural invasion.

### Correlation of intratumoral mast cell quantity with psychosocial distress in patients with pancreatic cancer - the PancStress study

ID: 424

**Kategorie: SCF - Oncology**

Rüdiger Göß[1], Alicia Sitte[1], Tutku Tüfekçi[2], Ilaria Pergolini[1], Paulo Pfitzinger[1], Eloísa Salvo-Romero[3], Carmen Mota Reyes[1], Sergey Tokalov[1], Okan Safak[1], Hendrik Steenfadt[1], Ibrahim Gürcinar[1], Ümmügülsüm Yurteri[1], Miriam Goebel-Stengel[4], Gemma Mazzuoli-Weber[5], Andreas Stengel[4], Mert Erkan[6], Helmut Friess[1], Rouzanna Istvanffy[1], Güralp Ceyhan[6], Elke Demir[1], Ihsan Ekin Demir[1]

[1]*Klinikum rechts der Isar, Technische Universität München, Klinik und Poliklinik für Chirurgie, München, Deutschland*, [2]*Koc University School of Medicine, Department of Surgery, Istanbul, Turkey*, [3]*Vall d'Hebron Hospital Universitari, Laboratory of Neuro-Immuno-Gastroenterology, Barcelona, Spanien*, [4]*Universitätsklinikum Tübingen, Innere Medizin, Tübingen, Deutschland*, [5]*Stiftung Tierärztliche Hochschule Hannover, Instituts für Physiologie und Zellbiologie, Hannover, Deutschland*, [6]*Acibadem Mehmet Ali Aydinlar University, Department of General Surgery, Istanbul, Turkey*

**Background:**
Mast cells are commonly found in pancreatic ductal adenocarcinoma (PDAC), yet their role in the disease remains uncertain. Although mast cells have been associated with depression in several diseases, their connection to PDAC in this context remains unclear. This study explored the correlation between mast cells and psychosocial stress in patients with PDAC.

**Materials and methods:**
Prior to surgery, 40 patients with PDAC (n=29 primary resected, n=11 neoadjuvant treated) completed four questionnaires assessing stress and quality of life. Immunostaining was performed on the resected tumor tissue. Spearman analysis was employed to correlate mast cells with distress and neuropeptides serotonin and beta-endorphin serum and tissue levels.

**Results:**
Patients with PDAC exhibited elevated levels of distress and worry. Lower number of mast cells within the tumor correlated with greater psychological burden. Among primary resected patients, mast cell count moderately correlated with joy and inversely with worries. Following neoadjuvant chemotherapy, strong inverse correlation was observed between anxiety, depression, and mast cell quantity. No correlation was found between mast cells and serotonin or beta-endorphin levels.

**Conclusion:**
In summary, mast cell presence inverse correlates with psychosocial stress, suggesting a link between immune cells and psychological well-being in pancreatic cancer. Targeting mast cells could offer therapeutic avenues for addressing cancer-induced depression and anxiety.

**Epidemiology, treatment algorithms, and pattern of recurrence of pancreatic neuroendocrine tumors – a population-based analysis of 1953 cases from the German Cancer Registry Group**
ID: 454

**Kategorie: SCF - Oncology**

Lennart von Fritsch[1], Jannis Duhn[1], Tobias Keck[1], Louisa Bolm[1], Kim Honselmann[1], Thaer Abdalla[1], Rüdiger Braun[1], Markus Kist[1], Steffen Deichmann[1], Ulrich Friedrich Wellner[1]

[1]*Universitätsklinikum Schleswig-Holstein, Campus Lübeck, Klinik für Chirurgie, Lübeck, Deutschland*

**Background:**
Neuroendocrine neoplasia of the pancreas (pNEN) are rare tumors making up around 2% of all pancreatic neoplasms. They are generally divided into functional and non-functional tumors based on their ability to produce a specific hormone. While the reported frequency distribution of non-functional and functional pNENs varies, it is common ground that functional ones are less frequent and are mostly insulinomas. Even though a steady increase in the incidence has been observed over the past decades in Germany and other countries many surgeons rarely encounter this neoplasia.
The aim of this study was to elucidate the epidemiology, survival rates, recurrence patterns and treatment algorithms of pNEN in Germany using real-world data from the German Cancer Registry Group (GCRG) of the Association of German Tumor Centers (ADT).

**Materials and methods:**
Data were obtained from pooled data of 17 clinical cancer registries in Germany collated by the GCRG and used according to their data use regulations. Data of patients with a functional (ICD O-3 codes 8151/3, 8152/3, and 8153/3) and non-functional (ICD O-3 codes 8013/3, 8150/3, 8240/3, and 8249/3) neuroendocrine tumor of the pancreas diagnosed after 2017 were retrieved. Patients diagnosed with a composite carcinoid, mixed islet cell carcinoma/adenocarcinoma, or large-cell /small-cell neuroendocrine carcinoma (LC-NEC/SC-NEC) were excluded. Differences in the epidemiological and histopathological data of patients with a functional or non-functional pNEN were assessed using a Mann-Whitney-U test or chi-square test as appropriate. Overall survival (OS) or disease-free survival (DFS) were calculated using the Kaplan-Meier method and compared using the log-rank test and are expressed with 95% confidence intervals. Furthermore, we compared the treatment algorithms and the cumulative hazard of a local or distant recurrence of disease using the Nelson–Aalen estimator.

**Results:**
A total of 1953 patients were identified with 97% non-functional pNEN. Median age was 63 years (range 16-95) and 44% were female. Of the functional pNENs, 85% were insulinomas, 10% gastrinomas, and 5% glucagonomas. Non-functional pNENs were larger as expressed by the T-stage ($p=0.046$), tended to have more frequent lymph node involvement as expressed by the N-stage ($p=0.102$) and were more often metastasized at diagnosis (22.5% vs. 11.1%, non-functional vs. functional, $p=0.047$). A higher percentage of patients with a functional pNEN underwent upfront surgery (93.7%) compared to patients with a non-functional carcinoma (81.1%, $p=0.088$). Patients with a functional carcinoma showed a better OS compared to non-functional NENs with an overall 5-year survival rate of 0.73 (0.49 – 1.00) vs. 0.48 (0.41 – 0.51) for functional and non-functional pNENs, respectively ($p<0.01$). The DFS was also improved for patients with a functional pNEN with a 5-year DFS of 0.50 (0.25 – 1.00) vs. 0.29 (0.24 – 0.36) for functional and non-functional pNENs, respectively ($p<0.01$). The most frequent sites of metastasis at diagnosis in the non-functional NENs were the liver (87% of all metastasized cases), distant lymph nodes (17%), and bone (13%) and for functional NENs liver (100%) and distant lymph nodes (14%). The cumulative hazard of a local recurrence after curative intent treatment was comparable with a hazard at 5 years of 0.11 vs. 0.20 for functional and non-functional pNENs, respectively ($p=0.56$). The cumulative hazard for newly diagnosed metastasis was higher for patients with a non-functional pNEN with a hazard at 5 years of 0.66 vs. 0.35 for functional pNENs ($p=0.035$). Most frequent regimens for patients with a non-functional pNEN undergoing chemotherapy were platinbased substances with or without etoposide, 5-fluorouracil with or without streptozocin, and capecitabin with or without temozolomide.

**Conclusion:**
Patients with a functional pNEN are diagnosed with less advanced tumors and less often have metastases at diagnosis compared to patients with a non-functional pNEN. Consequently, they are more often eligible for upfront surgery and have a better OS and DFS. Nevertheless, the rates of local recurrence are comparable, while the hazard for a newly diagnosed metastasis is nearly twice as high for patients with a non-functional pNEN. Our data show that both functional and non-functional pNENs have a good outcome. Nevertheless, non-functional pNENs need close follow-up after curative intent treatment for an early detection of distant metastasis.

**Isolated Tumor Cells in Peritoneal Lavage in Colorectal Carcinoma – Detectability and Prognostic Significance: An Exploratory Study**
ID: 500

**Kategorie: SCF - Oncology**

Christoph Thies[1], Florian Richter[1], Susanne Sebens[2], Reinhild Geisen[3], Thomas Becker[1], Julius Pochhammer[1]

[1]*Klinik für Allgemeine Chirurgie, Viszeral-, Thorax-, Transplantations- und Kinderchirurgie, UKSH Campus Kiel, Kiel, Deutschland*, [2]*Institut für experimentelle Tumorforschung, Christian-Albrechts-Universität Kiel, Kiel, Deutschland*, [3]*SYNENTEC GmbH, Elmshorn, Deutschland*

**Background:**
The prognostic significance of isolated tumor cells in blood has been described for various tumor entities, including colorectal carcinoma. The prognostic value of cell detection in peritoneal lavage has also been shown for various entities, but results for colorectal carcinoma remain controversial. Molecular detection methods have established cytokeratin 20 and others as markers for cell detection in blood samples, but not in peritoneal lavage samples. As well, the semiautomated evaluation is not yet established for these samples. This study aims to test different biomarkers using immunofluorescence microscopy for their ability to detect tumor cells in the peritoneal cavity and to determine their prognostic value in an exploratory study.

**Materials and methods:**
Twenty-three patients with rectal carcinoma who received neoadjuvant radiochemotherapy were included and matched with 23 patients without neoadjuvant treatment based on age, sex and UICC stage. Tumor cells were isolated from peritoneal lavages performed at the beginning of resection. Following antibody-based immunofluorescence staining on cytospins, semi-automated microscopic evaluation was conducted. Besides cytokeratin 20, CEACAM 5, EGFR and pan-CK were tested for staining .

**Results:**
Using anti-CK 20, 89.5% of the cells from the HT-29 tumor cell line (positive control) and 0.1% of the MeT-5A cell line (negative control) were stained. This resulted in a sensitivity of 89.5% and a specificity of 99.9%. The other markers showed less specific values in controls, so anti-CK 20 was used for further investigations. In the neoadjuvant-treated group, 73.9% of patients were positive for cell detection in the peritoneal lavage, while 56.5% were positive in the untreated group ($p=0.22$). Cell detection in the peritoneal lavage did not correlate with overall survival in this study cohort ($p=0.42$). Additionally, no statistically significant correlation was found between the UICC stage at the time of surgery and cell detection in both cohorts.

**Conclusion:**
We identified cytokeratin 20 as a reliable marker for semi-automated immunofluorescence microscopy for detecting tumor cells in peritoneal lavage in colorectal carcinoma. However, no prognostic significance for the presence of disseminated tumor cells in peritoneal lavage was demonstrated in this exploratory study population. The prognostic relevance of detecting disseminated peritoneal tumor cells for disease progression needs to be investigated in further studies with larger sample sizes.

**The Gal-9/VISTA immune checkpoint axis critically regulates M2 polarization of tumor infiltrating macrophages in pancreatic cancer.**
ID: 501

**Kategorie: SCF - Oncology**

Helge Meyer[1], Gaetan Aime Noubissi Nzeteu[1], Maximilian Bockhorn[1 2]

[1]*Carl von Ossietzky Universität Oldenburg, Universitätsklinik für Allgemein- und Viszeralchirurgie, Oldenburg, Deutschland*, [2]*Klinikum Oldenburg AöR, Oldenburg, Deutschland*

**Background:**
Immune checkpoint inhibitors (ICIs), targeting pathways like CTLA-4, PD-1, and PD-L1, have significantly advanced cancer immunotherapy, especially in cancers such as melanoma and lung cancer. However, pancreatic adenocarcinoma (PDAC) remains largely unresponsive to these treatments due to its low immunogenicity, as evidenced by a lack of CD8+ cytotoxic T lymphocytes in the tumor microenvironment (TME). Instead, PDAC tumor microenvironment is typically heavily infiltrated with anti-inflammatory tumor-associated macrophages (TAMs).
The immune checkpoint V-Domain Ig Suppressor of T Cell Activation (VISTA), highly expressed on myeloid cells, is emerging as a key player in this context, with growing evidence suggesting that it may drive immune evasion in PDAC. Despite this, the role of VISTA, particularly in relation to Galectin-9 (Gal-9) and macrophage polarization, remains poorly understood.

**Materials and methods:**
Gene expression data from the Cancer Genome Atlas (TCGA) pancreatic adenocarcinoma cohort was used to perform correlation analysis between Gal-9 and VISTA expression, and to analyze survival of patients with high and low Gal-9 or VISTA expression. Further, monocytes from healthy donors were differentiated into polarized macrophages to assess expression of M1 and M2 specific polarization markers as well as expression and release of Gal-9 and VISTA.

**Results:**
Our findings demonstrate a correlation between Gal-9 and VISTA expression in PDAC patients. High Gal-9 expression, alongside elevated VISTA and M2 polarization marker levels, was associated with significantly shorter overall survival. We further unveiled that matrix metalloproteinases orchestrate the constitutive release of soluble VISTA on human peripheral monocytes. Interestingly, the release of soluble VISTA was notably attenuated upon monocyte exposure to macrophage-stimulating cytokines. Furthermore, we observed a modulation of VISTA expression and release in response to pro- and anti-inflammatory cytokines, which are recognized drivers of macrophage polarization. Intriguingly, stimulation with Gal-9 and VISTA promoted M2 macrophage polarization, favoring the anti-inflammatory, tumor-supportive phenotype.

**Conclusion:**
Our study reveals that VISTA and Gal-9 are closely linked in the PDAC tumor microenvironment, with high levels of both associated with worse overall survival. We found that VISTA release is dynamically regulated by macrophage-polarizing cytokines, with M1 macrophages secreting more soluble VISTA, while M2c macrophages—resembling TAMs—show increased surface expression of VISTA. These findings suggest that VISTA plays a dual role: contributing to peripheral tolerance through its soluble form while promoting an immunosuppressive TME through its expression on TAMs. Targeting the Gal-9/VISTA axis could offer a novel therapeutic approach in PDAC, potentially overcoming the limitations of current immune checkpoint therapies by reducing immune evasion and enhancing anti-tumor immunity.

**In-depth proteomic profiling of ampullary adenocarcinoma (AMPAC) pancreatobiliary and intestinal subtype to guide clinical decision making**
ID: 536

**Kategorie: SCF - Oncology**

Louisa Bolm[1 2], Tilman Werner[3], Bianka Karreman[3], Patrick Bernhard[3], Julieta Medaglia[3], Peter Bronsert[3], Mari Mino-Kenudson[4], Cleopatra Schreiber[5], Martina Nebbia[6], Gennaro Nappo[6], Miljana Vladimirov[7], Alessandro Zerbi[6], Motaz Qadan[4], Keith Lillemoe[4], Cristina R. Ferrone[8], Tobias Keck[5], Ulrich F. Wellner[5], Carlos Fernandez-del Castillo[4], Oliver Schilling[3]

[1]*University Medical Center Schleswig-Holstein, Campus Lübeck, Institute of Pathology, Luebeck, Deutschland*, [2]*Massachusetts General Hospital, Harvard Medical School, Department of Pathology, Boston, Vereinigte Staaten*, [3]*Faculty of Medicine, University Medical Center Freiburg, Institute of Surgical Pathology, Freiburg, Deutschland*, [4]*Massachusetts General Hospital, Harvard Medical School, Department of Surgery, Boston, Vereinigte Staaten*, [5]*University Medical Center Schleswig-Holstein, Campus Lübeck, Department of Surgery, Luebeck, Deutschland*, [6]*IRCCS Humanitas Research Hospital, Rozzano, Pancreatic Surgery Unit, Rozzano, Italien*, [7]*University Medical Center Nuernberg, Department of Surgery, Nuernberg, Deutschland*, [8]*Cedars-Sinai Medical Center, Department of Surgery, Los Angeles, Vereinigte Staaten*

**Background:**
Ampullary adenocarcinoma (AMPAC) are a rare entity of gastrointestinal malignancies arising from the Ampulla of Vater. The two main histopathological subtypes in AMPAC are pancreatobiliary (pb) and intestinal (int). Primary resection is the only potentially curative option and adjuvant therapy may be beneficial in AMPAC subgroups. A profound proteomic characterization is warranted in order to guide clinical decision making and patient selection for adjuvant therapy.

**Materials and methods:**
Patients with resected AMPAC were identified from prospectively maintained databases at four high-volume centers of pancreatic surgery. Tumor-bearing areas including the in situ and the invasive component were marked in formalin-fixed, paraffin-embedded specimens by two pathologists and further processed for the mass spectrometry workflow. The total proteome of processed samples was obtained by semi-quantitative data-independent mass spectrometry employing a timsTOF fleX (Bruker). Mass spectrometric raw data are analyzed with the DIA-NN workflow and the 'in library free mode'. Proteomic profiles of AMPAC were correlated with patients' clinical and prognostic parameters as well as long-term outcomes following adjuvant therapy.

**Results:**
A total of 91 patients from four high-volume centers were included. 57.1% of the patients had a pb subtype while 42.9% were of int differentiation. Pb subtype was associated with more loco-regional tumor infiltration and impaired overall survival. Pb as opposed to int subtype AMPAC could be well-differentiated by their respective proteomic profiles. Furthermore, distinct marker profiles of patients with short- vs. long-term overall survival following adjuvant therapy in AMPAC were identified. Proteomic markers associated with cancer cell motility, proliferation and dedifferentiation were more frequent in pb subtype patients and exclusive to patients with short-term survival after adjuvant therapy.

**Conclusion:**
Profound proteomic profiling of AMPAC helps to elucidate the heterogeneous landscape of AMPAC subtypes. Proteomic profiles of patients with impaired long-term outcomes can help to guide patient selection for adjuvant therapy.

Abstracts – DCK 2025 – 142nd Congress of the German Society of Surgery – Munich, March 26–28 • DOI 10.1515/iss-2025-2005
Innov Surg Sci 2025; 10 (Special Suppl 1): s256–s367

**The roles of Galnt2 and St3gal6 genes in neural invasion in pancreatic cancer**
ID: 541

**Kategorie: SCF - Oncology**

Alper Dogruöz[1], Pavel Stupakov[1], Carmen Mota Reyes[1], Rouzanna Istvanffy[1], Roland Rad[2], Helmut Friess[1], Güralp Ceyhan[3], Ihsan Ekin Demir[1 3]

[1]*Technische Universität München, Klinikum rechts der Isar, Klinik und Poliklinik für Chirurgie, München, Deutschland*, [2]*Technische Universität München, Institute of Molecular Oncology and Functional Genomics, München, Deutschland*, [3]*Acibadem Mehmet Ali Aydinlar University, School of Medicine, Department of General Surgery, Hepatopancreatobiliary Unit, Istanbul, Turkey*

**Background:**
Despite extensive research efforts, the mechanisms underlying neural invasion in PDAC remain elusive. Our previous findings from a forward mutagenesis screen using piggyBac-transposon mice suggested a potential association between neural invasion and glycosylation genes *Galnt2* and *St3gal6* [1]. In this study, we aimed to further investigate this association, providing novel insights into the neural invasion and tumorigenesis of PDAC.

**Materials and methods:**
To evaluate the neuroaffinity of murine PDAC cell lines, we performed a 3D migration assay comparing cells with down-regulated expression of *Galnt2* or *St3gal6* to control cells, all of which were isolated from a piggyBac-transposon-mutagenesis mouse model which is previously described by Rad et al. 2015 [2]. Immunohistochemical staining was conducted on human PDAC specimens to investigate the protein expression of *Galnt2* and *St3gal6* in cancer cells that had invaded nerves. Orthotopic transplantation of PDAC cells with down-regulated expression of GalNT2 or St3Gal6, as well as control cells, was performed on wild-type C57BL/6 mice (n=12).

**Results:**
Our 3D migration assay showed that cells with down-regulated expression of *Galnt2* or *St3gal6* exhibited increased neuro-affinity. Immunohistochemical staining revealed that the protein expression of both *Galnt2* and *St3gal6* was significantly lower in cancer cells exhibiting neural invasion than cancer cells far from nerves (GalNT2: $p=0.025$; St3Gal6: $p=0.0097$). All mice implanted with cells down-regulated for *Galnt2* or *St3gal6* developed tumors, whereas only half of the mice implanted with control cells developed tumors.

**Conclusion:**
Our study suggests that *Galnt2* and *St3gal6* may play a role in the neural invasion and tumorigenesis of PDAC, and that down-regulation of *Galnt2* and *St3gal6* may enhance the neuro-affinity of PDAC cells. Further investigation is required to elucidate the underlying mechanisms and clinical implications of targeting these glycosylation-associated genes in the management of PDAC.

[1] Stupakov, P. (2020). *Identifizierung von Neuroinvasion-fördernden Mutationen im Pankreaskarzinom mittels PiggyBac-Transposon-Mutagenese* [Doctoral dissertation, Technical University of Munich].

[2] Rad, Roland et al. A conditional piggyBac transposition system for genetic screening in mice identifies oncogenic networks in pancreatic cancer. *Nature Genetics* vol. 47,1 (2015): 47-56. doi:10.1038/ng.3164

**snRNAseq reveals the Crucial Role of Tertiary Lymphoid Structures in Driving B Cell Maturation into Plasma Cells in PDAC**
ID: 548

**Kategorie: SCF - Oncology**

Carmen Mota Reyes[1], Julian Friedrich[2], Casper van Eijck[3], Raphael Kfuri[4], Lennard Halle[5], Luise Rupp[6], Jaime Martinez Villareal[7], Alexander Muckenhuber[8], Katja Steiger[8], Lidia Estudillo[7], Bo Kong[9], Christoph Michalski[9], Rouzanna Istvanffy[1], Helmut Friess[10], Marc Schmitz[6], Fabian Theis[5], Nuria Malats[7], Casper Hj van Eijck[11], Ihsan Ekin Demir[10]

[1]*Klinikum rechts der Isar, Klinik und Poliklinik für Chirurgie, München, Deutschland*, [2]*Klinikum rechts der Isar, München, Deutschland*, [3]*Erasmus MC, Roterdam, Niederlande*, [4]*Helmholz Munich, München, Niederlande*, [5]*Helmholz Munich, München, Deutschland*, [6]*Technische Universität Dresden, Institute of Immunology, Dresden, Deutschland*, [7]*Centro Nacional de Investigaciones Oncológicas, Madrid, Spanien*, [8]*Klinikum rechts der Isar, Institut für Pathologie, München, Deutschland*, [9]*Heidelberg University Hospital, Department of Surgery, Heidelberg, Deutschland*, [10]*Klinikum rechts der Isar, Department of Surgery, München, Deutschland*, [11]*Erasmus MC, Roterdam, Deutschland*

**Background:**
In pancreatic ductal adenocarcinoma (PDAC), the prognostic relevance of B cells remains controversial, and only when aggregated within tertiary lymphoid structures (TLS) have they been unanimously associated with extended survival. Our aim is to provide the first transcriptomic characterization of B cell subtypes and gain new insights into the immune-promoting role of TLS in PDAC.

**Materials and methods:**
We generated a comprehensive transcriptomic atlas of tumor-associated B cells in PDAC by integrating single cell technology with spatial transcriptomic approaches. Histological characterization of the B cell immune landscape was performed using a novel multiplexed immunohistochemistry (mIHC) panel in treatment-naïve PDAC patients. In addition, TLS assessment on routine H&E slides from two independent PDAC cohorts, comprising participants from the randomized controlled PREOPANC trial (n=44) and the PanGenEU study (n=66), were subjected to uni- and multivariate prognostic analyses, with associated bulk-RNAseq data utilized for additional validation.

**Results:**
The presence of TLS was linked with an expansion of the B cell compartment and their differentiation into antibody-producing plasma cells. Using single nucleus (sn)RNAseq and spatial mapping, we uncovered that certain differentiated B cell subtypes including germinal center (GC) B cells and plasma cells, were exclusively identified in patients with late-stage TLS harbouring GCs. In addition, CD38+ CD138+ plasma cells were predominantly located forming clusters in stromal regions situated in close proximity to TLS. Accordingly, the expression of antibody production and plasma cell markers was significantly heightened in patients with late-stage TLS, indicative of augmented adaptive immune responses. Clinically, we validated the prognostic significance of GC formation within TLS in two independent PDAC cohorts (HR=0.45, 95%CI 0.27-0.77, P.adj.=0.034). Noteworthy, patients with late-stage TLS in the PREOPANC cohort also exhibited a favorable immune milieu enriched with antibody-presenting cells and B cell subsets, including plasma cells.

**Conclusion:**
Our multiomic analysis unveils compelling evidence of selective B cell maturation and active adaptive immunity in PDAC patients harboring late-stage TLS. From a clinical perspective, TLSs emerge as robust prognosticators of survival in PDAC. Overall, our findings provide novel insights into B cell biology and hold potential to refine immunotherapeutic strategies in this challenging tumor context.

**Innervation at acinar-to-ductal metaplasia stage of pancreatic ductal adenocarcinoma**
ID: 550

**Kategorie: SCF - Oncology**

Alper Dogruöz[1], Min-Chun Chen[2], Linhan Ye[1], Rouzanna Istvanffy[1], Henrik Einwächter[2], Roland Schmid[1], Helmut Friess[1], Güralp Ceyhan[3], Ihsan Ekin Demir[1 3]

[1]*Technische Universität München, Klinikum rechts der Isar, Klinik und Poliklinik für Chirurgie, München, Deutschland*, [2]*Technische Universität München, Klinikum rechts der Isar, Klinik und Poliklinik für Innere Medizin II, München, Deutschland*, [3]*Acibadem Mehmet Ali Aydinlar University, School of Medicine, Department of General Surgery, Hepatopancreatobiliary Unit, Istanbul, Turkey*

**Background:**
Pancreatic ductal adenocarcinoma (PDAC) is a devastating disease that is often diagnosed at advanced stages, limiting treatment options and leading to poor patient outcomes. Early detection and intervention are crucial for improving survival rates, but the pathogenesis of PDAC remains poorly understood. Acinar-to-ductal metaplasia (ADM) is early precursor of PDAC. While it is known that neural alterations occur early in pancreatic carcinogenesis, it is unclear whether this occurs at the ADM stage. The aim of this study is to investigate whether neural alterations are present as early as the ADM stage of pancreatic carcinogenesis.

**Materials and methods:**
Pancreatic tissues were collected from LSL-Kras$^{+/G12D}$; Ptf1a$^{tm1(cre)Hnak}$ (KC) mice model at the age of 9-10 weeks. Tissues were paraformaldehyde-fixed and paraffin-embedded. 2 slides per mouse (n=5) were subjected to immunohistochemical staining using anti-PGP 9.5 and anti-Beta-3 tubulin antibodies for labeling nerves. ADM regions measuring 200x200 micrometers in size were selected. ADM regions containing pancreatic intraepithelial neoplasia(PanIN), blood vessels or Langerhans islets were excluded. Acinar regions without obvious pathology, blood vessels, or Langerhans islets were randomly selected in 200x200 micrometer size as a control. Positive pixel counting for PGP 9.5 and Beta-3 tubulin was conducted on both ADM and acinar regions using ImageJ software. The resulting data was subjected to the Mann-Whitney test for comparative analysis. As preliminary experiment, primary acinar cells were isolated from 4-6 weeks old mice of two genotypes: Mek1dd$^{CA/+}$;Ptf1a$^{tm1(cre)Hnak}$ (CMEK1, ADM-promoting experimental group) and Mek1dd$^{CA}$ (MEK1, phenotypically wild-type control group). These cells were cocultured in transwell systems with dissociated dorsal root ganglia (DRG) isolated from 4-12 day-old wild-type C57BL/6 mice. After 120 hours of cocultures, neuronal projections were labeled with an anti-beta 3 tubulin antibody and visualized using immunofluorescence microscopy. Neuronal projection density was then quantified on ImageJ software.

**Results:**
Quantitative analysis using positive pixel counting for PGP 9.5 and Beta-3 tubulin demonstrated a significantly higher density of positive pixels in ADM regions (0,2315 ± 0,06540) than in acinar regions (0,03282 ± 0,01304)(p<0,0001), indicating that ADM regions have more neurons than random, relatively healthy acinar regions. Preliminary results suggest that ADM-like cells promote axonal regeneration in vitro in neurons derived from murine DRG more than phenotypically wild-type acinar cells. However, the difference was not statistically significant due to the small sample size (n = 3; p=0.0531).

**Conclusion:**
This study provides preliminary evidence suggesting hyperinnervation already starts at ADM stage of PDAC, supporting neural alterations are present at the early progression of pancreatic carcinogenesis. This result highlights the importance of investigating relation between ADM cells and neurons in PDAC development, especially considering the known influence of neural components in tumor progression and metastasis. Further studies are needed to confirm and extend these findings.

### Which Surgical Technique for Liver Resection is Favourable in Patients with Colorectal Liver Metastases?

ID: 555

**Kategorie: SCF - Oncology**

Gabriel Fridolin Hess[1], Jasmin Zeindler[1 2], Maximillian Von Heesen[3 4], Simone Muenst[5], Martin Bolli[1 6], Savas Deniz Soysal[7], Otto Kollmar[1 2 4]

[1]*Clarunis Universitäres Bauchzentrum Basel Viszeralchirurgie, Basel, Schweiz*, [2]*Klinik Chirurgie & Viszeralchirurgie, Kantonsspital Baselland, Liestal, Schweiz*, [3]*Göttingen Uniklinik, Viszeralchirurgie, Göttingen, Deutschland*, [4]*UKS - Klinik für Allgemeine Chirurgie, Viszeral-, Gefäß- und Kinderchirurgie, Homburg, Deutschland*, [5]*Universitätspital Basel Pathologie, Basel, Schweiz*, [6]*Allgemein- & Viszeralchirurgie - Luzerner Kantonsspital, Luzern 16, Schweiz*, [7]*Medizinische Fakultät | Universität Basel, Basel, Schweiz*

**Background:**

The liver is the most common metastatic site after curative treatment for colorectal cancer (CRC). Around 50% of all patients with CRC will develop colorectal liver metastases (CRLM). Liver resection (LR) represents a curative treatment option. Benefits of anatomic (ALR) versus non-anatomic liver resection (NALR) show a lack of consistent evidence. While ALR seems to be associated with a higher postoperative complication rate, NALR could lead to more recurrences. We investigated complication and survival rates of patients with CRLM after both resection types.

**Materials and methods:**

This is a multicentre cohort study using retrospectively and prospectively collected data. All patients undergoing LR for CRLM between 2009 and 2020 from 3 specialised centres in Switzerland and Germany were included. Complications and survival rates after ALR versus NALR were analysed using uni- and multivariate Cox regression models.

**Results:**

512 patients were included. Median follow-up time was 65 months. In 250/512 patients (49%) ALR was performed, while 51% underwent NALR. Severe complications grade III or higher according to the Clavien-Dindo classification were observed more often in the ALR than in the NALR group (20% vs. 12%)). Both, length of ICU and postoperative hospital stay have been longer in the ALR group, with 2 and 11 days respectively, compared to NALR with 1 and 10 days. There was no significant difference for overall survival (OS) and recurrence free survival (RFS) between both groups (for OS adjusted HR of 0.96 (95% CI 0.58 - 1.57; p=0.86) and for RFS adjusted HR 1.06 (95% CI 0.74 - 1.51, p=0.77)). Positive lymph node status was associated with a significantly shorter OS (adjusted HR 3.34, 95% CI 1.65 – 6.78, p < 0.01).

**Conclusion:**

This multicentre study investigating ALR versus NALR for CRLM has shown no significant differences in OS and RFS. However, ALR was associated with a higher incidence of postoperative complications. NALR seems to be the favourable choice for patients with CRLM undergoing LR.

**Brg1 suppress DEN-induced hepatocellular carcinogenesis in mice**
ID: 570

**Kategorie: SCF - Oncology**

Baocai Wang[1], Benedikt Kaufmann[1], Carolin Mogler[2], Mathias Schillmaier[2], Rupert Öllinger[2], Rickmer Braren[2], Roland Rad[2], Roland Schmid[2], Helmut Friess[2], Norbert Hüser[2], Guido von Figura[2], Daniel Hartmann[1]

[1]*Universitätsklinik für Allgemeine, Viszeral- und Transplantationschirurgie, Universitätsklinikum Tübingen, Tübingen, Deutschland*, [2]*Klinikum rechts der Isar, Technical University of Munich, München, Deutschland*

**Background:**
Hepatocellular carcinoma (HCC) is the most common form of primary liver cancer. Recurrent mutations in components of the SWItch/Sucrose Non-Fermentable (SWI/SNF) chromatin remodeling complex have been implicated in the pathogenesis of various malignancies. Brahma-related gene 1 (Brg1), a key subunit of the SWI/SNF complex, plays a crucial role in chromatin remodeling and gene regulation. However, the role of Brg1 in liver tumorigenesis and hepatocarcinogenesis remains insufficiently understood.

**Materials and methods:**
To elucidate the role of Brg1 in liver carcinogenesis, we utilized a diethylnitrosamine (DEN)-induced HCC model in wild-type (Control) and hepatocyte-specific Brg1 knockout (Brg1 KO) mice. Mice were administered intraperitoneal DEN injections, and liver tumor burden was evaluated after 11 months. Molecular analyses were performed at various stages of tumor development.

**Results:**
Brg1 expression was significantly upregulated in tumor tissues compared to adjacent non-tumor tissues in DEN-induced HCC in wild-type mice. Notably, DEN-injected Brg1 KO mice exhibited a substantial increase in liver tumor incidence, tumor multiplicity, and tumor volume compared to DEN-injected Control mice. These findings strongly indicate that Brg1 deficiency promotes liver tumorigenesis. Additionally, survival analysis revealed a significantly reduced lifespan in DEN-injected Brg1 KO mice compared to the Control group ($p < 0.05$). Molecular analyses identified dysregulation of key oncogenic pathways in Brg1 KO mice, underscoring the role of Brg1 in maintaining liver homeostasis and tumor suppression.

**Conclusion:**
Our data demonstrate that Brg1 acts as a tumor suppressor in hepatocytes, and its loss accelerates hepatocarcinogenesis in DEN-induced HCC models. These findings highlight the critical role of Brg1 in chromatin dynamics and liver tumor initiation, suggesting its potential as a therapeutic target in HCC.

### Solitary fibrous tumors of the pleura: a 21-year surgical experience

ID: 571

**Kategorie: SCF - Oncology**

Lena Brendel[1], Laura Klotz[1], Michael Allgäuer[2], Romina Rösch[1], Gerlinde Egerer[3], Peter Reimer[1], Claus Heussel[4], Martin Eichhorn[1], Hauke Winter[1]

[1]*Department of Thoracic Surgery, Throaxklinik, Heidelberg, Deutschland*, [2]*Institut of Pathology, Heidelberg University Hospital, Heidelberg, Deutschland*, [3]*Sarcoma Center, Heidelberg University Hospital, Heidelberg, Deutschland*, [4]*Department of Radiology, Thoraxklinik , Heidelberg, Deutschland*

**Background:**

Solitary fibrous tumors (SFT) are a rare entity of mesenchymal tumors, accounting for <5% of all pleural tumors. They have a variable clinical presentation and histopathological evaluation is required for diagnosis. Surgery is usually the treatment of choice.

**Materials and methods:**

During the retrospective 21-year period between January 2003 and January 2024 we reviewed 131 patients (57.2% female and 42.8% male) who underwent primary resection of SFT. Chest computed tomography (CT) revealed the morphology of a well-demarcated masses with density of soft tissue. Patients were identified and retrospectively analyzed for their demographics, operative details, immunohistochemistry, postoperative course, recurrence rate and outcome.

**Results:**

The mean age of the entire cohort was 63 ± 11.4 years. In most cases, surgical resection was performed by anterolateral thoracotomy (61.8%). Mean tumor size was 8.0 ± 7.4 cm. The visceral pleura was significantly more frequently involved than the parietal pleura (87% vs. 13%). The lower lobe of the left lung was the preferred site of SFTs with 35.9% (n=47). Immunohistochemistry showed expressions of CD34, STAT6, vimentin, BCL2 and CD99. Local recurrence and metastasis occurred in five cases each (7.6 % overall). Overall survival was 189.2 month.

**Conclusion:**

The clinical and radiological presentation of solitary fibrous tumors are non-specific and the diagnosis is rarely made before interventional or surgical biopsy. Histologic and immunohistochemical examination is usually required to confirm the diagnosis. Complete surgical resection is the gold standard and prevents recurrence. Regular follow-up with computed tomography examination is recommended due to potential tumor recurrence.

**AURKA expression is positively correlated with mutations in gastrointenstinal cancers**
ID: 651

**Kategorie: SCF - Oncology**

Chuang Yang[1], Patrick Sven Plum[1], Ines Gockel[2], René Thieme[1]

[1]*Universitätsklinikum Leipzig, Klinik und Poliklinik für Viszeral-, Transplantations-, Thorax- und Gefäßchirurgie, Leipzig, Deutschland*, [2]*Leipzig, Leipzig, Deutschland*

**Background:**
Aurora kinase A (AURKA) is important for proper mitosis and its activation peaks during the transition from G2 to M phase. A dysregulation of AURKA was associated with malignancy. Tumor mutational burden (TMB), which is the number of somatic mutations per megabase and may serve as a predictive biomarker for immunotherapy. However, whether AURKA its prognostic value is rarely investigated.

**Materials and methods:**
Public available databases (The Cancer Genome Atlas (TCGA), Genotype-Tissue Expression (GTEx), and The Human Protein Atlas (THPA)) were investigated to explore the expression of AURKA in different cancers entities. The association between AURKA and clinical characteristics and the tumor microenvironment were analyzed. For a validation of the in-silico findings, AURKA's role in esophageal adenocarcinoma (EAC) cells was investigated.

**Results:**
EAC and gastric cancer showed an increased AURKA expression. High AURKA expressions were associated with a poor prognosis and advanced tumor stages. In-silico analyses had shown an association of AURKA with the cell cycle and arachidonic acid metabolism pathways. Additionally, AURKA expression was correlated with immunoregulatory genes and immune cells. A validation of AURKA's role in EAC (OE33 and OE19) demonstrated that inhibition by siAURKA and Aur-Inhibitors decreased cell viability, lowered the colony formation, prolonged the cell cycle, diminished cell invasion, and reduced spheroids growth.

**Conclusion:**
We could prove the role of AURKA and its prognostic value in various cancers, including EAC. The current data demonstrate that AURKA is sufficient for the most cancer entities and could probably be used as a predictive biomarker for EAC and other tumor entities.

**Current Landscape, Clinical Outcomes, and Prognostic Determinants of Neoadjuvant Therapy in Pancreatic Ductal Adenocarcinoma: A Population-based Analysis from the Association of German Tumor Centers.**
ID: 658

**Kategorie: SCF - Oncology**

Jannis Duhn[1], Thaer Abdalla[1], Lennart von Fritsch[1], Markus Kist[1], Louisa Bolm[1], Kim Honselmann[1], Rüdiger Braun[1], Steffen Deichmann[1], Tobias Keck[1], Ulrich Wellner[1]

[1]*University Medical-Center Schleswig-Holstein, Campus Lübeck, Department of Surgery, Lübeck, Deutschland*

**Background:**
Radical surgical resection is regarded the only curative treatment option for Pancreatic Ductal Adenocarcinoma (PDAC). Neoadjuvant therapy (NAT) has shown to improve outcomes of patients with “borderline-resectable” disease based on the NCCN criteria, therefore, the German S3-guidelines recommended NAT for these patients in 2021. However, in less advanced tumors and resectable disease, the benefit of NAT is a matter of controversial debate.
This study aims to evaluate the current clinical practice, outcomes and prognostic factors for NAT in PDAC patients in Germany.

**Materials and methods:**
Anonymized data was obtained from clinical cancer registries participating in the German Cancer Registry Group of the Association of German Tumor Centers (ADT). Patients diagnosed with PDAC (ICD10: C25.x, ICD-O-3: 8140/3, 8500/3) from 2016 onwards with available information on the treatment course and absence of distant metastases (cM1) at diagnosis were selected to delineate the treatment landscape. For further analysis, patients receiving NAT or upfront surgery with complete information on clinical tumor stage were selected and compared regarding epidemiological and histopathological characteristics using Chi-squared test. Survival-rates were compared using the Kaplan-Meier Method with Log-rank test and prognostic factors were evaluated utilizing multivariable Cox-proportional hazard models.

**Results:**
6887 patients meeting the inclusion criteria were identified. Of these, 15.0 % received NAT, 65.3 % upfront surgery, 19.6 % were treated with systemic or local-ablative therapy and 0.1% received palliative surgery. Within in NAT group, 46.3 % of patients were resected. Of these, 47.2 % received adjuvant therapy. Combined, only 21.8 % of NAT patients completed the therapy as recommended in the guidelines.
For further analysis, 847 patients with NAT and 2353 patients with upfront surgery with available information on clinical tumor stages were selected. Patients receiving NAT were significantly younger (65 vs. 70 years, $p < 0.001$) and had more advanced tumors (cT 3-4: 76.9 vs. 34.7 %; cN+: 49.5 vs. 43.0 %, each $p < 0.001$). In contrast, the performance status (ecog) at diagnosis was comparable between both groups. NAT induced a downstaging of tumors, as after resection (n = 378 vs. 2304), NAT patients had lower T stages, less lymph-node metastases, lower tumor Grading and less lymph and blood vessel invasion (each $p < 0.05$). Interestingly, R0 resection was less often achieved after NAT compared to upfront surgery (65.8 vs. 75.4 %, $p < 0.001$). In addition, NAT patients were less likely to receive adjuvant therapy (48.2 vs. 53.9 %, $p = 0.041$).
In univariable analysis, NAT was associated with worse overall survival (median OS: 16.3 vs. 16.9 months, $p = 0.021$). However, after adjusting for potentially confounding variables (age, sex, cT and cN status, tumor localization) NAT was no longer significantly associated with OS (HR 1.09 (0.98 – 1.22), $p = 0.097$). Patients were further stratified according to clinical UICC stages (I, IIA, IIB, III), showing no differences in the OS following NAT compared to upfront surgery in any group.

In the complete NAT group, age ≥ 65 (HR 1.30, p = 0.002), tumor location in the pancreatic head compared to other locations (HR 1.24, p = 0.011) and no resection after NAT (HR 2.34, p < 0.001) were independently associated with worse OS. After resection, reception of adjuvant therapy was associated with improved OS (HR 0.60, p = 0.008) after adjustment for confounding variables. In contrast, positive resection margins were not associated with OS of patients (HR 1.03, p = 0.9).

**Conclusion:**
This analysis provides comprehensive insights into the current clinical practice and outcomes of NAT for PDAC in Germany. Thereby, NAT fails to improve outcomes of patients irrespective of tumor stages. Resection after NAT and adjuvant therapy after resection significantly improves the outcomes, however, this is achieved only by a minority of patients initially receiving NAT. Interestingly, the real-world therapy completion rates in this analysis are lower compared to results from clinical trials.
Most retrospective studies showing survival benefits after NAT selected resected patients only, thus representing a major selection bias. As shown in this analysis, current NAT practice in Germany does not seem to improve outcomes of patients. Therefore, careful selection of patients eligible for NAT is crucial and must be further addressed in prospectively randomized trials.

Abstracts – DCK 2025 – 142nd Congress of the German Society of Surgery – Munich, March 26–28 • DOI 10.1515/iss-2025-2005
Innov Surg Sci 2025; 10 (Special Suppl 1): s256–s367

### Bile stenting and histopathology influence bile microbiome– Insights into a large single-center data base for pancreatic resections

ID: 674

**Kategorie: SCF - Oncology**

Patricia Wyzlic[1], Naita Wirsik[1], Karl Knipper[1], Nikolai Schleussner[1], Jin-On Jung[1], Alexander Damanakis[1], Hans Fuchs[1], Christiane J. Bruns[1], Thomas Schmidt[1]

[1]*Uniklinikum Köln, Klinik und Poliklinik für Allgemein-, Viszeral-, Thorax- und Transplantationschirurgie, Köln, Deutschland*

**Background:**
Pancreaticoduodenectomies are one of the most frequent pancreatic resections for benign and malign tumors. Former studies indicate the potential short-term and long-term consequences of the composition of the bile microbiome. Firstly, certain bile bacteria are associated with significant postoperative complications like surgical site infection and pancreatic fistulas. Secondly, gammaproteobacteria -including Enterobacter-, Escherischia-, Klebsiella-, Pseudomonas- species and many others - have the ability to convert gemcitabine into an inactive pharmaceutic due to a bacterial enzyme called cytidine deaminase. Subsequently, the prevalence and composition as well as the consequences of the bile microbiome need to be explored thoroughly

**Materials and methods:**
The presented data are derived from the pancreatic surgery data base of the university hospital of Cologne, Germany. All patients have agreed to submit their anonymized data for research. 410 patients were included who underwent pancreaticoduodenectomies performed between 2016 and June 2024.

**Results:**
54.4 % of all analyzed patients were male and 44.6% were female. Of all 410 patients, 86.1% underwent pylorus-preserving pancraticoduodenectomy (PPPD), and 13.9% patients underwent pylorus-resecting pancreatidoduodenectomy (PRPD).

50 (12.2%) of all participating patients received a bile duct stenting due to cholestasis before pancreaticoduodenectomy.

A microbial bile duct analysis was documented in 296 cases (72.2%). In 156 out of 296 patients (52.7%), no bacterial growth was observed. In 140 patients (47.3%), bacterial growth was documented in the bile ducts. In these cases, regularly more than one bacterium was identified (90.0%). Overall, more than 20 different bacteria could be identified in this cohort with subsequent different resistograms. The six most frequent bacteria which were identified were: Enterococcus faecalis (n=63), Escherischia coli (n=41), Klebsiella-species (n=41), Enterococcus faecium (n=39), Streptococcus-species (n=34) and Enterobacter-species (n=28). Bacterial growth was significantly more frequent in patients with bile duct stents with 39 of all 50 stented patients (78.0%) ($p<0.001$). In all 39 stented patients with bile positive for bacteria more than one bacterium was registered.

A malignant histopathology was reported in 328 of 410 cases (80.0%). Benign lesions accounted for the 82 other tumors (20.0%), mainly cystic lesions. From all malign neoplasms with registered bile swab (n=238), in 48.3% (n=115) swabs no bacteria were found and 51.7% (n=123) swabs were positive for bacteria. More specifically, out of all PDACs with information on bile microbiome (n=143), 47.6% (n=68) were associated with no bacterial growth and 52.4% (n=75) were positive for bacteria in bile. Out of all benign tumors with information on bile microbiome (n=55), in 70.9% (n=39) patients no bacteria was detected and in 29.1% (n=16) patients bile swab was positive for bacteria. Thus, significantly more patients with malignant neoplasms - respectively PDACs - were positive for bile bacteria than patients with benign neoplasms (p=0.002, respectively p=0.003 ).

Association of bile duct bacteriome with postoperative complications, hospital stay and survival are currently ongoing.

**Conclusion:**
The microbial bile profile of each patient is highly individual if culture is positive for bacteria. In our cohort, significantly more patients who received a preoperative bile duct stent showed a bile microbiome compared to patients who lacked a bile stent. Moreover, patients who suffered from a PDAC diagnosis had significantly more often positive bile swabs than those with a benign diagnosis. This could affect future perioperative antibiotics management.
The effect and power of peri- and postoperative antibiotic prophylaxis respectively therapy will be further explored in this context as well as the relevance of the bile microbiome on complications and long-term survival.

### Exploring pro-tumorigenic effects of malignant ascites on gastric cancer: an *in vitro* study

ID: 676

**Kategorie: SCF - Oncology**

Arianna Castagna[1], Andreas Brandl[1], Frank-Juergen Weinreich[2]

[1]*Universitätsklinikum Heidelberg, Allgemein-, Viszeral- und Transplantationschirurgie, Heidelberg, Deutschland*, [2]*Universitätsklinikum Tübingen, Allgemein-, Viszeral- und Transplantationschirurgie, Tübingen, Deutschland*

**Background:**

The progression of gastric cancer is frequently accompanied by the development of peritoneal metastasis and malignant ascites, which contribute to a poor prognosis. A deeper comprehension of the impact of ascites on tumour cell proliferation, along with a better understanding of the role of growth factors in ascites, can facilitate the development of highly personalised treatment strategies. The objective of this study is to evaluate the proliferation of gastric cancer cell lines (MKN and 23132/87) in response to ascites, in comparison to standard culture conditions.

**Materials and methods:**

An in vitro experimental approach was used to study gastric cancer cell proliferation related to the presence of malignant ascites. The presence of growth factors in ascites was examined. Subsequently, gastric cancer cell lines were cultured under three conditions: medium supplemented with ascites, serum-free medium, and standard medium with 5% FBS. The impact of ascites on cell proliferation was evaluated by monitoring cell growth. To evaluate any potential dose-dependent effects on cell proliferation, a series of ascites concentrations were tested with Real-time cell analysis (RTCA) technology.

**Results:**

The analysis of malignant ascites revealed the presence of several growth factors that could contribute to tumour cell promotion. Proliferation studies showed that both MKN and 23132/87 cell lines exhibited significantly enhanced growth when cultured in medium supplemented with ascites compared to standard conditions. The results demonstrated a clear correlation between ascites concentration and cell proliferation, with higher concentrations leading to increased growth rates in both cell lines.

**Conclusion:**

The presence of malignant ascites has been observed to significantly enhance the proliferation of gastric cancer cell lines, likely due to the presence of growth factors within the ascites. The observed dose-dependent increase in cell growth indicates that higher concentrations of ascites are particularly active in promoting tumour cell proliferation. Further investigations on the interaction of ascites with the tumour microenvironment may facilitate the identification of novel therapeutic targets.

### Navigating the Tumor Landscape: Tumor-Associated Macrophages Drive Pro-Tumorous Microenvironment through CCL17-CCR4 and CCL2-CCR2 Axes Mediated Monocyte Recruitment in Pancreatic Cancer

ID: 692

**Kategorie: SCF - Oncology**

Aydar Khabipov[1 1], Maik Lenz[1], Florian Novak[1], Sander Bekeschus[2], Stephan Kersting[1], Lars Ivo Partecke[1]

[1]*Universitätsmedizin Greifswald , Klinik und Poliklinik für Allgemeine Chirurgie, Viszeral- , Gefäß-, und Thoraxchirurgie, Greifswald, Deutschland*, [2]*Universitätsmedizin Rostock, Klinik und Poliklinik für Dermatologie, Rostock , Deutschland*

**Background:**
Pancreatic cancer (PC) remains one of the most lethal malignancies, characterized by a desmoplastic stroma and an immunosuppressive microenvironment. Tumor-associated macrophages (TAMs) representing the macrophage M2-Type have emerged as critical players in promoting tumor progression, yet the mechanisms underlying their recruitment and differentiation in pancreatic cancer are poorly understood. This study aims to elucidate the role of TAM in the recruitment of further macrophages, which get educated to pro tumorous M2-macrophages, focusing on the chemokine axes involving CCL2-CCR2 and CCL17-CCR4.

**Materials and methods:**
Murine RAW264.7 macrophages were co-cultured with pancreatic cancer cell lines (PDA6606/Panc02) to assess M2 differentiation through flow cytometry. Cytokine levels, including MCP-1, CCL17, and CCL22—ligands for CCR2 and CCR4—were measured in the co-culture. Expression levels of CCR2 and CCR4 in macrophages were assessed following exposure to cancer cells. For migration analysis, we employed a transwell assay and introduced a novel method using a modified ibidi chamber assay to evaluate the recruitment of naïve macrophages to macrophage-tumor co-cultures or PC-educated macrophages compared to PC-mono-cultures or naive macrophage mono cultures.

**Results:**
Co-culture with pancreatic cancer cells significantly induced M2 differentiation in RAW264.7 macrophages and promoted chemocine receptor expression of CCR2 and CCR4 in macrophages. Interestingly elevated levels of their ligands, the recruitment cytokines MCP-1, CCL17, and CCL22 were detected. Migration assays revealed increased recruitment of naïve macrophages to co-cultures and PC-educated macrophage cultures, indicating that the presence of both tumor and macrophages fosters a synergistic effect. This recruitment led to further M2 polarization of naïve macrophages, establishing a pro-tumorous microenvironment through a positive feedback loop.

**Conclusion:**
Our findings highlight a critical mechanism by which TAM orchestrate the recruitment of further TAMs by inducing CCR4- and CCR2- receptors and producing their ligands CCL2, CCL17 and CCL22. This "snowball effect" contributes to the establishment of a pro-tumor microenvironment, presenting potential therapeutic targets in disrupting this interaction to improve patient outcomes in pancreatic cancer.

Abstracts – DCK 2025 – 142nd Congress of the German Society of Surgery – Munich, March 26–28 • DOI 10.1515/iss-2025-2005
Innov Surg Sci 2025; 10 (Special Suppl 1): s256–s367

**The lncRNA within small extracellular vesicles promotes the neuron-cancer crosstalk in pancreatic cancer**
ID: 718

**Kategorie: SCF - Oncology**

Linhan Ye[1], Rouzanna Istvanffy[1], Helmut Friess[1], Ihsan Ekin Demir[1]

[1]*Klinikum rechts der Isar, Department of Surgery, Munich, Deutschland*

**Background:**
Pancreatic cancer (PCa), specifically pancreatic ductal adenocarcinoma (PDAC), is one of the most aggressive cancers with limited treatment options and poor prognosis. Increasing evidence suggests that interactions between PCa and nerves, termed cancer-nerve crosstalk, significantly contribute to tumor growth and progression. Tumor innervation is associated with poorer prognosis and increased recurrence in PCa, and neural invasion is found in nearly 100% of cases. Small extracellular vesicles (sEVs), extracellular vesicles 30-200 nm in size, play a critical role in intercellular communication, including between cancer cells and the nervous system. However, the molecular mechanisms driving cancer-nerve communication remain poorly understood, and this study aims to investigate the role of sEVs in PCa-neuron interactions, focusing on the role of long non-coding RNAs (lncRNAs).

**Materials and methods:**
Using differential ultracentrifugation, sEVs were isolated from both human and murine PCa cell lines and dorsal root ganglia (DRG) neurons. Characterization of the sEVs was conducted via electron microscopy, nanoparticle tracking analysis, and western blot. sEV uptake experiments were performed by labeling the sEVs with PKH67 dye and co-culturing them with neurons and cancer cells. Transcriptome analysis of lncRNAs within sEVs was also conducted. sEVs from a genetically engineered mouse model (TPAC) with high neural invasion were utilized to explore cancer-nerve interactions.

**Results:**
The study demonstrated the reciprocal uptake of sEVs between PCa cells and DRG neurons. sEVs from PCa cells significantly promoted neurite outgrowth in DRG neurons, suggesting their role in cancer-mediated neuritogenesis. Transcriptome analysis revealed differential LncRNA expression between neuroinvasive (TPAC) and non-neuroinvasive (KPC) PCa cells. Notably, Lnc473 was highly expressed in TPAC-derived sEVs and is linked to the cAMP/CREB signaling pathway, which is crucial for neurite formation. Additionally, sEVs from DRG neurons were found to deliver the lncRNA Snhg8 into PCa cells, where it interacted with the Fragile X mental retardation protein (FMRP), a key regulator in tumor immunity. Silencing Snhg8 led to reduced FMRP protein levels, suggesting that Snhg8 stabilizes FMRP protein, although no significant effect on FMRP mRNA was observed. **Conclusion:** This study highlights the critical role of sEVs in mediating PCa-neuron communication, particularly through the transport of LncRNAs such as Lnc473 and Snhg8. These findings suggest that sEV-mediated lncRNA transfer could be a novel mechanism promoting cancer innervation and neural invasion in PCa, offering potential therapeutic targets.

### Impact of Surgical Resection on Patients with Recurrent Perihilar Cholangiocarcinoma – A Retrospective Cohort Study

ID: 763

**Kategorie: SCF - Oncology**

Jens Rolinger[1], Ivan Capobianco[1], Alfred Königsrainer[1], Silvio Nadalin[1]

[1]*Universitätsklinik für Allgemeine, Viszeral- und Transplantationschirurgie, Universitätsklinikum Tübingen, Tübingen, Deutschland*

**Background:**

Perihilar cholangiocarcinoma (PHC) is the most common subtype of cholangiocarcinoma and presents a significant clinical challenge. Achieving complete tumor resection (R0) is crucial for improving patient outcomes. However, recurrence occurs in over half of cases. Data on recurrence patterns are limited, and most existing studies primarily focus on Asian populations. The optimal management strategy for patients with limited recurrent disease remains uncertain. Although promising preliminary results have been reported for intrahepatic cholangiocarcinoma, data on surgical management of recurrent PHC are sparse. This study aims to investigate recurrence patterns and evaluate the outcomes of patients who underwent surgery for recurrent PHC at our institution.

**Materials and methods:**

This retrospective, single-center cohort study included all consecutive patients at Tübingen University Hospital who underwent surgical resection with curative intent for suspected PHC between 2006 and 2023. The analysis focused particularly on recurrence rates in resected patients, the localization of recurrences, as well as the type of subsequent therapy. Additionally, overall survival (OS) was evaluated based on the treatment modalities, comparing surgical versus non-surgical management of recurrent disease. Survival analysis was conducted using Kaplan-Meier models.

**Results:**

The cohort included 271 patients, of whom 197 (72.7 %) underwent surgical resection. The cohort comprised 123 males (62.4 %) and 74 females (37.6 %), with a median age of 66 years (range 27 – 83 years). Bismuth type IV was the most common PHC subtype (54/197, 27.4 %), followed by Bismuth type IIIa (48/197, 24.4 %) and IIIb (48/197, 24.4 %). The median follow-up period was 39.4 months. Recurrence occurred in 108 (54.8 %) patients, presenting as local recurrence or distant metastases. The median recurrence-free survival (RFS) was 15.0 months (95 % CI [10.2; 19.8]), with a 5-year RFS rate of 20.3 %. The most frequent recurrence sites were local (46.3 %), peritoneal (34.3 %), and hepatic metastases (25.0 %). Thirteen patients (12.0 %) with recurrent disease underwent surgery, while 61 patients (56.5 %) received alternative treatments, including chemotherapy, radiotherapy, or best supportive care. The median overall survival (OS) for patients who underwent surgical resection for confirmed recurrence was 94.3 months (95 % CI [31.1; 157.6]), with a 5-year OS rate of 51.3%, calculated from the initial resection. Patients undergoing surgery for recurrent PHC had no significant difference in OS compared to those without recurrence ($p = 0.608$).

**Conclusion:**

Despite the small sample size, subgroup heterogeneity, and potential confounders, surgical resection of recurrent PHC appears to be a viable option, and re-exploration should be considered when resection is technically feasible. Current international guidelines offer inconsistent recommendations for managing recurrent PHC, emphasizing the need for individualized treatment plans developed by a multidisciplinary expert panel at experienced centers. Further studies are necessary to address this issue.

**Prediction of the risk for esophageal cancer development by four insulin resistance surrogates: A population cohort study using the UK Biobank**
ID: 803

**Kategorie: SCF - Oncology**

Chuang Yang[1], Wenke Cheng[1], Patrick Sven Plum[1], Jeanette Köppe[2], Ines Gockel[3], René Thieme[1] [1]

[1]*Universitätsklinikum Leipzig, Leipzig, Deutschland*, [2]*Universität Münster, Münster, Deutschland*, [3]*Leipzig, Leipzig, Deutschland*

**Background:**
Insulin resistance might be a surrogate to predict gastrointestinal malignancies. Among those, esophageal cancer is from special interest, as its incidence was rising during the last decades, especially in western world countries. Therefore, this study explored the association between triglyceride-glucose (TyG), TyG index with body mass index (TyG-BMI), triglyceride/high-density lipoprotein cholesterol ratio (TG/HDL-C), metabolic score for insulin resistance (IR) (METS-IR) and the risk of esophageal cancer.

**Materials and methods:**
The United Kingdom Biobank contains data of over 500,000 participants. For this study, a total of 388,900 participants from 2006 to 2010 were included. Fine-Gray models, restricted cubic spline (RCS), and receiver operating characteristic (ROC) curves were used to assess the association between the four IR surrogates and the risk of esophageal cancer, specifically, esophageal adenocarcinoma (EAC) and esophageal squamous cell carcinoma (ESCC).

**Results:**
Ten years after recruitment, 0.16% (95%CI 0.11 – 0.26%) UKB participants developed esophageal cancer and 4.17% (95%CI 3.86 – 4.46%) are deceased. For each standard deviation increase in the TyG index, TyG-BMI, TG/HDL-C, and METS-IR, the risk of EAC increased by Hazard ratios (HR)1.16, 1.37, 1.08, and 1.36, respectively (all $P < 0.05$), while the risk of ESCC decreased by HRs 0.80, 0.67, 0.77, and 0.65, respectively. RCS analysis revealed that most associations were nonlinear ($P < 0.05$). The most robust diagnostic efficacy has been shown for METS-IR by ROC curves.

**Conclusion:**
The four IR surrogates, TyG index, TyG-BMI, TG/HDL-C, and METS-IR were positive associated with the risk of EAC, while negatively associated with ESCC. Additionally, METS-IR surpassed the other three IR indices in predicting and diagnosing the risks of EAC and ESCC. The METS-IR might be used as an effective metric for identifying populations at risk of esophageal cancer at earlier time points and to improve risk stratification.

Abstracts – DCK 2025 – 142nd Congress of the German Society of Surgery – Munich, March 26–28 • DOI 10.1515/iss-2025-2005
Innov Surg Sci 2025; 10 (Special Suppl 1): s256–s367

### Vision Transformer network discovers the prognostic value of histopathology sections suitable to improve postoperative assurance

ID: 872

**Kategorie: SCF - Oncology**

Wenjie Shi[1], Roland S. Croner[1], Ulf Kahlert[1]

[1]*University Clinic for General-, Visceral-, Vascular- and Trans-Plantation Surgery, Medical Faculty University Hospital Magdeburg, Otto-von-Guericke University, Magdeburg, Deutschland*

**Background:**
Histopathology staining of the resected tissue and signal evaluation by board certified pathologists represent one of the required and cardinal source for disease diagnostics across cancer entities. Pancreatic cancers, a class of tumors comprising cancers with generally clinical unfavorable diagnostics, has been classified in tumor subtypes using molecular-based stratification, medical imaging-based stratification, patient-parameter based stratification and/or based on histopathological features of the tumor material. Low risk pancreatic cancer patients may benefit from adjusted treatment plans in terms of survival probabilities. Informing patients on risk status may also help to optimize life management of newly diagnosed individuals and related caregivers. Our aim was to develop a computational- based algorithm capable of predicting survival outcomes based on features cellular and extracellular properties of pancreatic cancer retrieved from histopathology slides.

**Materials and methods:**
We obtained pathology slides from 173 pancreatic cancer patients and 11 IPMN patients through public databases and real world data from n=2 pathology centers spanning Asian and Western World patients. Follow-up data for cancer patients were retrieved from the database. Each image was uniformly cropped and subjected to quality control. We pre-trained a model based on the Vision Transformer (ViT) architecture, incorporating spatial attention mechanisms and stacked attention weights to construct a deep learning model capable of predicting patient survival. The primary outcomes were overall survival (OS) and disease-free survival (DFS), and the concordance index (C-index) was used to evaluate the model's predictive performance. The survival-related signature calculated by the model was used for patient risk stratification, and survival differences between stratified groups were tested using the log-rank test.

**Results:**
After quality control, a total of n=125 pancreatic cancer slides (from 173 patients) and n=70 IPMN slides (from 11 patients) were included in the final analysis. We trained a ViT model capable of predicting survival outcomes from pancreatic cancer pathology slides. In the validation set, the C-index for OS was 0.79, and for DFS, it was 0.82. The risk score signature derived from the model successfully stratified patients into risk groups, with high-risk patients showing significantly worse prognosis compared to low-risk patients, for both OS and DFS ($p < 0.05$). Our technological advantage to other published histopathological-based stratification approaches due to high ViT model-associated accuracy.

**Conclusion:**
We developed an optimized ViT model capable of predicting survival outcomes for pancreatic cancer patients based on pathology slides. This model has important clinical value for guiding patient stratification and facilitating precision medicine thereby improving postoperative assurance. Our technology may be applicable to tissue sections from biopsies possible innovating pre-operative therapy and surgery planning.

**Evaluation of electrochemotherapy with bleomycin, oxaliplatin, and oxaliplatin with bevacizumab in the treatment of colorectal hepatic metastases in a rat model.**
ID: 878

**Kategorie: SCF - Oncology**

Antonios Spiliotis[1], Gereon Gäbelein[2], Sebastian Holländer[2], Matthias W. Laschke[3], Michael Menger[3], Matthias Glanemann[2]

[1]*Charité Campus Virchow-Klinikum, Chirurgische Klinik, Campus Virchow und Mitte, Berlin, Deutschland*, [2]*Universitätsklinikum des Saarlandes, Klinik für Allgemeine-, Viszeral-, Gefäß- und Kinderchirurgie, Homburg, Deutschland*, [3]*Institut für Experimentelle Chirurgie, Universitätsklinikum des Saarlandes, Homburg, Deutschland*

**Background:**
Several ablative methods have been developed for the treatment of hepatic cancer in patients with unresectable cancer, as a bridging therapy to transplantation or in selected patients as combination with surgical resection. Recent studies and guidelines recommend radiofrequency ablation (RFA) and microwave ablation (MWA) as the ablative procedures with the highest efficacy in the treatment of liver cancer. However, RFA and MWA are prone to heat-sink effect and are not indicated for the treatment of hepatic lesions adjacent to blood vessels or intrahepatic bile ducts. Furthermore, MWA and RFA are associated with thermal-related complications, such as bowel perforation, portal vein thrombosis, cholecystitis, and liver abscess.
In contrast to RFA and MWA, electrochemotherapy (ECT) achieves treatment without thermonecrosis. Consequently, high-risk regions, such as hepatic hilum, hepatic vessels, or bile ducts, can be treated with ECT without thermal-induced complications. The principle of ECT is based on the properties of reversible electroporation (rEP) combined with administration of chemotherapeutic agents. Enhanced cellular permeability is achieved with rEP, which allows increased concentration of chemotherapeutic agents to diffuse into cells, inducing cell death mitosis. In ECT, transportation of typically poorly penetrating chemotherapeutic agents into tumor cells is facilitated, increasing their cytotoxicity.
In our previous study, we reported the beneficial outcomes of ECT with bleomycin (BLM) in the treatment of colorectal metastases with 87.2% tumor necrosis. Aim of the current study is to compare the effectiveness of ECT in the treatment of colorectal metastases with BLM, oxaliplatin (OXP), and OXP with bevacizumab (BVZ) in a rat liver metastasis model.

**Materials and methods:**
WAG/Rij rats were randomized into three groups. Eight days after tumor cell implantation, each group received intravenous chemotherapy combined with rEP. Group 1 received BLM, Group 2 OXP, and Group 3 a combination of OXP/BVZ. Ultrasound and photoacoustic imaging were performed before and five days after treatment to assess the oxygen saturation ($SO_2$) and hemoglobin concentration (HbT) in tumor tissue. Treatment response was evaluated with histological and immunohistochemical analyses on day five following treatment. Tumor necrosis was determined as percentage of the whole tumor area in hematoxylin-eosin stained slices. Immunohistological analyses included PCNA (proliferating cell nuclear antigen), Cleaved caspase-3 (cysteine-aspartic proteases), PECAM-1 (platelet-endothelial cell adhesion molecule- 1; CD31), and MPO (anti-myeloperoxidase antibody).

**Results:**
33.7% decrease in $SO_2$ was observed in the treated area of the BLM group ($SO_2$ pretreatment: 80.1 ± 1.8%; $SO_2$ posttreatment: 46.4 ± 4.8%). The OXP/BVZ group also showed reduced tumor $SO_2$, with a decrease of 12.1% ($SO_2$ pretreatment: 76.4 ± 2.4%; $SO_2$ posttreatment: 64.3 ± 2.5%). On the contrary, $SO_2$ levels in the OXP group presented a modest decrease of 4.7% ($SO_2$ pretreatment: 79 ± 1.4%; $SO_2$ posttreatment: 74.3 ± 1.5%). The BLM group demonstrated the lowest HbT levels within the treated tissue, exhibiting a decrease of approximately 12.7%. In comparison, the OXP and OXP/BVZ groups showed reductions in HbT, measured at 2.1% and 3.4%, respectively.

The histological analysis revealed a higher rate of tumor necrosis in the BLM group, reaching 83%. Tumor necrosis was estimated at 11% and 26% in the animals treated with OXP and OXP/BVZ, respectively. The immunohistochemical analysis of tumor cell proliferation revealed up to 50% PCNA-positive cells in both the OXP and OXP/BVZ groups; whereas, proliferated cells were about 30% in the BLM group.
Treatment with BLM did not lead to increased hepatocellular apoptotic cellular death compared to either the OXP or OXP/BVZ groups (BLM: 7.2 ± 0.7 per HPF; OXP: 14.1 ± 1.4 per HPF; OXP/BVZ: 13.3 ± 1.2 per HPF). The immunohistochemical analysis of MPO-positive neutrophilic granulocytes demonstrated a reduced number of inflammatory cells in tumor tissue following BLM treatment compared to the OXP and OXP/BVZ groups (BLM: 7.1 ± 0.6 cells per HPF; OXP: 10.1 ± 1.4 cells per HPF; OXP/BVZ: 11 ± 0.9 cells per HPF).
Immunohistochemical assessment of CD31-positive microvessels demonstrated a significant reduction in microvessel density in the BLM group compared to the OXP group (69.9 ± 11.3 $mm^{-2}$; 95 ± 4.5 $mm^{-2}$, respectively; $p = 0.026$). Treatment with OXP/BVZ also indicated a decrease in CD31-positive blood vessels relative to the OXP group; however, this difference did not reach statistical significance (80.6 ± 4.6 $mm^{-2}$; $p = 0.185$).

**Conclusion:**
According to our study, ECT with BLM presented beneficial oncological outcomes compared to the other groups with necrosis rates up to 83%. ECT was not associated with increased effectiveness when was conducted with OXP or OXP/BVZ as chemotherapeutic agents.

### Neoadjuvant treatment is an independent predictor of ISGPS grade B/C chyle leak

ID: 891

**Kategorie: SCF - Oncology**

Sakher Shraim[1], Carl-Stephan Leonhardt[2], Ulf Hinz[1], Georgios Polychronidis[1], Markus W. Büchler[3], Mohammed Al-Saeedi[1], Susanne Roth[1], Loos Martin[1]

[1]*Department of General, Visceral, and Transplantation Surgery, University of Heidelberg, Heidelberg, Germany, Heidelberg, Deutschland*, [2]*Department of General Surgery, Division of Visceral Surgery, Medical University of Vienna, Vienna, Austria, Wien, Österreich*, [3]*Department of Pancreatic Surgery, Champalimaud Foundation, Lisbon, Portugal*

**Background:**

Chyle leak is a relevant complication following pancreatic surgery, with variable incidence and severity. This study aims to evaluate the incidence of chyle leak and identify the risk factors associated with its development, focusing on the impact of neoadjuvant chemotherapy (NAT) in patients undergoing resection for pancreatic ductal adenocarcinoma (PDAC).

**Materials and methods:**

All patients who underwent resection for PDAC were identified from a prospective institutional database. Clinicopathologic as well as demographic data were extracted. The primary outcome was chyle leak, graded as A, B, or C according to the ISGPS definition. A prespecified subgroup analysis was conducted for grade B/C chyle leaks. Univariable and multivariable logistic regression modelling was performed with variables with a p-value <0.1 in univariable analysis included in the multivariable model. A p-value of <0.05 was considered significant.

**Results:**

A total of 1406 PDAC patients were included in the final study cohort, of which 1093 (77.7%) underwent upfront surgery while 313 (22.3%) received neoadjuvant treatment (NAT). Median age was 66.2 years (IQR 58.6-74.1) and 732 (52.1%) were male. Pancreatic head resections were performed in 721 (51.3%) patients while 318 (22.6 %) underwent distal pancreatectomies and 367 (26.1%) total pancreatectomies. In total, 407 (28.9%) developed a chyle leak after surgery, of which 269 (19.1%) developed grade A, 121 (8.6%) grade B, and 17 (1.2%) grade C. After adjusting for relevant variables, age ≥70 years (OR 0.74, 95% CI 0.57-0.96), ASA 3/4 (OR 1.33, 95% CI 1.03-1.72), and total pancreatectomy (OR 2.42, 95% CI 1.86-3.16) were associated with the development of chyle leak. In subgroup analysis, NAT was the only predictor for grade B/C chyle leak (OR 2.33; 95%-CI 1.60-3.37) while age, type of resection, and N status were not independently associated with grade B/C chyle leak.

**Conclusion:**

The identified predictors can assist in risk-adapted postoperative care strategies after surgery for PDAC. Prospective validation of these findings is necessary.

Abstracts – DCK 2025 – 142nd Congress of the German Society of Surgery – Munich, March 26–28 • DOI 10.1515/iss-2025-2005
Innov Surg Sci 2025; 10 (Special Suppl 1): s256–s367

### Clinical Application of a Sarcoma Classifier Based on DNA Methylation Profiling: A Single-Center Experience

ID: 892

**Kategorie: SCF - Oncology**

Justus Osterloh[1], Alexander Runkel[1], David Braig[1], Steffen Ulrich Eisenhardt[1]

[1]*Uniklinik Freiburg - Klinik für Plastische und Handchirurgie, Freiburg im Breisgau, Deutschland*

**Background:**
Soft tissue sarcomas are rare malignant tumors of mesenchymal origin characterized by their heterogeneity. Currently, diagnosis is challenging and relies primarily on histopathological analysis and molecular markers. Classification of sarcomas using epigenetic analysis offers the possibility of an objective and reliable categorization of sarcoma subtypes. Therefore, a DNA methylation-based sarcoma classifier was recently introduced.

**Materials and methods:**
In this single-center study, we describe our experiences in clinical application of the DNA methylation classification system from the German Cancer Research Center (DKFZ), Heidelberg. 39 samples from 30 patients were examined and compared by histopathology and DNA methylation analysis before and after neoadjuvant therapy.

**Results:**
In 17 (43.6%) samples, a clear match to a reference group could be made using the methylome classifier (calibrated score ≥0.84). The diagnosis was in concordance with the histopathologic diagnosis in 76.5% (13/17). In three cases (17.6%), the classifier suggested a non-malignant or low malignant diagnosis, while the histopathologic diagnosis was consistent with high malignant sarcoma. In 16 (41%) of the analyzed samples, a lower confidence score was reached (calibrated score 0.3 to 0.84). In 6 cases (15.4%), the methylome-based classification system could not be matched to a reference group (calibrated score <0.3).

**Conclusion:**
The classification of sarcomas using epigenetic analysis methods is a valuable adjunctive diagnostic tool to confirm or revise the histopathologic diagnosis. However, in our clinical experience, the concordance rate was low and refinement of the applicability of the classifier is needed to clearly demonstrate its clinical utility.

Abstracts – DCK 2025 – 142nd Congress of the German Society of Surgery – Munich, March 26–28 • DOI 10.1515/iss-2025-2005
Innov Surg Sci 2025; 10 (Special Suppl 1): s256–s367

### GLUT1-Positive Immune Cells at the Tumor Interface: Uncovering a Metabolic Pathway to Enhanced Survival in Colorectal Liver Metastases

ID: 954

**Kategorie: SCF - Oncology**

Stefan M. Brunner[1], Eva Griesshammer[1], Niklas Bogovic[1], Hans Schlitt[1]

[1]*Uniklinik Regensburg, Klinik für Chirurgie, Regensburg, Deutschland*

**Background:**

Glucose transporter 1 (GLUT1) is essential for cellular energy regulation, and elevated GLUT1 expression in tumor cells has been associated with poor prognosis across several malignancies, including colorectal carcinoma. The effect of increased GLUT1 expression in immune cells, however, on their antitumor functionality has not been thoroughly explored. This study investigates the role of GLUT1-positive immune cells within the infiltration margin of colorectal liver metastases on patient survival outcomes. A detailed analysis was performed to differentiate between GLUT1-positive and GLUT1-negative CD8+ TEMRA cells, hypothesized to significantly influence patient prognosis.

**Materials and methods:**

Immunohistochemical staining for GLUT1 was conducted on colorectal liver metastasis tissue samples, and GLUT1 expression was correlated with survival data sourced from a patient database. Additionally, leukocytes isolated from the infiltration margin of resected colorectal liver metastases were differentiated by flow cytometry, and the cytotoxicity of CD8+ TEMRA cells was examined based on GLUT1 expression levels.

**Results:**

Our findings indicate that elevated GLUT1 expression within the infiltration margin correlates significantly with prolonged patient survival ($p=0.011$), independent of the tumor's proliferation rate. This survival benefit was particularly pronounced in a subgroup of patients with solitary liver metastases. Flow cytometric analysis revealed an increased frequency of CD8+ TEMRA cells with a trend towards higher granzyme B expression in GLUT1-positive compared to GLUT1-negative CD8+ TEMRA cells, although statistical significance was not achieved ($p=0.06$).

**Conclusion:**

These results suggest that GLUT1 expression in immune cells at the tumor-host interface may enhance antitumor immune responses, potentially through increased cytotoxic activity of CD8+ TEMRA cells. This study highlights the potential of GLUT1 as a prognostic marker and a target for therapeutic strategies aimed at augmenting immune-mediated tumor suppression in colorectal liver metastases.

**Detection and role of ecDNA at distinct evolutionary stages during pancreatic carcinogenesis.**
ID: 959

**Kategorie: SCF - Oncology**

Dou MA[1], Matthäus Felsenstein[1]

[1]*Chirurgische Klinik, Charité – Universitätsmedizin Berlin, Berlin*

**Background:**
Pancreatic duct adenocarcinoma (PDAC) is an aggressive disease showing high recurrence rates and poor prognosis. The underlying genomic changes have been characterized by many in-depth short-read sequencing efforts and unfolded complex genomic aberrations that accumulate from precursor to later stage disease. Chromothripsis has been shown responsible in many instances and could lead to molecular by-products such as extrachromosomal DNA (ecDNA). Genome wide analyses of TCGA datasets estimated that ecDNA is present in around 15-20% of PDAC patients. The evolutionary trajectory of their presence during distinct disease stages has not yet been deciphered.

**Materials and methods:**
Three-dimensional organoid tissue culture was established from 42 primary tissue samples. Those samples were harvested from either normal pancreatic ducts (n=13), cystic precursors (n=4, IPMN), PDAC (n=23) and metastatic samples (n=4). Phenotype characterization included life- and phase contrast imaging as well as IHC using markers for duct differentiation. Following enrichment of ductal/neoplastic content, we performed shallow whole genome (WGS) and exome sequencing (WES) to identify their gene signatures and complex chromosomal aberrations. Downstream analyes was performed using state-of-the-art computational algorithms (Amplicon Architect). The clinical outcome and follow-up of patients were documented in clinical datasets. Cytotoxicity assays were performed with Gemcitabin to assess oncogenic fitness of ecDNA.

**Results:**
We established and characterized a living biobank of pancreatic organoids that represented distinct stages during the pancreatic carcinogenesis. Life-/Phase-contrast imaging and IHC revealed organotypic distinctions reflecting the individual disease stage. Investigation of the exome revealed a diverse landscape of driver gene alterations that included PDAC specific oncogenes (*KRAS, Myc*) and tumor suppressors (*CDKN2A, TP53, SMAD4*). Amplicon Architect identified complex chromosomal abberations (BNDs, Translocations), while few ecDNA like structures were detected in the genome. Integrating cytotoxicity assays enriched for ecDNA content and may reflect an important resistance mechanism.

**Conclusion:**
Our results suggest that ecDNA plays an incremental role from early to later stage disease. Our study provides a comprehensive overview of ecDNA characterization during pancreatic carcinogenesis. EcDNA may be capable to predict disease recurrence.

Abstracts – DCK 2025 – 142nd Congress of the German Society of Surgery – Munich, March 26–28 • DOI 10.1515/iss-2025-2005
Innov Surg Sci 2025; 10 (Special Suppl 1): s256–s367

### The AP-1 factor JUNB correlates with worse survival of patients with esophageal adenocarcinoma

ID: 968

**Kategorie: SCF - Oncology**

Nikolai Schleussner[1], Karl Knipper[1], Ella Leugner[2], Uraz Yasar[2], Christian Goddemeier[1], Naita Wirsik[1], Jin-On Jung[1], Hans Anton Schlößer[1], Hans Fuchs[1], Wolfgang Schröder[1], Lars Schiffmann[1], Alexander Quaas[3], Christiane J. Bruns[1], Thomas Schmidt[1]

[1]*Uniklinik Köln, Klinik für Allgemein-, Viszeral-, Thorax- und Transplantationschirurgie, Köln, Deutschland*, [2]*Uniklinik Heidelberg, Klinik für Allgemein-, Viszeral- und Transplantationschirurgie, Heidelberg, Deutschland*, [3]*Uniklinik Köln, Institut für Allgemeine Pathologie und Pathologische Anatomie, Köln, Deutschland*

**Background:**
Malignant cells have in contrast to non-transformed cells deregulated transcriptional networks. Transcription factors are essential to initialize, maintain and adjust these networks. Activator Protein-1 (AP-1) transcription factors are expressed in many cancer entities including adenocarcinomas and have been correlated to deregulated transcription and tumor-promoting mechanisms. Esophageal cancer is despite complex treatment approaches still correlated to poor overall survival. There is an urgent need for better patient stratification to increase the outcome of the multimodally treated patients.

**Materials and methods:**
Seven hundred patients with the diagnosis of esophageal adenocarcinoma were included into this study, all underwent surgery with a curative intent in the university hospital cologne between the years 1996 and 2019. Immunohistochemical staining for JUNB and cJUN was performed. Clinicopathological data were prospectively collected and retrospectively reviewed including TNM status, treatment and overall survival. The study population was split into two cohorts with either high or low expression of AP-1 factors.

**Results:**
Patients with a high JUNB expression level correlate to a reduced overall survival (OS) compared to patients with a low expression ($p = 0.006$). Furthermore, in the multivariate analysis high JUNB expression was shown to be an independent risk factor for reduced patient survival (hazard ratio, 1.347, 95% CI, 1.074–1.690; $p = 0.01$). In addition, subgroup analysis demonstrated a significantly reduced OS for high JUNB expression in the subgroup of patient with neoadjuvant treatment ($p = 0.006$).

**Conclusion:**
This study suggests JUNB expression as a novel biomarker to stratify patients with esophageal adenocarcinoma, especially in the subgroup of neoadjuvant treated patients. Identifying those patients and adjusting the treatment might lead to better outcome.

**Adjusted 3D human valvular interstitial cell culture to emulate fibrosis vs. calcification in calcific aortic valve disease**
ID: 100

**Kategorie: SCF - Regeneration and Tissue Engineering**

Claudia Dittfeld[1], Thea Grüneberg[1], Anett Jannasch[1], Klaus Matschke[1], Sems-Malte Tugtekin[1]

[1]*Faculty of Medicine and University Hospital Carl Gustav Carus, Technische Universität Dresden, Department of Cardiac Surgery, Heart Centre Dresden, Dresden, Deutschland*

**Background:**
Calcific aortic valve disease (CAVD) is the most common valvular heart disease. In Germany more than 40.000 patients are treated by surgical replacement of the diseased valve or transcatheter implantation of AV prostheses per year. One quarter of the population older than 65 is assumed to manifest a sclerosis of the aortic valve and 1.8 % suffer from progress to stenosis annually. Still there is no substance-based treatment strategy to prevent disease progression. Reason for lack of valuable drug development is that model systems to reflect CAVD in an experimental setup possess multiple limitations. 2D valvular interstitial or endothelial cell cultures (VIC/VEC) do not simulate the tissue structure and relevant impact of extracellular matrix not to mention the extreme physiological and biomechanical aspects in the system. But also, animal models hardly reflect the human situation due to different physiological parameters and anatomy in small animal models or disease time line in large animal models also hardly developing AV calcification. Researchers still focus on improvement of model systems especially AV tissue culture concepts. The herein presented 3D VIC model bridges the 2D culture and the tissue culture concepts with the ability to use cells of human origin and simulation of different tissue pathologies such as fibrosis or calcification using differing media formulations.
Aim of the study is to provide an easy too handle and reproducible 3D tissue simulating culture system allowing reflection of multiple aspects of CAVD individually to study drug effects and with the possibility to analyze a large number of replicates.

**Materials and methods:**
Valvular interstitial cells were isolated from patient material (Ethical votum: EK429102015) and cultured as conventional monolayer in DMEM (containing 10% FCS). For 3D culture, 12.000 VICs were seeded per well of a low adhesion round bottom plate to form spheroids within four days. 3D cultures were feed every 3-4 days by replacing half of the medium volume and monitored for 28 days. The following conditions were compared: A) control medium DMEM B) DMEM, 1%FCS, 1% ITS (Insulin/ Transferrin/ Selenium) C) DMEM, TGF-ß1 (1 µg/ml) D) DMEM, 50 µM L-ascorbic acid-2-phosphate, 10 nM dexamethasone, 10 mM ß-glycerophosphate; named ADGM. Spheroid size was determined by microscopical monitoring and measurement of diameter at feeding time points. VIC viability was proven by alkaline phosphatase assay at day 4 or 8 and at day 28. LDH viability stain was performed using cryosectioned spheroid samples (DAPI nuclei co-stain). Spheroids were histologically prepared, sliced and sections were stained via HE, Movats Pentachrom, von Kossa and Alizarinred staining to analyze cell nuclei, morphology and calcification status. Immunohistochemistry was used to detect α-smooth muscle actin (αSMA) as a marker for myofibroblast differentiation.

**Results:**
Spheroid formation was proven for all media formulations. Initial spheroid diameters at day 4 to 8 did not differ significantly. At day 28 the ADGM spheroids exhibit with 333.5 µm ± 24.8 µm a significantly smaller diameter than spheroids in all other conditions. VICs in TGF-β1 supplemented DMEM are with a diameter of 506.2 ± 20.6 µm larger than the DMEM control cultures (424.7 ± 33.1 µm) at day 28. ITS supplemented culture conditions revealed reduced sphericity and oval shaped spheroids.
Nevertheless, no significant differences were detected in APH viability assay activity comparing early and late culture time points. Initial LDH viability stain revealed no necrotic core regions but increased blue staining in the outer spheroid layers.

Calcification of the cultures after 28 days was verified by von Kossa and Alizarinred stain in histology. In all ADGM induced 3D culture setups a calcification associated to cellular structures was detected. Half of the individual cell preparations also exhibited calcification in the DMEM control conditions mainly in the inner part of the 3D cultures. In contrast, TGF-β1 supplemented cultures, although largest spheroid size was detected, did not show calcification. Here, areas of higher ECM density without cell nuclei were monitored and a spheroid core, forming tissue nodules. The surrounding fibrous rings are rich in glycosaminoglycans and cells show elongated nuclei and are strongly positive for α-SMA.

**Conclusion:**
Valvular interstitial 3D cell culture model using specific adjustment by differing medium supplementation can discriminate and simulate pathological processes of aortic valve pathology. Using cells from a single human source, aspects like fibrosis or calcification can be investigated independently. Bridging 2D and tissue culture, the standardized in vitro 3D model is envisioned for application in CAVD drug testing procedures.

**Influence of the blood microbiome and bacterial translocation on postoperative liver regeneration**
ID: 304

**Kategorie: SCF - Regeneration and Tissue Engineering**

Jonas Santol[1 2 3], Emma Whittle[4], Laura Godin[5], Markus Ammann[2 6], Laura Brunnthaler[3], Yawen Dong[1 2], Joel Probst[1], Valerie Laferl[7], Anna E. Kern[7], Gregor Ortmayr[8], Alice Assinger[3], Craig Jenne[5], Marina Walther-Antonio[4], Patrick Starlinger[2 9 10]

[1]*Klinik Favoriten - Wiener Gesundheitsverbund, ehemaliges Kaiser Franz Josef Spital, Abteilung für Chirurgie, Wien, Österreich*, [2]*Mayo Clinic, Department of Surgery, Rochester, Vereinigte Staaten*, [3]*Medizinische Universität Wien, Institut für Gefäßbiologie und Thromboseforschung, Wien, Österreich*, [4]*Mayo Clinic, Division of Surgical Research, Rochester, Vereinigte Staaten*, [5]*University of Calgary, Department of Microbiology, Immunology and Infectious Diseases, Calgary, Kanada*, [6]*Landesklinikum Wiener Neustadt, Wiener Neustadt, Österreich*, [7]*Medizinische Universität Wien, Wien, Österreich*, [8]*Medizinische Universität Wien, Zentrum für Krebsforschung, Wien, Österreich*, [9]*Medizinische Universität Wien, Universitätsklinik für Allgemeinchirurgie, Wien, Österreich*, [10]*Medizinische Universität Wien, Zentrum für Physiologie und Pharmakologie, Wien, Österreich*

**Background:**
The human microbiome contributes significantly to the development of chronic liver disease. If the microbiome is dysregulated, bacteria and bacterial endotoxins can cross the intestinal barrier via bacterial translocation (BT) and reach the liver. In previous publications we could document the detrimental effect of an overshooting inflammatory response during liver surgery on postoperative liver regeneration and outcome. This study aimed to investigate a possible mechanism of BT influencing liver regeneration and subsequent development of posthepatectomy liver failure (PHLF).

**Materials and methods:**
192 patients undergoing liver surgery were included from a prospective biorepository. Plasma and serum samples were collected prior to surgery, on the first postoperative day (POD) and intraoperatively from the portal vein prior to portal vein ligation. Lipopolysaccharide (LPS) levels were measured and bacterial load and blood microbiome composition were analyzed using bacterial rRNA sequencing. Wildtype mice were treated with low-dose LPS (0.01 mg/kg) or phosphate buffered saline for ten days before undergoing hepatectomy. Mouse livers were either harvested 2 hours after partial hepatectomy or 48 hours after partial hepatectomy. To investigate whether the effects of LPS treatment on liver regeneration were targetable, a group of mice that underwent partial hepatectomy (48h harvesting timepoint) was treated with a neutrophil extracellular trap (NET) inhibitor, DNase I, immediately after surgery and again at 24 and 48 hours post-surgery. After 2 hours, regenerating livers were stained for platelets, neutrophils, and neutrophil extracellular traps (NETs) and examined via intravital microscopy using a confocal microscope. At 48 hours, the livers were harvested and stained for Ki-67 using immunohistochemistry.

**Results:**
Preop Bacterial load was higher in patients who developed PHLF ($p=0.048$). Patients who developed PHLF also had a higher abundance of gram-negative bacteria in their portal circulation. In patients who developed PHLF LPS, the main outer membrane protein of gram-negative bacteria, increased in the systemic circulation from preop to POD1 ($P=0.033$). Interestingly, in patients who developed PHLF higher LPS levels could even be measured in the hepatic circulation in the portal vein, prior to inflow occlusion ($P=0.038$). LPS in the portal circulation also correlated with myeloperoxidase, a marker for neutrophil activation, on POD1 ($R=0.823$, $p=0.023$). LPS treatment in mice led to higher neutrophil accumulation ($p=0.027$) 2h after hepatectomy (Fig. 1A), which was associated with a tendency for increased formation of NETs. Interestingly, 2h after partial hepatectomy after LPS treatment platelet accumulation was increased as well, especially of larger platelet aggregates (25µm aggregates, $P=0.026$) (Fig. 1B). When examining hepatocyte proliferation 48h after partial hepatectomy LPS

Abstracts – DCK 2025 – 142nd Congress of the German Society of Surgery – Munich, March 26–28 • DOI 10.1515/iss-2025-2005
Innov Surg Sci 2025; 10 (Special Suppl 1): s256–s367

treatment led to decreased Ki-67 positive hepatocytes in mice 48h after hepatectomy (P=0.003) (Fig. 1C). However, mice treated with DNase I following LPS treatment showed improved liver regeneration compared to those treated with LPS alone (P = 0.002), and proliferation comparable to controls (P = 0.1128) (Fig. 1C).

**Conclusion:**
BT seems to prime patients for development of PHLF via translocation of gram-negative bacteria and LPS. This is driven by increased neutrophil accumulation and formation of microthrombi. LPS treatment inhibited liver regeneration in mice. However, these mice could be rescued via NET inhibition. This holds significant therapeutic potential, as NET inhibitors are available for clinical use.

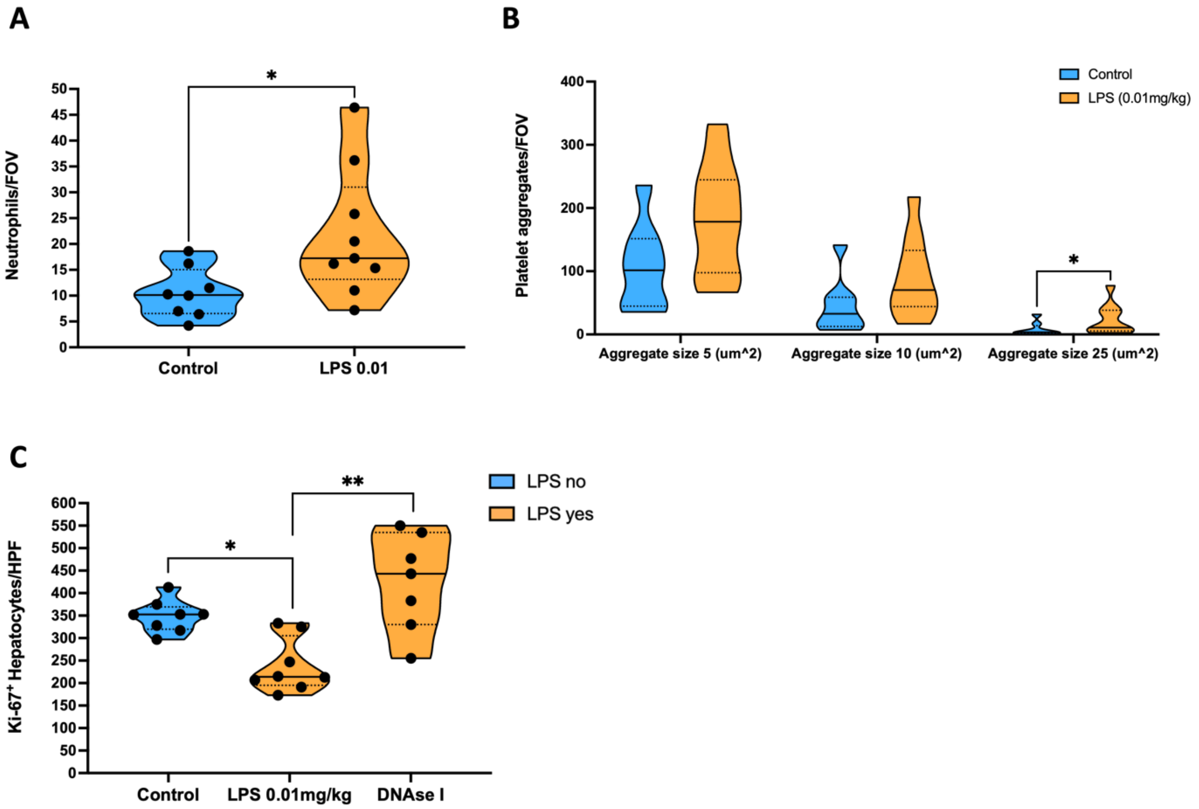

**Fig. 1. Neutrophil and platelet accumulation increases 2h after low-dose LPS treatment inhibiting postoperative liver regeneration.**
(A) Neutrophils per FOF and (B) platelet aggregates per FOV 2h after partial hepatectomy in mice treated with either 0.01 mg/kg LPS or controls treated with phosphate buffered saline. (C) Ki-67 positive Hepatocytes per HPF 48 hours after partial hepatectomy in mice treated with either 0.01 mg/kg LPS, 0.01 mg/kg LPS and DNAse I or controls treated with phosphate buffered saline. FOF=Field of view, HPF=High-power fields, LPS=Lipopolysacharides

### Decellularized Struthio trachea as a tissue engineered neo-aortic graft

ID: 327

**Kategorie: SCF - Regeneration and Tissue Engineering**

Felix Strobl[1], Christian Wintzer[2], Andreas Greiner[1], Leon Bruder[1]

[1]*Charité Campus Benjamin Franklin, Klinik für Gefäßchirurgie, Berlin, Deutschland*, [2]*St. Marienkrankenhaus, Klinik für Gefäßchirurgie, Berlin, Deutschland*

**Background:**

While Dacron prothesis constituted the standard material in open aortic replacement, they lack in their resistance to prothesis infection. Options currently available are limited by their availability (e.g. aortic homografts, autologous vein) or differ heavily from native aorta in their biomechanical properties (bovine pericardial tubes). Decellularized tissue engineered grafts could offer more physiological scaffolds. With its suitable dimensions and exceptional durability, the trachea of the ostrich could constitute a novel solution in this context.

**Materials and methods:**

4 tracheae of Struthio camelus were decellularized using two different protocols. Trachea pieces measured approximately 30 x 150 mm and were decellularized by either Sodium desoxycholate plus DNAse (protocol I) or Triton X-100 plus DNAse and RNAse (protocol II). Decellularization efficacy was measured by DNA quantification using the Nano Drop, as well as through HE and DAPI staining. Cytotoxicity was measured indirectly according to ISO 10993 standards with murine L929-cells. A standard bovine pericardium patch served as control group.

**Results:**

DNA quantification showed prominent reduction in concentration compared to native trachea. Protocol I reduced concentration and by an average of 67.5% ($p = 0.655$), Protocol II by an average of 27.3 % ($p = 0.180$). In histological analysis, almost no cell nuclei were detectable in HE as well as DAPI staining after decellularization. Overall cytotoxicity levels were mild to moderate in the decellularized tracheal graft, while being negligible in the bovine pericardium patch.

**Conclusion:**

Decellularization of large sections of ostrich-trachea seems feasible using multiple protocols. Reduction of cytotoxicity needs to be achieved, possibly by more efficient sterilization of grafts. This publication serves as a prove of concept, further exploration with multiple samples is necessary to determine optimal modalities, as well in-depth analyses of biomechanical and molecular properties of decellularized trachea.

### Electrospun PLLAcoCL/PDLA-scaffold enables recellularization and migration of HUVEC in-vitro

ID: 373

**Kategorie: SCF - Regeneration and Tissue Engineering**

Lorenz Mertens[1], Leon Bruder[2], Andreas Greiner[2], Hanin Alkhamis[3], Katarzyna Polak-Kraśna[3]

[1]*Charité - Universitätsmedizin Berlin, Klinik für Gefäßchirurgie, Berlin, Deutschland*, [2]*Charité - Universitätsmedizin Berlin, Klinik für Gefäßchirurgie, Berlin, Deutschland*, [3]*Helmholtz-Zentrum Hereon, Institut für aktive Polymere, Teltow, Deutschland*

**Background:**
In vascular surgery, often synthetic materials such as PET or PTFE are being used for reconstruction. These materials come with certain limitations, such as high rates of infection and thromboembolic complications due to an insufficient recellularization and hence absence of a remodeling process. New materials are therefore needed to minimize complications following vascular surgery and ensure long term patient safety.

**Materials and methods:**
A biodegradable, hyperelastic copolymer (PLLAcoCL/PLLA) has been developed previously and was further processed into a microporous scaffold-mesh using electrospinning. Cytotoxicity was assessed indirectly according to ISO 10993 standards using murine L292 fibroblasts. For recellularization, human umbilical vein endothelial cells were seeded upon the scaffold and incubated until they formed a confluent layer. A standardized migration assay was then performed to assess cell migration and evaluated using confocal microscopy.

**Results:**
The PLLAcoCL/PDLA polymer showed excellent mechanical stability and elasticity, while only slight/negligible cytotoxicity could be detected. HUVEC adhered fully after only 24 hours of incubation, while migration on the polymers surface could also be detected. The cells beta-aktin network was formed more strongly in the presence of fibronectin the incubation medium.

**Conclusion:**
Recellularization of a novel hyperelastic and biodegradable electrospun polymer seems feasible with the presence of a hydrogel potentially enabling faster adhesion and migration. The polymer presented could therefore help to overcome limitations of prosthetic materials in vascular surgery. Still, further investigation on cell behavior has to be conducted especially in a dynamic culture as well as animal testing.

### Development of advanced *in-vitro* human bronchial epithelial models and tissue engineering in COPD enabled by thoracic surgery translational research

ID: 417

**Kategorie: SCF - Regeneration and Tissue Engineering**

Mircea Gabriel Stoleriu[1 2], Meshal Ansari[2], Maximilian Strunz[2], Andrea Schamberger[2], Motaharehsadat Heydarian[2], Christina Gabriel[2], Aakruti Nayak[2], Anja Disovic[2], Juliane Schneider[2], Michael Gerckens[2 3], Gerald Burgstaller[2], Yaobo Ding[2], Ali Doryab[2], Carola Voss[2], Christian Ketscher[1], Wulf Sienel[1], Teresa Kauke[1], Jan Michael Fertmann[1], Christian Schneider[1], Jürgen Behr[2 3], Martin Irmler[4 5], Johannes Beckers[4 5], Oliver Eickelberg[6], Benjamin Schubert[2], Stefanie Hauck[2 7], Rudolf Hatz[1 2], Otmar Schmid[2], Tobias Stöger[2], Herbert Schiller[2], Anne Hilgendorff[2 8]

[1]*Division for Thoracic Surgery Munich, Ludwig-Maximilians-University of Munich (LMU) and Asklepios Medical Center; Munich-Gauting, 82131, Gauting, Germany, Munich, Deutschland*, [2]*Institute for Lung Health and Immunity and Comprehensive Pneumology Center with the CPC-M bioArchive; Helmholtz Zentrum Munich; Member of the German Lung Research Center (DZL), 81377 Munich, Germany , Munich, Deutschland*, [3]*Department of Medicine V, University Hospital, LMU Munich and Asklepios Medical Center, Munich-Gauting, 82131, Gauting, Germany, Munich, Deutschland*, [4]*Helmholtz Zentrum München – German Research Center for Environmental Health (GmbH), Institute of Experimental Genetics, 85764 Neuherberg, Germany, Munich, Deutschland*, [5]*German Center for Diabetes Research (DZD), 85764 Neuherberg, Germany, Munich, Deutschland*, [6]*Division of Pulmonary Sciences and Critical Care Medicine, Department of Medicine, University of Colorado, Anschutz Medical Campus, Aurora, CO 80045, USA, Colorado*, [7]*Metabolomics and Proteomics Core, Helmholtz Center Munich, German Research Center for Environmental Health GmbH, 80939, Munich, Germany, Munich, Deutschland*, [8]*Center for Comprehensive Developmental Care at the iSPZ Hauner, Dr. von Haunersches Children’s University Hospital, Ludwig-Maximilians-University of Munich (LMU); Member of the German Lung Research Center (DZL), 81377 Munich, Germany , Munich, Deutschland*

**Background:**

Careful analysis of cellular function and differentiation in the human bronchial epithelium is important to understand, treat and prevent disease patterns that characterize chronic lung diseases (CLD, including chronic obstructive lung disease/ COPD). To enable the translational research in thoracic surgery, close collaboration between surgical departments and associated laboratories is warranted. A highly standardized long-term culture approach based on primary human bronchial epithelial cells (pHBECs) from non-CLD and end-stage COPD patients was recently developed in our laboratory (Stoleriu et al.,Thorax 2024). To physiologically mimic the native microenvironment of the bronchial epithelium further adaptations of primary cell culture are needed.

**Materials and methods:**

Over 200 human bronchial tissue samples from CLD patients undergoing lung transplantation at the Thoracic Surgery Division of the LMU Hospital Munich between 01.2018-09.2024 were processed at the CPC-M bioArchive, the LMU Hospital, the Asklepios Clinic Gauting and the Institute for Lung Health and Immunity Munich. Long-term pHBECs cultures isolated from non-CLD, COPD-II and IV patients at air-liquid-interface were used for comprehensive cell characterization and culture validation on a novel transwell system.

**Results:**

Single cell RNA-seq analysis of pHBECs from 2 non-CLD and 2 COPD-IV patients revealed a novel basal cell population that could drive the hypersecretory phenotype in end-stage COPD derived cultures. The basal cell state was characterized by overrepresentation of Wnt and Notch canonical airway pathways involved in secretory and multi-ciliated cell differentiation. After successful long-term pHBECs culture on conventional silicone-based membrane transwells, a novel culture system based on elasticporous convex transwell membranes was developed to improve cell differentiation, mimic physiological primary cell cultures and facilitate a reliable measurement of transcellular drug transport.

**Conclusion:**
The collaboration between thoracic surgery and respective scientific laboratories is able to update knowledge about the cellular landscape of the human bronchial epithelium and of the lung cell atlas, thus contributing to a better disease understanding in COPD patients driving therapeutic ideas.

Abstracts – DCK 2025 – 142nd Congress of the German Society of Surgery – Munich, March 26–28 • DOI 10.1515/iss-2025-2005
Innov Surg Sci 2025; 10 (Special Suppl 1): s256–s367

**Human Fingertip Regeneration follows clinical phases with distinct proteomic signatures – A comprehensive MS/MS analysis of human fingertip regeneration**
ID: 544

**Kategorie: SCF - Regeneration and Tissue Engineering**

Jurek Schultz[1], Leah Wissing[1], Purva Patel[2], Rita Aires[2], Patrick Glatte[1], Marc Gentzel[2], Adele Doyle[2], Tatiana Sandoval-Guzman[2], Guido Fitze[1]

[1]*Dresden, Technical University of Dresden, Pediatric Surgery, Dresden, Deutschland*, [2]*CRTD Zentrum für Regenerative Therapien TU Dresden, Dresden, Deutschland*

**Background:**
While fingertips are essential for hand function and aesthetics, they are among the most frequently injured parts of the human body. In parallel to the fantastic regenerative capacities of some animal models, distal injuries in humans can regenerate almost fully under semi-occlusive dressings, too. In contrast to limb regeneration in animal models and regular human wound healing, phases of human fingertip regeneration are hardly described, characterized, or analyzed on a cellular and molecular level.

**Materials and methods:**
A novel silicone finger cap, comprising a puncturable reservoir at the distal tip, was used to treat 22 human fingertip amputations. Wounds were clinically assessed and photographed while wound fluid samples were aspirated at regular intervals from the same patients. The regeneration process was divided into four typical clinical phases based on clinical appearances. Thirty-six wound fluids of different clinical phases were subjected to MS/MS; proteomic data was bioinformatically analyzed, including principal component analysis and hierarchical clustering.

**Results:**
In all included cases (n=17), subcutaneous tissue, nailbed, and skin regenerated with excellent outcomes. After initial hemostasis (coagulation, 12-24 hours), all wounds developed granulomatous tissue that proliferated into a hypergranuloma (hypergranulation 24h to 7 days). Later, keratinized epithelium proceeded to cover this tissue from the base of the wound (proliferation, 7 to 28 days). Finally, almost the entire regenerated fingertip was covered by regular skin (epithelialization, 28 to 42 days). These four clinical phases of regeneration displayed distinct proteomic signatures. At a false discovery rate (FDR) of 0.05, around one thousand marker proteins accounted for the clear proteomic separation of clinical stages, and various characteristic processes and networks could be found in each phase.

**Conclusion:**
Here, we conduct a longitudinal study to describe the clinical phases of human fingertip regeneration. We present comprehensive proteomic data that highlight the complex orchestration of these clinically and proteomically distinct phases. This study also shows for the first time that human fingertip regeneration can be studied from a proteomic perspective by analyzing wound fluids. Further analyses of this proteomic data and testing identified candidates in various settings will help unravel the obscure mechanisms orchestrating human fingertip regeneration. This is especially important since significant differences are overt compared to regenerative animal models.

**Unraveling MASH: Kupffer Cell-Driven Inflammation and Fibrosis within Hepatic Spheroids as a 3D Disease Model**
ID: 960

**Kategorie: SCF - Regeneration and Tissue Engineering**

Niklas Bogovic[1], Lydia Schneider[1], Marie Keller[1], Henrik Junger[1], Hans Schlitt[1], Elke Eggenhofer[1]

[1]*Uniklinik Regensburg, Klinik für Chirurgie, Regensburg, Deutschland*

**Background:**
Chronic inflammation and fibrosis are central to the progression of metabolic associated steatohepatitis (MASH), a condition increasingly modeled through hepatic spheroids for more accurate in vitro studies. Hepatic spheroids, comprising hepatocytes, stellate cells, Kupffer cells, and endothelial cells, replicate liver architecture and cell-cell interactions, offering insights into the pathophysiology of fibrosis and inflammation that are unattainable in traditional two-dimensional cultures.

**Materials and methods:**
Liver spheroids were generated in a 24-well plate format, containing a mixture of HepaRG cells, stellate cells, and endothelial cells, with and without the inclusion of Kupffer cells, to simulate various liver microenvironments. Immunohistochemical staining confirmed the presence of all cell types within the spheroids. To induce steatotic conditions, palmitic acid and oleic acid were added to a subset of spheroids, while another subset was treated with lipopolysaccharide (LPS) on day 3 to create MASH-like conditions. Cytotoxicity was measured using an LDH assay, and fibrosis and steatosis were evaluated through staining for collagen III, fibronectin, Sirius red (for fibrosis), and Nile red (for steatosis). Inflammatory cytokine levels (IL-6, IL-1β, and TNF-α) were quantified using ELISA and PCR, enabling comparisons between steatotic and MASH spheroids, as well as between spheroids with and without Kupffer cells, to assess variations in inflammatory and fibrotic responses.

**Results:**
Our findings demonstrate stable spheroid formation across conditions. Inflammatory cytokine levels, including IL-6, IL-1β, and TNF-α, were significantly elevated in MASH spheroids, particularly in those containing Kupffer cells. The LDH assay indicated an increase in LDH release in MASH spheroids with Kupffer cells, suggesting enhanced cytotoxicity or cell membrane damage under these conditions. This increase was notably higher compared to spheroids without Kupffer cells and those under steatotic conditions alone, highlighting the role of Kupffer cells in intensifying inflammatory and cytotoxic responses in the MASH model.

**Conclusion:**
The spheroid model, especially under MASH conditions, demonstrates significant promise for studying MASH pathogenesis by accurately mimicking chronic inflammation and fibrosis. The increased release of inflammatory cytokines and LDH in MASH spheroids containing Kupffer cells underscores the crucial role these cells play in driving inflammation and cellular stress. These findings suggest that Kupffer cells amplify liver injury in MASH, providing a useful model for evaluating potential therapeutic interventions targeting inflammation and fibrosis in NASH. This model not only enhances our understanding of disease mechanisms but also offers a platform for testing novel anti-inflammatory and antifibrotic treatments.

**Machine perfusion parameters to predict graft injury or early function after liver transplantation**
ID: 71

**Kategorie: SCF - Transplantation**

Nurith Winzen[1], Laura Malkus[1], Fuat Saner[1], Thomas Minor[1], Charlotte von Horn[1]

[1]*Universitätsklinikum Essen, Essen, Deutschland*

**Background:**
In line with the attempt to push the limits of acceptance criteria for donor organs, tools to support the surgeon with appropriate estimation of the quality of organ grafts are of growing importance.
Ex-vivo machine perfusion, that evolves as promising tool to improve graft integrity, may simultaneously be used for evaluation prior to transplantation.
As part of a randomized controlled trial, investigating the benefits of a short-term rewarming machine perfusion prior to liver transplantation, we also tried to evaluate a variety of biomarkers during machine perfusion as potential candidates to predict the ulterior fate of the graft after transplantation. The latter had been defined either by morphological injury to the graft as represented by maximal release of transaminases during the first 3 days after transplantation (TA max), or early functional recovery determined as maximum liver function capacity (LiMAx-test) during mechanical ventilation on postoperative day one.

**Materials and methods:**
Twenty livers from extended criteria donors, that had previously been accepted for transplantation, were subjected to machine perfusion with MPS solution for at least 90 min. Temperature was increased from 10°C to 20°C during the first 60 min. Data were obtained after 90 min of perfusion.

**Results:**
Significant correlations with peak transaminase levels after transplantation were found for perfusate levels of glucose, tissue inhibitor of metalloproteinase2 (TIMP2), AST, pH and lactate, with best correlations being evident for the product of TIMP2 and lactate ($r^2$=0.82; p<0.001).
No significant correlation was found for cold ischemia time (CIT), donor risk index (DRI), perfusate flow or hepatic release of flavin mononucleotides (FMN).
Most interestingly, early liver function (LiMAx) after transplantation did not significantly correlate with either of the above parameters. The best prognostic factor was again the product of TIMP2 and lactate which calculated a correlation factor of $r^2$=0.15 (p=0.10).
Actually, the obtained LiMAx values as readout of hepatic functional recovery did not correlate with peak transaminase levels as read out of morphological reperfusion injury with a computed coefficient of only $r^2$=0.15 (n.s.).

**Conclusion:**
Limitations of this investigation are to be seen in the relatively small number of patients as well as the rather homogeneous patient collective. The extension of the investigation towards transplantation of donor livers of lesser quality is, however, not an option in the human situation.
While there was a more than 20-fold difference between lowest and highest peak transaminase level after transplantation (185 to 3972 U/L), the variation with regard to the post-transplant LiMAx value were only 3-fold, representing a much smaller basis for the computed correlation coefficients.
On the other hand, function might not easily be prognosticated by predominantly non-functional injury parameters. The development and refinement of functional investigations of the organ during machine perfusion might hence be recommended.

### Sex-specific Differences in Gene Expression in Livers of Male and Female Fisher Rats

ID: 128

**Kategorie: SCF - Transplantation**

Simge Oral[1], Luisa Rajcsanyi[2], Bettina Budeus[3], Andrea Kindler-Röhrborn[2], Florian Vondran[4], Stefanie Flohé[5], Anke Hinney[2], Arzu Oezcelik[1]

[1]*Universitätsklinikum Essen, Klinik für Allgemein-, Viszeral-, Gefäß- und Transplantationschirurgie, Institut für Geschlechtersensible Medizin, Essen, Deutschland*, [2]*Universitätsklinikum Essen , Institut für Geschlechtersensible Medizin, Essen, Deutschland*, [3]*Universitätsklinikum Essen, Institut für Zellbiologie (Tumorforschung), Essen, Deutschland*, [4]*Universitätsklinikum Aachen, Klinik für Allgemein-, Viszeral- und Transplantationschirurgie, Aachen, Deutschland*, [5]*Universitätsklinikum Essen, Klinik für Unfall-, Hand- und Wiederherstellungschirurgie, Experimentelle Unfallchirurgie, Essen, Deutschland*

**Background:**
Sex-dependent differences in gene expression in the liver are not sufficiently studied. Especially after liver transplantation, differences at the molecular genetic level may have a significant influence on the outcome. Our aim was to assess sex specific differences in gene expression in Fisher rat liver tissues at the molecular level.

**Materials and methods:**
Male (n=5) and female (n=5) Fisher rats at the age of five months were euthanized and liver tissue was obtained. RNA was isolated and quality controlled. RNA-Seq was executed by NGS. Library creation was performed utilizing Lexogen 3' FWD method, sequencing was conducted using Illumina Sequencing. Data mapping and statistical analysis were performed with Enhanced Volcano on R. Kegg and GO analysis were conducted for gene enrichment.

**Results:**
We conducted a comprehensive analysis of the ten most significantly expressed genes between female and male rat livers ($p < 10^{-100}$, lowest p-value: $p < 10^{-250}$). Prominently expressed genes in female liver tissue are, among others involved in the eicosanoid and prostaglandin pathways. Marked expression of sex-specific sulfotransferases and CYP450 enzymes was notable. Other highly expressed genes in female liver tissue are associated with chemokine activity, macrophage differentiation, activation of PMNCs, NK cells, estrogen and blood coagulation. Differentially expressed genes in male liver tissue are, among others, involved in peroxidase, oxidoreductase and redox signaling pathway related genes.

**Conclusion:**
Our findings suggest that sex dependent differences in gene expression play an important role in pathways, that might influence the outcome after liver transplantation. Sex-specific gene expression analyses at different times after sex-mismatched and sex-matched liver transplantations might provide significant information, that can help to improve the outcome.

### Understanding the Effects of Donor Age and Prolonged Warm Ischemia Time on Liver Graft Viability with Normothermic Machine Perfusion studies in Sprague-Dawley Rats

ID: 446

**Kategorie: SCF - Transplantation**

Anna Pietsch[1], Leonard Boerger[1], Kilian Walter[1], Luca Padoan[1], Joseph Gassner[1 2], Johann Pratschke[1], Igor Maximilian Sauer[1], Felix Krenzien[1 2], Nathanael Raschzok[1 2], Simon Moosburner[1 2]

[1]*Charité – Universitätsmedizin Berlin, corporate member of Freie Universität Berlin and Humboldt-Universität zu Berlin, Department of Surgery , Experimental Surgery , Berlin, Deutschland*, [2]*Berlin Institute of Health at Charité – Universitätsmedizin Berlin, BIH Academy, Clinician Scientist Program, Berlin, Deutschland*

**Background:**

Liver transplantation faces a critical gap between organ demand and availability, leading to growing reliance on extended criteria donors (ECD), including those of advanced age and donation after circulatory death (DCD) [1]. Older donors and increased usage of DCD could offer considerable potential to expand the donor pool [2]. However, combining DCD status with advanced donor age may heighten susceptibility to ischemia-reperfusion injury (IRI), increasing the risk of complications and graft loss [3, 4]. Normothermic machine perfusion (NMP) has emerged as a promising alternative to static cold storage by maintaining near- physiologic conditions, enabling continuous viability assessment and reducing IRI [5, 6]. This study evaluates the combined impact of advanced donor age and prolonged warm ischemia time on graft viability using an ex vivo liver perfusion model in Sprague Dawley rats.

**Materials and methods:**

Twenty- four Sprague Dawley rats were assigned to 4 experimental groups differing in donor age (3 vs. 12 months) and NMP duration (3 vs. 6 hours). Following circulatory arrest and 30 minutes of warm ischemia time, livers were perfused using a pressure-dependent flow-controlled dual vessel NMP system, as previously described [7]. Verapamil was administered to prevent vasospasm. Bile was continuously collected. Perfusate and dialysate samples were analyzed for oxygenation and biochemical markers. Tissue samples were obtained for histological and proteomic analyses.

**Results:**

Livers from elderly donors were heavier than from younger ones (23.46g vs. 18.21g; $p<0.001$). Over time, perfusion pressures increased in both groups. Elderly livers required inflow reduction at later time points and maintained a more consistent target flow (83% vs. 33%). All livers cleared lactate with elderly grafts initially exhibiting higher levels (46 mg/dl vs. 65 mg/dl; $p=0.002$) but converging to the end (15 mg/dl vs. 17 mg/dl; $p>0.9$). Alanine- and Aspartate- Aminotransferase rose during perfusion. Younger livers showed significantly higher AST (4328 U/l vs. 3432 U/l; $p=0.041$) and Lactate- dehydrogenase levels throughout perfusion (21966 U/l vs. 20,650 U/l; $p>0,9$). Grafts from elderly donors produced more bile, with the highest production after 2 hours (84 mg/h vs. 39 mg/h; $p=0.044$).
Histological analysis showed liver injury across all groups, with no significant differences in necrosis or vacuolization, but younger livers presented more sinusoidal congestion. Prolonged perfusion increased liver injury, especially in older livers. No age-related changes in fibrosis were observed. However, older livers had a higher Kupffer cell density.
Younger livers displayed similar protein expression after 6 hours of perfusion as older livers after 3 hours. Prolonged perfusion led to greater proteomic divergence, particularly in older livers, which exhibited higher expression of oxidative stress- related proteins like Aldehyde dehydrogenase 1A1 and glutathione metabolism enzymes. Older livers also showed increased proteasome subunit expression and reduced mitochondrial ribosomal protein L55, indicating mitochondrial damage and increased protein degradation under cellular stress. Elevated Allograft Inflammatory Factor 1 was observed with prolonged perfusion, while Leukotriene C4 synthase expression was upregulated in older livers, indicating heightened immune activation associated with both extended perfusion time and higher donor age.

**Conclusion:**
Our research demonstrated the impact of donor age on liver graft function during NMP. Immunohistochemistry and proteomic analysis revealed heightened susceptibility to IRI in elder donor grafts with increased expression of proteins related to oxidative stress responses, mitochondrial damage, and inflammation, while perfusion performance and conventional viability markers did not clearly show age- related differences,
NMP proved efficiency in maintaining metabolic activity, indicating its potential utility for the assessment and preservation of ECD grafts. Implementing organ-specific therapies during NMP could further improve outcomes, particularly for older or DCD livers.

**References:**
1. Renz, J.F., et al., *Utilization of extended donor criteria liver allografts maximizes donor use and patient access to liver transplantation.* Ann Surg, 2005. 242(4): p. 556-63; discussion 563-5.
2. Moosburner, S., et al., *[High donor age for liver transplantation: Tackling organ scarcity in Germany].* Chirurg, 2019. 90(9): p. 744-751.
3. de Vera, M.E., et al., *Liver transplantation using donation after cardiac death donors: long-term follow-up from a single center.* Am J Transplant, 2009. 9(4): p. 773-81.
4. Okaya, T., et al., *Age-dependent responses to hepatic ischemia/reperfusion injury.* Shock, 2005. 24(5): p. 421-7.
5. Rodriguez, R.P., et al., *Outcome of Liver Transplants Using Donors After Cardiac Death With Normothermic Regional Perfusion.* Transplant Proc, 2022. 54(1): p. 37-40.
6. Ghinolfi, D., et al., *Pilot, Open, Randomized, Prospective Trial for Normothermic Machine Perfusion Evaluation in Liver Transplantation From Older Donors.* Liver Transpl, 2019. 25(3): p. 436-449.
7. Strobl, F., et al., *Advancing Perfusion Models: Dual-Vessel Ex Vivo Rat Liver Perfusion Based on a Clinical Setup.* Tissue Eng Part A, 2023. 29(19-20): p. 518-528.

**Extra-anatomical reconstruction of the portal vein during liver-retransplantation: a case report**
ID: 782

**Kategorie: SCF - Transplantation**

Isabelle Flammang[1], Felicia Kneifel[1], Felix Becker[1], Andreas Pascher[1], Philipp Houben[1]

[1]*University Hospital Münster, Münster, Deutschland*

**Background:**
Portal vein thrombosis is a challenge in liver transplantation. In case of chronic thrombosis, conventional end-to-end anastomosis is often not feasible, making extra-anatomical reconstruction necessary. This can be particularly difficult in the context of a retransplantation.

**Materials and methods:**
A 32 year-old patient was diagnosed with chronic graft failure with secondary sclerosing cholangitis following liver transplantation 5 years previously due to primary sclerosing cholangitis. Additionally, subtotal colectomy for refractory ulcerative colitis with simultaneous splenectomy for hypersplenism was performed 1 year previously. The retransplantation revealed a very thin-walled portal vein in the recipient. After creating an end-to-end anastomosis, a portal vein thrombosis became apparent shortly after reperfusion, so that a thrombectomy was necessary. The thrombectomy resulted in complete disintegration of the recipient's delicate vessel, meaning that a new anatomical portal vein anastomisation was not possible. Fortunately, a reno-portal jump graft could be created using an iliac donor vessel. To avoid collapse of the graft along its course between the pancreas and the posterior wall of the stomach, the jump graft was placed in a 16 mm ring-reinforced PTFE prosthesis. In addition, the placement of the arterial and biliodigestive anastomoses was unproblematic. Postoperative CT-scan revealed a stenosis of the portal-vein anastomosis due to kinking, so that a direct surgical correction with a change to a 20 mm ring-reinforced PTFE prosthesis was performed. After reoperation, a harmonious course of the jump graft with a good calibre was shown by CT-scan. Effective anticoagulation was started postoperatively using Argatroban-perfusor which was subsequently switched to oral anticoagulation with apixaban.

**Results:**
The patient showed an uncomplicated postoperative course, there was no allograft dysfunction with normal bilirubin on the first postoperative day and rapid regression of transaminases. Regular postoperative duplex sonography showed good portal venous flow and no evidence of recurrent thrombosis. The intensive care stay was 4 days, the hospital stay was 12 days. In the 10 month follow-up the patient was in good general condition with full allograft function. In the meantime, continuity after subtotal colectomy could be restored without any problems. Even after discontinuitation of oral anticoagulation, unrestricted portalvenous perfusion could be demonstrated by CT-scan.

**Conclusion:**
Portal vein reconstruction in retransplantation can be challenging and difficult. The technique demonstrated here, might be a valuable addition to the standard repertoire to reestablish portal inflow in liver transplantation.

### Depletion of donor dendritic cells ameliorates immunogenicity of both skin and hind limb transplants

ID: 880

**Kategorie: SCF - Transplantation**

Barbara Kern[1], Joerg Mengwasser[2], Anja Reutzel-Selke[3], Dietrich Polenz[3], Kirsten Führer[3], Steffen Lippert[3], Peter Tang[3], Edward Michaelis[4], Rusan Catar[5], Johann Pratschke[6], Christian Witzel[1], Igor Maximilian Sauer[3], Stefan Tullius[7], Muhammad-Imtiaz Ashraf[3]

[1]*Charité Universitätsmedizin Berlin, Chirurgische Klinik, Plastische Chirurgie, Berlin, Deutschland*, [2]*Medizinische Universität Hannover, Abteilung für Allgemein-, Viszeral-, Transplantationschirurgie, Hannover, Deutschland*, [3]*Charité Universitätsmedizin Berlin, Chirurgische Klinik, Experimentelle Chirurgie, Berlin, Deutschland*, [4]*DRK Kliniken Berlin Westend, Institut für Pathologie, Spandauer Damm 130, Deutschland*, [5]*Charité Universitätsmedizin Berlin, Abteilung für Nephrologie, Berlin, Deutschland*, [6]*Charité Universitätsmedizin Berlin, Chirurgische Klinik, Berlin, Deutschland*, [7]*Harvard Medical School, Division of Transplant Surgery, Boston*, *USA*

**Background:**
Acute cellular rejection remains a significant obstacle affecting successful outcomes of organ transplantation including vascularized composite tissue allografts (VCA). Cell-based therapies have demonstrated promising results with the potential to modify life-long conventional immunosuppression. Donor antigen presenting cells (APCs), particularly dendritic cells (DCs), orchestrate early alloimmune responses by activating recipient effector T cells. Of particular interest is the role of DCs as antigen-presenting cells and their potential tolerogenic effects as immature DCs.

**Materials and methods:**
Employing a targeted approach, we investigated the impact of donor-derived conventional DCs (cDCs) and APCs on the immunogenicity of skin and skin-containing VCA grafts, using major MHC-mismatched mouse models of skin and hind limb transplantation. For cDC depletion zDC-DTR knock-in donor mice received an intraperitoneal injection of diphteria toxin and transient depletion of all APCs was achieved by injecting Clodronate liposomes intravenously into donor C57BL/6J prior to transplantation. Transplant recipients went through an observation period of 6 days, alloimmune was assessed sequentially; graft survival, intragraft structural and inflammatory changes were tested via immune analyses.

**Results:**
By post-transplantation day 6, skin grafts demonstrated severe rejections, characterized by predominance of recipient CD4 T cells. In contrast, hind limb grafts showed moderate rejection, primarily infiltrated by CD8 T cells. Notably, the skin component exhibited heightened immunogenicity when compared to the entire VCA, evidenced by increased frequencies of pan ($CD11b^-CD11c^+$), mature ($CD11b^-CD11c^+MHCII^+$) and active ($CD11b^-CD11c^+CD40^+$) DCs and cDC2 subset ($CD11b^+CD11c^+$ $MHCII^+$) in the lymphoid tissues and the blood of skin transplant recipients. While donor depletion of cDC and APC reduced frequencies, maturation and activation of DCs in all analysed tissues of skin transplant recipients, reduction in DC activities was only observed in the spleen of hind limb recipients. Donor cDC and APC depletion did not impact all lymphocyte compartments, but significantly affected CD8 T cells and activated CD4 T cells in lymph nodes of skin recipients. Moreover, both donor APC and cDC depletion attenuated the Th17 immune response, evident by significantly reduced Th17 ($CD4^+IL\text{-}17^+$) cells in the spleen of skin recipients and reduced levels of IL-17E and lymphotoxin-α in the serum samples of both skin and hind limb recipients.

**Conclusion:**
In conclusion, our findings underscore the highly immunogenic nature of skin component in VCA. The depletion of donor APCs and cDCs mitigates the immunogenicity of skin grafts while exerting minimal impact on VCA.

**Moderate LMWH anticoagulation improves success rate of hind limb allotransplantation in mice**
ID: 895

**Kategorie: SCF - Transplantation**

Barbara Kern[1], Muhammad-Imtiaz Ashraf[2], Anja Reutzel-Selke[2], Joerg Mengwasser[3], Dietrich Polenz[2], Edward Michaelis[4], Johann Pratschke[5], Christian Witzel[1], Stefan Tullius[6], Igor Maximilian Sauer[2]

[1]*Charité Universitätsmedizin Berlin, Chirurgische Klinik, Plastische Chirurgie, Berlin, Deutschland*, [2]*Charité Universitätsmedizin Berlin, Chirurgische Klinik, Experimentelle Chirurgie, Berlin, Deutschland*, [3]*Medizinische Universität Hannover, Abteilung für Allgemein-, Viszeral-, Transplantationschirurgie, Hannover, Deutschland*, [4]*DRK Kliniken Berlin Westend, Institut für Pathologie, Berlin, Deutschland*, [5]*Charité Universitätsmedizin Berlin, Chirurgische Klinik, Berlin, Deutschland*, [6]*Harvard Medical School, Division of Transplant Surgery, Boston, USA*

**Background:**
The mouse hind limb model represents a powerful research tool in vascularized composite tissue allotransplantation, but its applicability is limited due to poor graft survival (62%-83%). Vascular thrombosis and massive hemorrhage are the major causes for these drop-outs. We hypothesize that because of better anticoagulation effect and lower risk of thrombocytopenia, application of low-molecular-weight-heparin (LMWH) will minimize vascular complications and enhance graft and animal survival.

**Materials and methods:**
Fifty allogeneic hind limb transplantations were performed (C57BL/6 to DBA/2 mice) using five different anticoagulation protocols. Bleeding and thromboembolic events were recorded macroscopically by postoperative hemorrhage and livid discoloration of the graft, respectively. Graft perfusion and survival were monitored daily by capillary-refill-time of graft toes within 2-3 seconds. Vascular congestion and tissue necrosis were examined by histological evaluation of hematoxylin-eosin-stained tissue sections.

**Results:**
Lorem All transplantations were technically successful. Increase in thromboembolic events and a concomitant decrease in bleeding events were observed with the decreasing concentration of heparin in the perfusion solution. While treatment of donor and recipient with low dose of LMWH could not reduce thromboembolic events, moderate dose effectively reduced these events. Compared to the poor outcome of graft perfusion with heparin alone, additional treatment of donor and recipient with low dose of LMWH improved graft and animal survival by 18%. Interestingly, animals treated with moderate dose of LMWH demonstrated 100% graft and animal survival.

**Conclusion:**
Treatment of donor and recipient mice with a moderate dose of LMWH prevents vascular complications and improves the outcome of murine hind limb transplants.

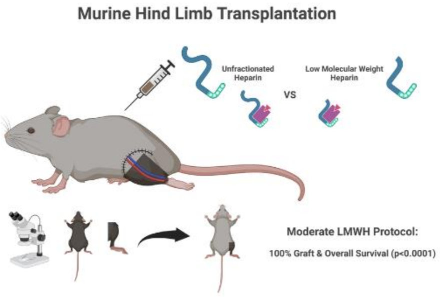

.
